# Identification, Replication, and Fine-Mapping of Loci Associated with Adult Height in Individuals of African Ancestry

**DOI:** 10.1371/journal.pgen.1002298

**Published:** 2011-10-06

**Authors:** Amidou N'Diaye, Gary K. Chen, Cameron D. Palmer, Bing Ge, Bamidele Tayo, Rasika A. Mathias, Jingzhong Ding, Michael A. Nalls, Adebowale Adeyemo, Véronique Adoue, Christine B. Ambrosone, Larry Atwood, Elisa V. Bandera, Lewis C. Becker, Sonja I. Berndt, Leslie Bernstein, William J. Blot, Eric Boerwinkle, Angela Britton, Graham Casey, Stephen J. Chanock, Ellen Demerath, Sandra L. Deming, W. Ryan Diver, Caroline Fox, Tamara B. Harris, Dena G. Hernandez, Jennifer J. Hu, Sue A. Ingles, Esther M. John, Craig Johnson, Brendan Keating, Rick A. Kittles, Laurence N. Kolonel, Stephen B. Kritchevsky, Loic Le Marchand, Kurt Lohman, Jiankang Liu, Robert C. Millikan, Adam Murphy, Solomon Musani, Christine Neslund-Dudas, Kari E. North, Sarah Nyante, Adesola Ogunniyi, Elaine A. Ostrander, George Papanicolaou, Sanjay Patel, Curtis A. Pettaway, Michael F. Press, Susan Redline, Jorge L. Rodriguez-Gil, Charles Rotimi, Benjamin A. Rybicki, Babatunde Salako, Pamela J. Schreiner, Lisa B. Signorello, Andrew B. Singleton, Janet L. Stanford, Alex H. Stram, Daniel O. Stram, Sara S. Strom, Bhoom Suktitipat, Michael J. Thun, John S. Witte, Lisa R. Yanek, Regina G. Ziegler, Wei Zheng, Xiaofeng Zhu, Joseph M. Zmuda, Alan B. Zonderman, Michele K. Evans, Yongmei Liu, Diane M. Becker, Richard S. Cooper, Tomi Pastinen, Brian E. Henderson, Joel N. Hirschhorn, Guillaume Lettre, Christopher A. Haiman

**Affiliations:** 1 Montreal Heart Institute, Montréal, Canada; 2 Department of Preventive Medicine, Keck School of Medicine and Norris Comprehensive Cancer Center, University of Southern California, Los Angeles, California, United States of America; 3 Program in Medical and Population Genetics, Broad Institute, Cambridge, Massachusetts, United States of America; 4 Divisions of Genetics and Endocrinology and Program in Genomics, Children's Hospital Boston, Boston, Massachusetts, United States of America; 5 Department of Human Genetics, McGill University and Genome Quebec Innovation Centre, Montreal, Canada; 6 Department of Preventive Medicine and Epidemiology, Loyola University Chicago Stritch School of Medicine, Maywood, Illinois, United States of America; 7 Department of Medicine, The Johns Hopkins GeneSTAR Research Program, The Johns Hopkins University School of Medicine, Baltimore, Maryland, United States of America; 8 Sticht Center on Aging, Wake Forest University School of Medicine, Winston-Salem, North Carolina, United States of America; 9 Laboratory of Neurogenetics, National Institute on Aging, National Institutes of Health, Bethesda, Maryland, United States of America; 10 NIH Intramural Center for Research on Genomics and Global Health, National Human Genome Research Institute, National Institutes of Health, Bethesda, Maryland, United States of America; 11 Department of Cancer Prevention and Control, Roswell Park Cancer Institute, Buffalo, New York, United States of America; 12 Department of Neurology, Boston University School of Medicine, Boston, Massachusetts, United States of America; 13 The Cancer Institute of New Jersey, New Brunswick, New Jersey, United States of America; 14 Division of Cancer Epidemiology and Genetics, National Cancer Institute, National Institutes of Health, Bethesda, Maryland, United States of America; 15 Division of Cancer Etiology, Department of Population Science, Beckman Research Institute, City of Hope, Duarte, California, United States of America; 16 International Epidemiology Institute, Rockville, Maryland, United States of America; 17 Division of Epidemiology, Department of Medicine, Vanderbilt Epidemiology Center, Vanderbilt University, Nashville, Tennessee, United States of America; 18 Vanderbilt–Ingram Cancer Center, Nashville, Tennessee, United States of America; 19 Human Genetics Center and Institute of Molecular Medicine and Division of Epidemiology, University of Texas Health Science Center, Houston, Texas, United States of America; 20 Division of Epidemiology and Community Health, University of Minnesota School of Public Health, Minneapolis, Minnesota, United States of America; 21 Epidemiology Research Program, American Cancer Society, Atlanta, Georgia, United States of America; 22 Department of Medicine, University of Mississippi Medical Center, Jackson, Mississippi, United States of America; 23 Laboratory of Epidemiology, Demography, and Biometry, National Institute on Aging, National Institutes of Health, Bethesda, Maryland, United States of America; 24 Sylvester Comprehensive Cancer Center and Department of Epidemiology and Public Health, University of Miami Miller School of Medicine, Miami, Florida, United States of America; 25 Cancer Prevention Institute of California, Fremont, California, United States of America; 26 School of Medicine and Stanford Cancer Center, Stanford University, Stanford, California, United States of America; 27 Department of Biostatistics, University of Washington, Seattle, Washington, United States of America; 28 Center for Applied Genomics, Children's Hospital of Philadelphia, Philadelphia, Pennsylvania, United States of America; 29 Department of Medicine, University of Illinois at Chicago, Chicago, Illinois, United States of America; 30 Epidemiology Program, Cancer Research Center, University of Hawaii, Honolulu, Hawaii, United States of America; 31 University of Mississippi Medical Center, Jackson, Mississippi, United States of America; 32 Department of Epidemiology, Gillings School of Global Public Health and Lineberger Comprehensive Cancer Center, University of North Carolina, Chapel Hill, North Carolina, United States of America; 33 Department of Urology, Northwestern University, Chicago, Illinois, United States of America; 34 Jackson Heart Study, Department of Medicine, Division of Cardiovascular Disease, University of Mississippi Medical Center, Jackson, Mississippi, United States of America; 35 Department of Biostatistics and Research Epidemiology, Henry Ford Hospital, Detroit, Michigan, United States of America; 36 Department of Epidemiology, University of North Carolina, Chapel Hill, North Carolina, United States of America; 37 Carolina Center for Genome Sciences, University of North Carolina, Chapel Hill, North Carolina, United States of America; 38 Department of Medicine, University of Ibadan, Ibadan, Nigeria; 39 Cancer Genetics Branch, National Human Genome Research Institute, National Institutes of Health, Bethesda, Maryland, United States of America; 40 National Heart, Lung, and Blood Institute (NHLBI), Division of Cardiovascular Sciences, National Institutes of Health, Bethesda, Maryland, United States of America; 41 Division of Sleep Medicine, Brigham and Women's Hospital, Boston, Massachusetts, United States of America; 42 Department of Urology, The University of Texas M. D. Anderson Cancer Center, Houston, Texas, United States of America; 43 Department of Pathology, Keck School of Medicine and Norris Comprehensive Cancer Center, University of Southern California, Los Angeles, California, United States of America; 44 Division of Epidemiology and Community Health, University of Minnesota, Minneapolis, Minnesota, United States of America; 45 Division of Public Health Sciences, Fred Hutchinson Cancer Research Center, Seattle, Washington, United States of America; 46 Department of Epidemiology, The University of Texas M. D. Anderson Cancer Center, Houston, Texas, United States of America; 47 The Johns Hopkins Bloomberg School of Public Health, Baltimore, Maryland, United States of America; 48 Institute for Human Genetics, Departments of Epidemiology and Biostatistics and Urology, University of California San Francisco, San Francisco, California, United States of America; 49 Department of Biostatistics and Epidemiology, Case Western Reserve University, Cleveland, Ohio, United States of America; 50 Department of Epidemiology, Graduate School of Public Health, University of Pittsburgh, Pittsburgh, Pennsylvania, United States of America; 51 Laboratory of Personality and Cognition, National Institute on Aging, National Institutes of Health, Baltimore, Maryland, United States of America; 52 Health Disparities Research Section, Clinical Research Branch, National Institute on Aging, National Institutes of Health, Baltimore, Maryland, United States of America; 53 Department of Medicine, Harvard Medical School, Boston, Massachusetts, United States of America; 54 Département de Médecine, Université de Montréal, Montréal, Canada; Queensland Institute of Medical Research, Australia

## Abstract

Adult height is a classic polygenic trait of high heritability (*h* ^2^ ∼0.8). More than 180 single nucleotide polymorphisms (SNPs), identified mostly in populations of European descent, are associated with height. These variants convey modest effects and explain ∼10% of the variance in height. Discovery efforts in other populations, while limited, have revealed loci for height not previously implicated in individuals of European ancestry. Here, we performed a meta-analysis of genome-wide association (GWA) results for adult height in 20,427 individuals of African ancestry with replication in up to 16,436 African Americans. We found two novel height loci (Xp22-rs12393627, *P* = 3.4×10^−12^ and 2p14-rs4315565, *P* = 1.2×10^−8^). As a group, height associations discovered in European-ancestry samples replicate in individuals of African ancestry (*P* = 1.7×10^−4^ for overall replication). Fine-mapping of the European height loci in African-ancestry individuals showed an enrichment of SNPs that are associated with expression of nearby genes when compared to the index European height SNPs (*P*<0.01). Our results highlight the utility of genetic studies in non-European populations to understand the etiology of complex human diseases and traits.

## Author Summary

Adult height is an ideal phenotype to improve our understanding of the genetic architecture of complex diseases and traits: it is easily measured and usually available in large cohorts, relatively stable, and mostly influenced by genetics (narrow-sense heritability of height *h* ^2^∼0.8). Genome-wide association (GWA) studies in individuals of European ancestry have identified >180 single nucleotide polymorphisms (SNPs) associated with height. In the current study, we continued to use height as a model polygenic trait and explored the genetic influence in populations of African ancestry through a meta-analysis of GWA height results from 20,809 individuals of African descent. We identified two novel height loci not previously found in Europeans. We also replicated the European height signals, suggesting that many of the genetic variants that are associated with height are shared between individuals of European and African descent. Finally, in fine-mapping the European height loci in African-ancestry individuals, we found SNPs more likely to be associated with the expression of nearby genes than the SNPs originally found in Europeans. Thus, our results support the utility of performing genetic studies in non-European populations to gain insights into complex human diseases and traits.

## Introduction

Adult height is a classic polygenic trait of high heritability (*h* ^2^∼0.8) [1], [2]. A recent large meta-analysis of genome-wide association (GWA) results for height, which included data from >180,000 individuals of European descent, identified 180 loci that associate with variation in height [3]. The most significantly associated variants at these loci explain approximately 10% of the variance, consistent with the hypothesis put forward in 1918 by Fisher on the “cumulative Mendelian factors”, which suggested that the segregation of a large number of genetic variants, each of small effect, is sufficient to explain the variation in height observed in humans [4].

In parallel to the work in European-ancestry populations, GWA studies for adult height in other ethnic groups, including Koreans, Japanese, Africans, and African Americans have also been performed [5]–[10]. The GWA scans in East Asians replicated several of the height loci already identified in individuals of European descent, and also found evidence for new height loci not previously implicated in individuals of European ancestry [6], [7]. The studies in Africans and African Americans were modest in size and, although they replicated nominally some of the associations previously found in European populations, were not well-powered to find new population-specific height loci [8], [9].

To search for novel loci for height in populations of African ancestry, and to explore systematically the replication of previously validated height loci, we combined GWA results for height from nine studies totaling 20,427 individuals of African descent. We identified two novel height loci and observed significant evidence for the replication of European height signals in African-derived populations. In fine-mapping of the European height loci we also identified variants that better define the association in individuals of African ancestry and control local gene expression in *cis* (*cis*-eQTLs), suggesting that they are likely to be better surrogates of the biologically functional alleles.

## Results/Discussion

The meta-analysis included results from nine studies: four population-based African-American studies (ARIC (N = 2,740), CARDIA (N = 699), JHS (N = 2,119), and MESA (N = 1,646)), one family-based African-American study (CFS (N = 386)), African-American GWA study consortia of breast (AABC (N = 5,380)) and prostate cancer (AAPC (N = 5,526)) and two case-control studies of obesity (Maywood (N = 743)) and hypertension (Nigeria (N = 1,188)) (Materials and Methods, Text S1 and Table S1). We tested associations between 3,310,998 genotyped or imputed SNPs and sex-, age-, and disease status-adjusted height Z-scores under an additive genetic model, correcting for global admixture using principal components (PCs) as covariates, and modeling family structure when appropriate (Text S1). Height results for each study were scaled using genomic control, and then combined using the inverse-variance meta-analytic method (Text S1).

**Figure 1:**
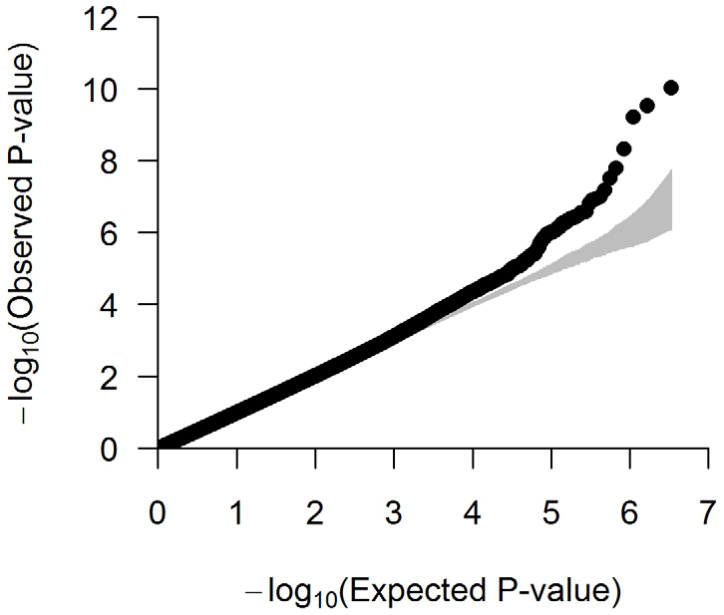
Quantile–quantile (QQ) plot of the meta-analysis with up to 3.3 M SNPs across 9 studies (N = 20,427). Each black circle represents an observed statistic for genotyped SNPs only (defined as the −log_10_ *P*) against the corresponding expected statistic. The grey area corresponds to the 90% confidence intervals calculated empirically using permutations. The individual studies' inflation factors, as well as the inflation factor of the meta-analysis, were corrected using genomic control. The inflation factor of the meta-analysis is λ_GC_ = 1.064.

The quantile-quantile (QQ) plot suggested little departure from the null expectation, except at the right end tail of the distribution (Figure 1). The associations that deviate most strongly from the null correspond to loci previously associated with height in European populations, providing a strong validation of our approach (Table 1). The overall inflation factor in the meta-analysis was λ_GC_ = 1.064 and results were again scaled using genomic control, a slightly conservative approach [11].

**Table 1:** SNPs that reached genome-wide significance (*P*<5×10^−8^) in the combined analysis.

				Discovery					Replication		Combined	
SNP	CHR(POS)	Locus	Effect allele/other allele	Effect allele frequency	Beta (SE)	GC-corrected P-value	Heterogeneity P-value	I^2^ (%)	Beta (SE)	P-value	Beta (SE)	P-value
rs2589113	2 (55369827)	*CCDC88A*	T/G	0.48	0.043 (0.009)	1.1×10^−5^	0.35	0	0.048 (0.014)	4.0×10^−4^	0.044 (0.008)	1.7×10^−8^
**rs4315565**	**2 (69141447)**	***ANTXR1***	**A/G**	**0.20**	**−0.067 (0.012)**	**1.5×10^−7^**	**0.83**	**0**	**−0.044 (0.018)**	**0.013**	**−0.059 (0.01)**	**1.2×10^−8^**
rs6440003	3 (142576899)	*ZBTB38*	A/G	0.81	0.064 (0.012)	3.7×10^−7^	0.48	0	0.066 (0.017)	1.3×10^−4^	0.065 (0.01)	1.9×10^−10^
rs724577	4 (17602508)	*LCORL*	A/C	0.31	0.054 (0.010)	1.2×10^−7^	0.28	0	0.091 (0.014)	1.9×10^−10^	0.067 (0.008)	1.1×10^−15^
rs994014	4 (82384814)	*PRKG2*	T/C	0.29	−0.052 (0.010)	1.1×10^−6^	0.85	0	−0.056 (0.015)	1.7×10^−4^	−0.054 (0.009)	7.8×10^−10^
rs9470004	6 (35449828)	*PPARD*	T/C	0.17	−0.078 (0.012)	6×10^−10^	0.20	9.9	−0.102 (0.035)	0.0033	−0.081 (0.012)	1.0×10^−11^
rs7979673	12 (64513524)	*HMGA2*	T/C	0.32	−0.056 (0.012)	6.7×10^−6^	0.6	0	−0.062 (0.015)	2.3×10^−5^	−0.058 (0.01)	7.3×10^−10^
rs2351491	15 (87199109)	*ACAN*	T/C	0.25	0.062 (0.011)	1.1×10^−7^	0.42	0	0.048 (0.017)	0.0034	0.057 (0.01)	1.5×10^−9^
rs11658329	17 (59116763)	*MAP3K3*	C/G	0.7	0.049 (0.010)	4.2×10^−6^	0.36	0	0.063 (0.015)	2.5×10^−5^	0.053 (0.009)	5.7×10^−10^
rs1787200	18 (44841652)	*DYM*	A/G	0.37	0.044 (0.010)	1.5×10^−5^	0.92	0	0.070 (0.014)	5.5×10^−7^	0.053 (0.008)	1.2×10^−10^
**rs12393627**	**X** **(2895723)**	***ARSE***	**A/G**	**0.63**	**−0.093 (0.019)**	**1.4×10^−6^**	**0.24**	**0**	**−0.064 (0.012)**	**2.6×10^−7^**	**−0.072 (0.010)**	**5.7×10^−12^**

For loci with more than one SNP with a *P*<5×10^−8^, we list the SNP with the smallest combined P-value. Results for the 153 SNPs that were followed up by *in silico* replication are available in Table S2. We highlight in bold the two loci not previously implicated in the regulation of height in European-ancestry populations. Effect size (beta) and standard error (SE) are in Z-score units. The effect allele frequency is the average frequency across all African-American discovery studies. The heterogeneity P-value is based on Cochran Q heterogeneity test. *I* ^2^ is a measure of heterogeneity and represents the proportion of inconsistency in individual studies that cannot be explained by chance.

Two genomic loci (*LCORL* on chromosome 4 and *PPARD* on chromosome 6), previously implicated in height in European populations [3], reached genome-wide significance in the discovery meta-analysis (*P*<5×10^−8^; Table 1, Figure S1 and Table S2). We prioritized 153 SNPs with *P*<1×10^−5^ from our meta-analysis for *in silico* replication in up to 16,436 African Americans from five additional studies (Text S1). After combining the data in a joint analysis, 40 SNPs from 11 different chromosomal regions reached genome-wide significance (Table 1 and Table S2), including two SNPs not previously implicated in the regulation of height: rs12393627 on the X-chromosome and rs4315565 on chromosome 2 (Table 1).

**Figure 2:**
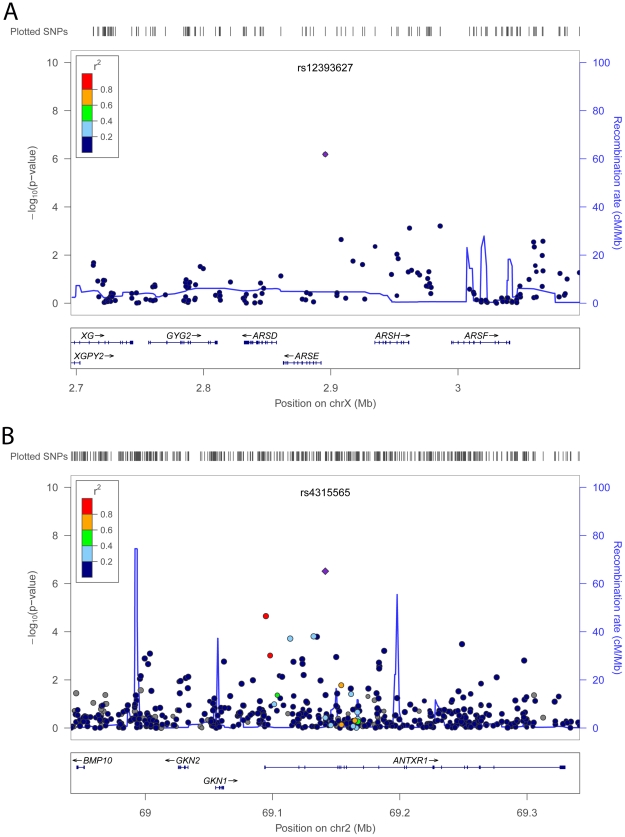
SNPs are plotted using LocusZoom [**22**] by position on the chromosome against association with adult height (−log_10_ *P*). The SNP name shown on the plot was the most significant SNP after the discovery meta-analysis. Estimated recombination rates (from HapMap) are plotted in cyan to reflect the local LD structure. The SNPs surrounding the most significant SNP are color coded to reflect their LD with this SNP (taken from pairwise *r* ^2^ values from the ARIC African Americans Affymetrix6.0 dataset for rs1239627 (A) and from the HapMap YRI data for rs4315565 (B)). The size of the points on the plots is proportional to the number of individuals with available genotype for any given SNP. Genes, the position of exons and the direction of transcription from the UCSC genome browser are noted. Hashmarks represent SNP positions available in the meta-analysis.

rs12393627 is located 3.2 kb upstream of the arylsulfatase E (*ARSE*) gene on chromosome Xp22 (Figure 2a). Mutations in the *ARSE* gene cause X-linked brachytelephalangic chondrodysplasia punctata (*CDPX1*; OMIM #302950), a congenital disorder of bone and cartilage development also characterized by short stature [12]. The co-localization of human growth syndrome genes with SNPs associated with adult height has been reported in European-ancestry samples [3], [13], [14]. rs12393627 reached a *P* = 1.4×10^−6^ in the initial meta-analysis (N = 8,333; the SNP was not on the genotyping arrays and/or could not be imputed for AABC, AAPC, Maywood, and Nigeria), and was strongly replicated for association with height in 13,153 African Americans (replication *P* = 2.6×10^−7^; combined *P* = 5.7×10^−12^) (Table 1). When considering the number of independent markers in a 1 Mb window we found no secondary independent signals in the region conditioning on genotype at rs12393627. We also found no significant evidence of heterogeneity at rs12393627 between men and women (*P* = 0.26).

The derived A-allele (i.e. non-ancestral allele based on the chimp genome) at rs12393627 is monomorphic in the HapMap CEU individuals and has a frequency of 54% in the HapMap YRI participants. We also investigated the association of rs12393627 with height in 3,487 Japanese Americans and 2,979 Latinos from the Multiethnic Cohort (MEC) (Text S1). Whereas the marker was monomorphic in Japanese Americans, the association between height and rs12393627 was replicated in Latinos with a comparable effect size (A-allele frequency = 97%, standardized effect size  = −0.177±0.088, *P* = 0.044). The frequency of this allele is consistent with previous estimates of ∼5–10% African ancestry among Latinos in the MEC [15]. Measures of local ancestry (the number of European-derived chromosomes (0, 1, or 2) in each individual) were not available for the X-chromosome, but since the marker is polymorphic only in African-derived populations (according to HapMap phase 3 data [16]), the height association signal defined by rs12393627 on Xp22 is likely to be specific to these populations.

SNP rs4315565 on 2p14 (discovery *P* = 1.5×10^−7^; combined *P* = 1.2×10^−8^) is located in intron 3 of the anthrax toxin receptor 1 (*ANTXR1*) gene, and 189 kb upstream of the bone morphogenetic protein 10 (*BMP10*) gene (Figure 2b), a member of the TGF-β signaling pathway. This pathway is important in normal skeletal growth [17] and implicated in previous GWA studies of height [3]. We observed no evidence of heterogeneity by sex (*P* = 0.34) and no independent signals when conditioning on rs4315565 within a 1 Mb window.

The allele frequency of rs4315565 differs strongly between the HapMap CEU and YRI samples: the derived A-allele, which is associated with decreased height, has a frequency of 85% in CEU and 2% in YRI, respectively (F_st_ = 0.701). This allele frequency difference is consistent with recent weak positive selection acting in individuals of European ancestry (iHS = −1.668) [18], and could indicate an association with local ancestry. In a conditional analysis where we controlled for global ancestry using PCs as covariates, we did observe a significant association between height and local ancestry at the *ANTXR1* locus, with an increase in the number of European chromosomes associated with a decrease in height (*P* = 1.6×10^−6^; N = 18,495 samples available for this analysis) [19]. Still controlling for global ancestry with PCs, genotypes at rs4315565 could account for the association between local ancestry and height (*P* = 0.22 for local ancestry conditional on rs4315565), while the association of rs4315565 with height diminished but remained significant in the same model (*P* = 4.6×10^−8^ and *P* = 0.0044, before and after conditioning on local ancestry; N = 18,495).

To investigate the relationship between rs4135565 and local ancestry further, we considered the background on which the rs4135565 variants were present in different individuals. In analyses stratified by the number of African/European chromosomes in the region, rs4315565 was nominally associated with height in African Americans that are homozygous (*P* = 0.038) or heterozygous (*P* = 0.043) for African chromosomes (with effect size stronger in African chromosome homozygotes) (Table 2). In 1,188 Nigerians from the discovery phase, a similar trend between height and rs4315565 was observed (*P* = 0.075). rs4315565 was not significantly associated with height in African Americans that are homozygous for European chromosomes at the locus (*P* = 0.91), although the sample size of this sub-group is small (N = 943) (Table 2). More strikingly, this variant is not associated with height in populations of European ancestry in the GIANT Consortium (N = 133,653, *P* = 0.66) [3]. Together, these results suggest that 2p14 harbors at least one novel height-associated variant that is strongly associated with African ancestry and is correlated with rs4315565 in African- but not European-derived chromosomes. Our results also indicate that rs4315565 is a better marker of the functional variant(s) than is local ancestry or any other SNPs represented in HapMap.

**Table 2:** Stratified analysis of the association between adult height and rs4315565 based on ancestry at the locus.

Ancestry	N	Beta (SE)	P-value
African homozygote	11,608	−0.067 (0.032)	0.038
African/European heterozygote	5,971	−0.052 (0.026)	0.043
European homozygote	943	−0.007 (0.056)	0.90
Combined analysis	18,522	−0.051 (0.019)	0.0079

The effect size (beta) and standard error (SE) are in Z-score units. The direction of the effect is given for the A-allele at rs4315565. Results are from a meta-analysis of the results in ARIC, CARDIA, CFS, JHS, MESA, AABC, and AAPC. Because of its small sample size, we could not analyze height in the CFS participants homozygous for the European chromosome.

We then considered the previously known height loci. Of the 180 SNPs previously reported by the GIANT Consortium to be associated with height in populations of European ancestry, the effect estimates for 38 SNPs were in the same direction as the initial report and nominally associated (*P*<0.05) with height in the African-derived height meta-analysis. This number is however a lower-bound estimate of the number of known European height loci that replicate in individuals of African ancestry because it does not take into account different LD relationships in European and African chromosomes: since any of the SNPs in LD in European-ancestry individuals with the GIANT height SNPs could be causal, this entire set of SNPs need to be evaluated, both in terms of statistical significance and direction of effect, for replication in the African height meta-analysis. To address this issue, we utilized a rigorous framework, described in the Materials and Methods section and graphically summarized in Figure S2, to test systematically for replication at the previously known European height loci in the African meta-analysis. We started with 161 of the 180 height SNPs identified by the GIANT Consortium (19 SNPs could not be tested because linkage disequilibrium (LD) information in HapMap was not available) [3], and generated 5,819 sets of 161 SNPs matched on minor allele frequency using the HapMap2+3 CEU dataset. We then counted the number of SNPs (also considering LD proxies) in the African height meta-analysis with directionally consistent (one-tailed) *P*≤0.05 for the set of 161 height-associated SNPs and the simulated sets. We found one simulation with a count of nominal associations equal to or higher than what we observed for the 161 height-associated SNPs (*P* = 1.7×10^−4^; 171 nominal associations for the GIANT height SNPs (and their proxies); median number of nominal associations to height in the matched sets of SNPs = 28 (range = 8–172)). Therefore, we found strong overall evidence of replication in our large meta-analysis of 20,427 individuals of African ancestry for SNPs previously associated with adult height in individuals of European ancestry, indicating a substantial shared genetic basis for height in populations separated since the out-of-Africa event.

**Figure 3:**
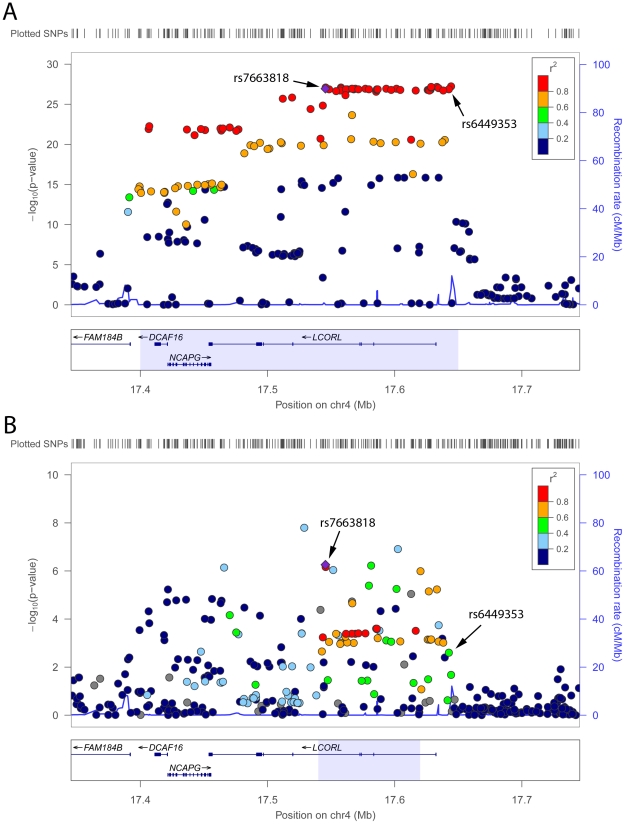
Height association results. In Europeans from the GIANT Consortium (A) [3] and in individuals of African ancestry (B) (this study) at the *LCORL* locus on chromosome 4. The GIANT Consortium originally reported SNP rs6449353, whereas rs7663818 was fine-mapped in the African height meta-analysis. For each panel, the light blue box corresponds to the chromosomal interval flanked by the leftmost and rightmost SNPs with a *r* ^2^≥0.8 with rs7663818 in HapMap CEU (A) and YRI (B) participants: these intervals are 250 kb and 80 kb wide in CEU and YRI, respectively.

The replication procedure described above also allowed us to identify, for each of the 161 European height loci that we assessed using data from our African meta-analysis, the best candidate height index SNP (Table 3 and Table S3). For instance in population of European ancestry at the *LCORL* locus on chromosome 4, the GIANT height SNP (rs6449353) and the SNP identified by fine-mapping in the African height meta-analysis (rs7663818) are both strongly associated with height (*P*<1×10^−25^) and in strong LD (*r* ^2^>0.8) with each other (Figure 3a). However, in African-derived populations, LD is weaker between the two SNPs (*r* ^2^<0.6) and the association with height is stronger for rs7663818 (*P* = 2.9×10^−7^) than for rs6449353 (*P* = 0.0025) (Figure 3b). When we consider SNPs in strong LD (*r* ^2^>0.8) with rs7663818 in HapMap CEU and YRI populations, they define genomic intervals of 250 kb and 80 kb, respectively (light blue boxes in Figure 3). Finally, in lymphoblastoid cell lines derived from YRI individuals (Materials and Methods), rs7663818, but not rs6449353, is associated with *LCORL* gene expression levels (*LCORL* eQTL *P* = 0.0026 and *P* = 0.13 for rs7663818 and rs6449353, respectively). Thus, the *LCORL* locus illustrates a clear example of the utility of fine-mapping association signals in other ethnic groups, both in terms of narrowing the genomic interval of interest and highlighting potential functional variants (*cis*-eQTL).

**Table 3:** Fine-mapping results for SNPs associated with height in individuals of European descent [3].

Association results in the African-American height meta-analysis for the SNPs associated with height in European-ancestry populations						Fine-mapping results in the African-American height meta-analysis of the height association signals in European-ancestry populations					
European height SNP	Chr (BP)	Effect allele/Other allele	Beta (SE)	GC-corrected P-value	Annotation	Best African-American SNP	Chr (BP)	Effect allele/Other allele	Beta (SE)	GC-corrected P-vlaue	Annotation
rs9428104	1 (118657110)	A/G	−0.0187 (0.0102)	0.076	*SPAG17* (intergenic, 128 kb)	rs7536458	1 (118666125)	G/T	0.0465 (0.0121)	9.6×10^−5^	*SPAG17* (intergenic, 137 kb)
rs3791675	2 (55964813)	T/C	−0.0312 (0.0221)	0.17	*EFEMP1* (intron)	rs1346789	2 (55945556)	A/G	0.0765 (0.0192)	5.6×10^−5^	*EFEMP1* (intergenic, 1 kb)
rs12470505	2 (219616613)	T/G	0.0232 (0.0096)	0.019	*CCDC108* (intergenic, 2 kb)	rs4453686	2 (219604794)	C/G	0.0395 (0.0094)	2.7×10^−5^	*CCDC108* (intron)
rs9835332	3 (56642722)	C/G	−0.0401 (0.0126)	0.0021	*C3orf63* (missense)	rs7637449	3 (56603071)	A/G	0.0597 (0.0128)	3.1×10^−7^	*CCDC66* (missense)
rs724016	3 (142588260)	A/G	−0.0485 (0.0107)	1.1×10^−5^	*ZBTB38* (intron)	rs2871960	3 (142604504)	A/C	−0.0655 (0.0123)	1.3×10^−7^	*ZBTB38* (intron)
rs6449353	4 (17642586)	T/C	0.0312 (0.01)	0.0025	*LCORL* (intergenic, 10 kb)	rs7663818	4 (17545541)	C/T	−0.0489 (0.0095)	2.9×10^−7^	*LCORL* (intron)
rs17081935	4 (57518233)	T/C	0.0021 (0.0142)	0.89	*C4orf14* (intergenic, 6 kb)	rs17087335	4 (57533340)	G/T	0.0447 (0.0117)	0.00011	*C4orf14* (intron)
rs788867	4 (82369030)	T/G	−0.029 (0.0158)	0.076	*PRKG2* (intergenic, 24 kb)	rs1662840	4 (82375433)	A/G	0.0513 (0.0102)	5.6×10^−7^	*PRKG2* (intergenic, 30 kb)
rs6470764	8 (130794847)	T/C	−0.0392 (0.0111)	0.00061	*GSDMC* (intergenic, 35 kb)	rs4733724	8 (130792910)	A/G	0.0442 (0.0113)	7.6×10^−5^	*GSDMC* (intergenic, 37 kb)
rs12680655	8 (135706519)	C/G	0.0212 (0.0095)	0.031	*ZFAT* (intron)	rs2277138	8 (135691822)	A/G	0.0458 (0.0121)	0.00012	*ZFAT* (intron)
rs8181166	9 (88306448)	C/G	0.0199 (0.0128)	0.13	*ZCCHC6* (intergenic, 147 kb)	rs405761	9 (88274946)	C/T	0.0372 (0.01)	0.00016	*ZCCHC6* (intergenic, 116 kb)
rs1468758	9 (112846903)	T/C	−0.0208 (0.0135)	0.14	*LPAR1* (intergenic, 7 kb)	rs10817133	9 (112831795)	A/G	0.0463(0.0129)	0.00025	*LPAR1* (intron)
rs2145998	10 (80791702)	A/T	−0.0339 (0.0094)	0.00048	*PPIF* (intergenic, 7 kb)	rs941873	10 (80809468)	C/T	−0.0541 (0.0103)	1.9×10^−7^	*ZCCHC24* (intergenic, 3 kb)
rs634552	11 (74959700)	T/G	0.0159 (0.0099)	0.12	*SERPINH1* (intron)	rs606452	11 (74953826)	A/C	0.0425 (0.01)	1.8×10^−5^	*SERPINH1* (intron)
rs1351394	12 (64638093)	T/C	0.0392 (0.0093)	4.7×10^−5^	*HMGA2* (intron)	rs7968682	12 (64658147)	G/T	−0.0426 (0.0093)	4.7×10^−6^	*HMGA2* (intergenic, 12 kb)
rs862034	14 (74060499)	A/G	−0.0317 (0.0121)	0.011	*LTBP2* (intron)	rs862057	14 (74048618)	A/G	0.04 (0.0095)	2.3×10^−5^	*LTBP2* (intron)
rs4821083	22 (31386341)	T/C	0.04 (0.0094)	3.7×10^−5^	*SYN3* (intron)	rs3788478	22 (31387746)	A/G	0.0414 (0.0094)	1.0×10^−5^	*SYN3* (intron)

In the left-handed side of the table, we present the association results in the African-American height meta-analysis for SNPs associated with height in European-ancestry individuals. In the right-handed side of the table, we present results from our fine-mapping experiment using data from our African-American height meta-analysis. Only SNPs with *P*<3×10^−4^ (Bonferroni correction threshold; α = 0.05/161 SNPs) are presented here; the complete list of 161 SNPs (19 of the 180 height SNPs from the GIANT Consortium were not available for fine-mapping) is in Table S3. For intergenic SNPs, we provide the closest gene and the physical distance between them.

For 40 loci, the index SNPs from our fine-mapping list was nominally associated with height (*P*<0.05) in the African height meta-analysis, whereas the corresponding index European height SNPs was not. To test whether this result reflects an enrichment of surrogates for functional variants identified by fine-mapping, we designed an experiment using allelic gene expression phenotypes in the HapMap YRI cell lines as functional readouts. We hypothesized that if our trans-ethnic fine-mapping strategy was successful, a larger fraction of variants in the list of fine-mapped height SNPs should be associated with phenotypes (in this case gene expression) than of variants in the list of European index height SNPs. In other words, the list of SNPs from our fine-mapping experiment should contain more *cis*-eQTLs than the GIANT list of height SNPs in cell lines derived from Africans. We retrieved allelic expression mapping datasets from the HapMap YRI cell lines (Materials and Methods) and observed that 4.7% of the GIANT index height SNPs and 8.6% of the best candidate height SNPs obtained by trans-ethnic fine-mapping, were both nominally associated with height (*P*<0.05) in our meta-analysis and with allelic expression phenotypes (*P*<0.01). When we used simulations to assess the significance of these results, we found no simulated set with a *cis*-eQTL enrichment equal or above that observed in the data (*P*<0.01, obtained from 100 simulations (Text S1)). Therefore, fine-mapping European height loci in African-ancestry individuals generated a list of markers more likely to control gene expression, potentially improving mechanistic insights into the biology of height. Although we did not see an enrichment when compared to the list of GIANT index height SNPs, we also found that 17 missense SNPs are in strong LD (*r* ^2^≥0.8 based on HapMap phase II YRI) with the fine-mapped height SNPs (Table S4).

In conclusion, our study shows the benefit of performing large-scale genetic studies in non-European populations to discover new biology (we identified two novel height loci), and to gain functional insights at the loci previously found in European-derived individuals (in this case, by enrichment of *cis*-eQTL signals). The strong replication of most of the European height loci in African-ancestry populations suggest that many of the published association signals with common variants from GWA studies – for height and perhaps other complex diseases and traits – are relevant across different populations and caused by shared genetic factors that predate the out-of-Africa event.

## Materials and Methods

### Ethics statement

All participants gave informed written consent. The project has been approved by the local ethics committees and/or institutional review boards.

### Studies

Five discovery studies/consortia (AABC, AAPC, CARe, Maywood, and Nigeria) and five replication studies (GeneSTAR, HANDLS, Health ABC, WHI, and MEC) contributed height association results to this project. There were eight population-based cohorts (ARIC (N = 2,740), CARDIA (N = 699), JHS (N = 2119), MESA (N = 1,646), HANDLS (N = 993), HABC (N = 1,139), WHI (N = 8,149) and MEC (N = 11,569)), two family-based cohorts (CFS (N = 386) and GeneSTAR (N = 1,148)) two case-control studies (Maywood (obesity, N = 743) and Nigeria (hypertension, N = 1,188)), and two cancer consortia comprised of case-control studies that were population-based or nested within prospective cohorts (AABC (breast cancer, N = 5380), AAPC (prostate cancer, N = 5,526). All cohorts with genome-wide genotyping data available were genotyped on the Affymetrix 6.0 array, except AABC, AAPC, HANDLS, HABC and GeneSTAR, that were genotyped on the Illumina 1M-duo or 1Mv1_c chip. The studies, including genotyping and quality control steps, are described in detail in Text S1. The statistics (height and age) are summarized in Table S1. Genotype imputation was performed as previously described [20] and is summarized in Text S1.

### Statistical analysis

Height measures were corrected for sex, age, disease status, and other appropriate covariates (e.g. recruitment centers), and were normalized into Z-scores (Text S1). Association analysis was performed using linear regression for studies of unrelated individuals and a linear mixed effect model for family-based studies, testing an additive model and including the 4–10 first principal components. Results were combined using the inverse variance meta-analysis method. Local ancestry was estimated using the HAPMIX software using default parameters [19]. Conditional analyses were performed by including SNP genotypes or local ancestry estimates in the linear models.

### Replication of European height loci in African Americans

The list of European height loci from the largest study to date was used as a source of known European loci for fine-mapping [3]. The procedure is graphically summarized in Figure S2. Of the 180 SNPs from this list, 19 were filtered for lack of available LD data (we combined data from HapMap2 haplotype release 22 (Aug 2007), HapMap3 haplotype release 2 (Jul 2009), and HapMap2+3 LD data release 27 (Apr 2009); conflicting data, as is the case for these 19 SNPs, were excluded). LD estimates (*r* ^2^) from CEU HapMap 2+3 were used to generate the set of common SNPs (proxies) tagging the remaining putative loci (*r* ^2^≥0.8). These sets were then binned using YRI HapMap 2+3 LD as follows: the whole list of proxies was randomized, to remove any bias towards significance in the representative P-values; the first SNP was removed and set as an “index” SNP; then all SNPs not yet binned were filtered based on LD (*r* ^2^≥0.3) with the index SNP. This procedure was repeated until all SNPs were binned. The metric for replication of a European signal was the number of SNP bins nominally significant (*P*≤0.05), and replication of the entire list of known SNPs was the number of significant bins across all loci. Each SNP bin was represented by the index SNP used to generate it. Because the SNPs are in LD with known European signals, there is a strong prediction as to which index SNP allele should be increasing height: it should be the allele in LD with the height-increasing allele in Europeans. Therefore, all index SNP P-values were made one-tailed (set to *P*/2 or 1-*P*/2) based on the hypothesis that the height-increasing allele should be the one predicted by the European SNP, based on the phased HapMap CEU data.

The LD thresholds used for proxy determination in European ancestry and binning in African ancestry were arbitrary and likely do not fully encompass the LD structure of the populations in this meta-analysis. To control for artifacts introduced by these thresholds and the HapMap data, 5,819 sets of 161 SNPs, matched to the European known loci on HapMap 2+3 CEU minor allele frequency, were generated. Since the European SNP list contains independent loci, each simulated list was designed to contain relatively independent SNPs (CEU *r* ^2^≥0.2); changing this threshold did not alter the results. The same procedure of proxy generation and SNP binning (see Figure S2 for a graphical description of the binning strategy) was performed on each of the 5,819 sets to generate a null distribution of significant bins.

To generate the list of “best” SNP for each locus (fine-mapped list), the binning procedure was repeated for the known SNPs, except each iteration selected an index SNP from the list of remaining SNPs, sorted on P-value, not randomized. Note that the best SNPs at each locus are not perfectly concordant between Table 1 and Table 3 because our fine-mapping approach did not consider the *in silico* replication data and required that the SNPs are available in the HapMap phased haplotypes. We note that our fine-mapping approach focuses on SNPs with low P-values and is thus more likely to identify markers with fewer missing genotypes, that is markers for which we have more statistical power.

### Analysis of *cis*-acting eQTLs

To assess whether European SNPs replicated for height (at nominal *P*<0.05) in African-ancestry populations would also be more likely to show links to functional variation in samples of African ancestry, we applied a sensitive technique for mapping *cis*-regulatory allelic expression SNPs [21] in lymphoblastoid cell lines (LCLs) derived from 56 unrelated Yoruba HapMap participants. A detailed description of the protocols and statistical methods used is available in the Text S1.

## Supporting Information

Figure S1 Manhattan plot of the height meta-analysis (3,310,998 SNPs in up to 20,809 participants from 9 studies). The dashed line highlights the genome-wide significance threshold used in this study (*P*<5×10^−8^). In the discovery phase of the project, SNPs at 4 loci reached genome-wide significance: *LCORL* on chromosome 4, *PPARD* on chromosome 6, *SULF1* on chromosome 8, and *ACAN* on chromosome 15. The association between height and SNPs near *SULF1* did not replicate. The 3 remaining loci – *LCORL*, *PPARD*, and *ACAN* – are loci previously associated with height in Europeans. Genomic-control P-values are displayed.
(TIF)

Figure S2 On the left, an example analysis for the European SNP rs12470505 (CCDC108). Top: rs12470505 (square) and proxies (circles; *r* ^2^≥0.8 in HapMap2+3 CEU), plotted with their P-values in the GIANT European analysis. Bottom: the same SNPs, plotted with African-American meta-analysis P-values, converted to one-tailed P-values based on predicted direction of effect from the European result and phased HapMap2 CEU data. Colors segregate SNPs into 6 randomly seeded “independent” clusters (*r* ^2^≥0.3) using HapMap2+3 YRI linkage disequilibrium estimates. Right: simulation results for the fine-mapping analysis. Simulations were matched to the European SNP list by minor allele frequency; SNPs in each simulation were independent of each other at *r* ^2^≥0.2 in HapMap2+3 CEU. The result for each simulation is significant bins/total bins. Red line indicates observed proportion of significant bins for true European SNP replication (*P* = 8.6×10^−6^).
(TIF)

Table S1 Baseline characteristics of cohorts involved in the study.
(DOC)

Table S2 Association results for the top 153 SNPs in the discovery meta-analysis. Positions are on NCBI build 36.1 (hg18) and the alleles are on the forward strand. Beta (effect size) and SE (standard error) are in standardized ‘Z-score’ units.
(DOC)

Table S3 Fine-mapping results for SNPs associated with height in Caucasians [49]. We could not fine-map 19 of the 180 SNPs reported by the GIANT Consortium because they were not available in the HapMap phased datasets. In the left-handed side of the table, we present the association results in the African-American height meta-analysis for SNPs associated with height in Caucasians. In the right-handed side of the table, we present results from our fine-mapping experiment using data from our African-American height meta-analysis. For intergenic SNPs, we provide the closest gene and the physical distance between them.
(DOC)

Text S1 Supplementary information.
(DOC)
