# Correction: Identification, Replication, and Fine-Mapping of Loci Associated with Adult Height in Individuals of African Ancestry

**DOI:** 10.1371/annotation/58c67154-3f10-4155-9085-dcd6e3689008

**Published:** 2011-11-21

**Authors:** Amidou N'Diaye, Gary K. Chen, Cameron D. Palmer, Bing Ge, Bamidele Tayo, Rasika A. Mathias, Jingzhong Ding, Michael A. Nalls, Adebowale Adeyemo, Véronique Adoue, Christine B. Ambrosone, Larry Atwood, Elisa V. Bandera, Lewis C. Becker, Sonja I. Berndt, Leslie Bernstein, William J. Blot, Eric Boerwinkle, Angela Britton, Graham Casey, Stephen J. Chanock, Ellen Demerath, Sandra L. Deming, W. Ryan Diver, Caroline Fox, Tamara B. Harris, Dena G. Hernandez, Jennifer J. Hu, Sue A. Ingles, Esther M. John, Craig Johnson, Brendan Keating, Rick A. Kittles, Laurence N. Kolonel, Stephen B. Kritchevsky, Loic Le Marchand, Kurt Lohman, Jiankang Liu, Robert C. Millikan, Adam Murphy, Solomon Musani, Christine Neslund-Dudas, Kari E. North, Sarah Nyante, Adesola Ogunniyi, Elaine A. Ostrander, George Papanicolaou, Sanjay Patel, Curtis A. Pettaway, Michael F. Press, Susan Redline, Jorge L. Rodriguez-Gil, Charles Rotimi, Benjamin A. Rybicki, Babatunde Salako, Pamela J. Schreiner, Lisa B. Signorello, Andrew B. Singleton, Janet L. Stanford, Alex H. Stram, Daniel O. Stram, Sara S. Strom, Bhoom Suktitipat, Michael J. Thun, John S. Witte, Lisa R. Yanek, Regina G. Ziegler, Wei Zheng, Xiaofeng Zhu, Joseph M. Zmuda, Alan B. Zonderman, Michele K. Evans, Yongmei Liu, Diane M. Becker, Richard S. Cooper, Tomi Pastinen, Brian E. Henderson, Joel N. Hirschhorn, Guillaume Lettre, Christopher A. Haiman

Table S4 was omitted from the published article. Table S4 can be viewed here:

.

