# Supplementary figures and images for: Identification, Replication, and Fine-Mapping of Loci Associated with Adult Height in Individuals of African Ancestry
Source: PLoS Genet. 2011 Oct 6;7(10):e1002298. doi: 10.1371/journal.pgen.1002298 (PMC3188544; doi:10.1371/journal.pgen.1002298)

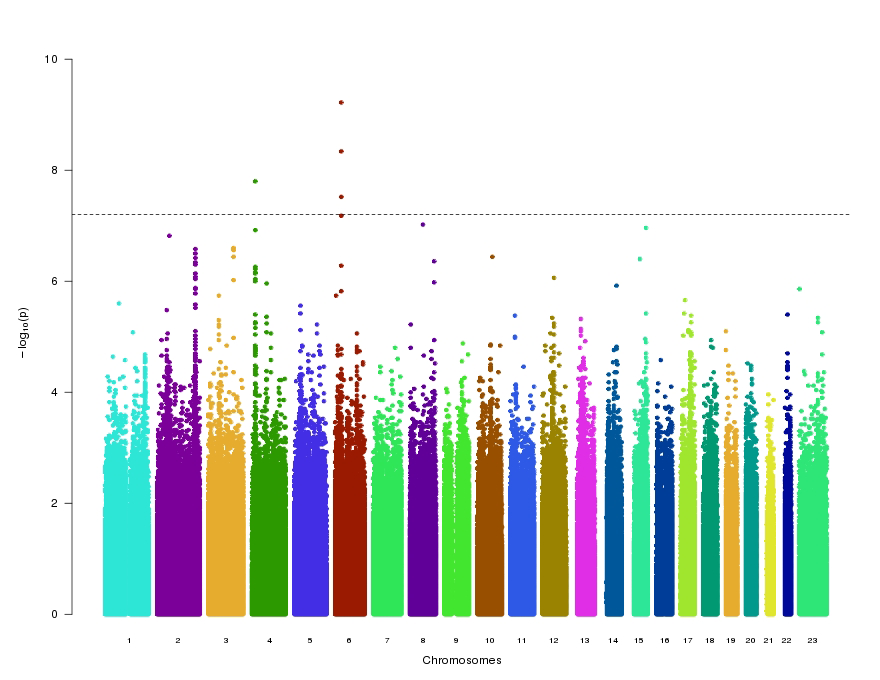

Supplement: Figure S1 — Manhattan plot of the height meta-analysis (3,310,998 SNPs in up to 20,809 participants from 9 studies). The dashed line highlights the genome-wide significance threshold used in this study (P<5×10^−8^). In the discovery phase of the project, SNPs at 4 loci reached genome-wide significance: LCORL on chromosome 4, PPARD on chromosome 6, SULF1 on chromosome 8, and ACAN on chromosome 15. The association between height and SNPs near SULF1 did not replicate. The 3 remaining loci – LCORL, PPARD, and ACAN – are loci previously associated with height in Europeans. Genomic-control P-values are displayed. (TIF) [file pgen.1002298.s001.tif]

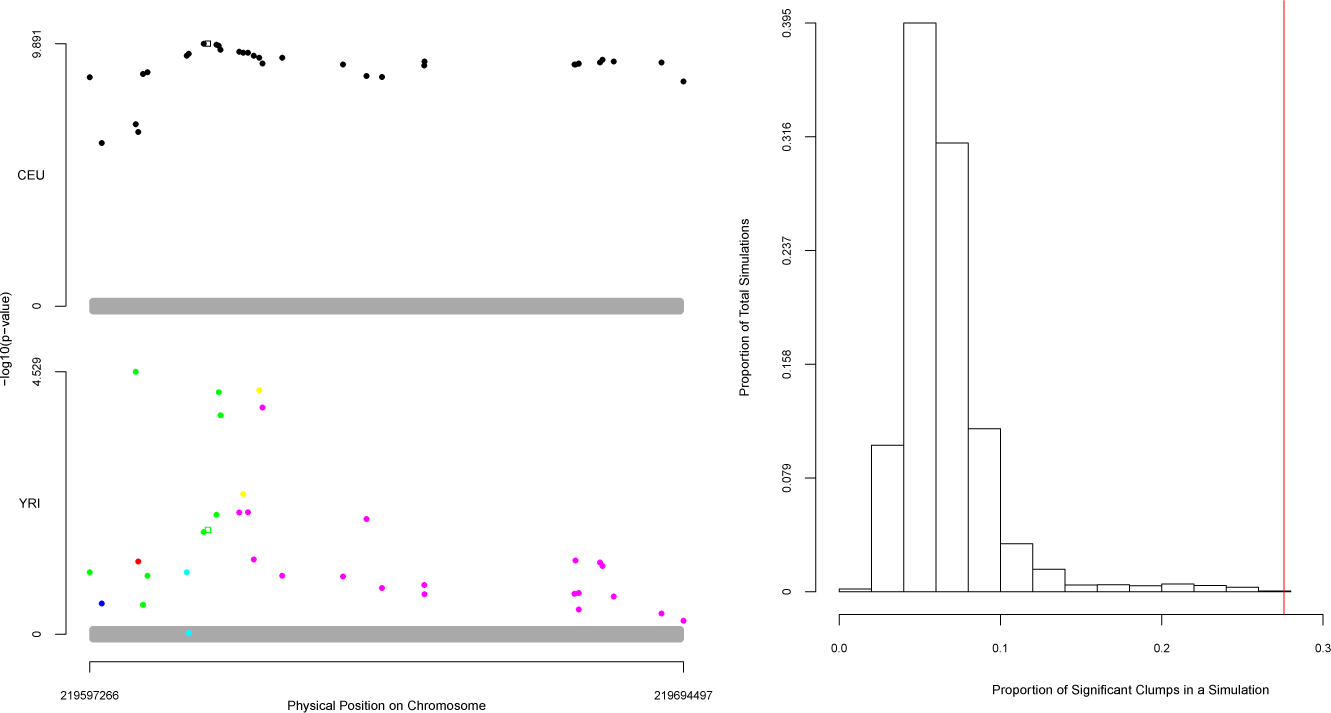

Supplement: Figure S2 — On the left, an example analysis for the European SNP rs12470505 (CCDC108). Top: rs12470505 (square) and proxies (circles; r 2≥0.8 in HapMap2+3 CEU), plotted with their P-values in the GIANT European analysis. Bottom: the same SNPs, plotted with African-American meta-analysis P-values, converted to one-tailed P-values based on predicted direction of effect from the European result and phased HapMap2 CEU data. Colors segregate SNPs into 6 randomly seeded “independent” clusters (r 2≥0.3) using HapMap2+3 YRI linkage disequilibrium estimates. Right: simulation results for the fine-mapping analysis. Simulations were matched to the European SNP list by minor allele frequency; SNPs in each simulation were independent of each other at r 2≥0.2 in HapMap2+3 CEU. The result for each simulation is significant bins/total bins. Red line indicates observed proportion of significant bins for true European SNP replication (P = 8.6×10^−6^). (TIF) [file pgen.1002298.s002.tif]
