# Supplementary material for: Identification, Replication, and Fine-Mapping of Loci Associated with Adult Height in Individuals of African Ancestry
Source: PLoS Genet. 2011 Oct 6;7(10):e1002298. doi: 10.1371/journal.pgen.1002298 (PMC3188544; doi:10.1371/journal.pgen.1002298)
Supplement: Table S1 — Baseline characteristics of cohorts involved in the study. (DOC) [file pgen.1002298.s003.doc]

**Table S1.** Baseline characteristics of cohorts involved in the study

|  |  | **Men** | | | | | | **Women** | | | | | | **Global African Ancestry (mean ± SD)** |
| --- | --- | --- | --- | --- | --- | --- | --- | --- | --- | --- | --- | --- | --- | --- |
| **cohorts** | **trait** | **sample size** | **mean** | **SD** | **median** | **min** | **max** | **sample size** | **mean** | **SD** | **median** | **min** | **max** |  |
| AABC(Women only) | Height(cm) | NA | | | | | | 5380 | 163.4 | 5.9 | 163.0 | 149.9 | 177.5 | 78% ± 14% |
|  | Age(yrs) | NA | | | | | | 5380 | 56.6 | 12.6 | 56.0 | 22.0 | 87.0 |  |
|  |  |  |  |  |  |  |  |  |  |  |  |  |  |  |
| AAPC(Men only) | Height(cm) | 5526 | 178.1 | 6.6 | 177.8 | 163.8 | 194.3 | NA | | | | | | 79% ± 14% |
|  | Age(yrs) | 5526 | 64.6 | 9.1 | 65.0 | 31.3 | 95.0 | NA | | | | | |  |
|  |  |  |  |  |  |  |  |  |  |  |  |  |  |  |
| ARIC | Height(cm) | 1015 | 176.1 | 6.6 | 176.0 | 156.0 | 197.0 | 1725 | 163.1 | 6.1 | 163.0 | 143.0 | 188.0 | 82% ± 11% |
|  | Age(yrs) | 1015 | 53.5 | 6.0 | 53.0 | 44.0 | 66.0 | 1725 | 53.2 | 5.7 | 53.0 | 44.0 | 65.0 |  |
|  |  |  |  |  |  |  |  |  |  |  |  |  |  |  |
| CARDIA | Height(cm) | 248 | 177.6 | 6.8 | 178.0 | 156.0 | 195.0 | 451 | 163.8 | 7.0 | 163.5 | 143.0 | 186.0 | 81% ± 11% |
|  | Age(yrs) | 248 | 26.5 | 2.5 | 26.0 | 23.0 | 34.0 | 451 | 26.0 | 2.9 | 26.0 | 21.0 | 34.0 |  |
|  |  |  |  |  |  |  |  |  |  |  |  |  |  |  |
| CFS | Height(cm) | 151 | 177.9 | 7.0 | 177.8 | 154.9 | 203.2 | 235 | 163.3 | 6.2 | 163.8 | 144.8 | 176.5 | 79% ± 12% |
|  | Age(yrs) | 151 | 48.0 | 13.4 | 46.8 | 24.5 | 80.8 | 235 | 47.6 | 14.9 | 46.3 | 21.0 | 79.7 |  |
|  |  |  |  |  |  |  |  |  |  |  |  |  |  |  |
| JHS | Height(cm) | 827 | 178.1 | 6.8 | 178.0 | 158.0 | 200.0 | 1292 | 164.4 | 6.5 | 164.0 | 142.0 | 188.0 | 82% ± 9% |
|  | Age(yrs) | 827 | 49.6 | 11.6 | 49.0 | 23.0 | 84.0 | 1292 | 50.2 | 12.0 | 49.0 | 21.0 | 85.0 |  |
|  |  |  |  |  |  |  |  |  |  |  |  |  |  |  |
| MAYWOOD | Height(cm) | 465 | 176.1 | 6.9 | 176.5 | 151.5 | 194.0 | 278 | 164.2 | 6.8 | 164.0 | 149.5 | 193.5 | 80% ± 8% |
|  | Age(yrs) | 465 | 43.1 | 7.6 | 43.7 | 20.0 | 70.0 | 278 | 40.9 | 7.8 | 41.1 | 24.0 | 71.0 |  |
|  |  |  |  |  |  |  |  |  |  |  |  |  |  |  |
| MESA | Height(cm) | 745 | 175.8 | 6.8 | 176.0 | 152.5 | 196.7 | 901 | 162.2 | 6.7 | 162.0 | 136.9 | 188.4 | 79% ± 14% |
|  | Age(yrs) | 745 | 62.5 | 10.3 | 63.0 | 45.0 | 84.0 | 901 | 62.0 | 10.0 | 62.0 | 45.0 | 84.0 |  |
|  |  |  |  |  |  |  |  |  |  |  |  |  |  |  |
| NIGERIA | Height(cm) | 511 | 170.1 | 7.7 | 170.3 | 102.0 | 192.5 | 677 | 159.6 | 7.0 | 159.6 | 130.8 | 183.5 | ~100% |
|  | Age(yrs) | 511 | 46.7 | 16.3 | 44.0 | 16.0 | 89.0 | 677 | 48.6 | 14.8 | 48.0 | 19.0 | 95.0 |  |
|  |  |  |  |  |  |  |  |  |  |  |  |  |  |  |
| HANDLS | Height(cm) | 441 | 176.7 | 6.8 | 177.0 | 158.0 | 196.0 | 552 | 164.3 | 6.7 | 165.0 | 146.0 | 187.0 | Replication |
|  | Age(yrs) | 441 | 48.7 | 8.7 | 49.0 | 30.0 | 64.0 | 552 | 48.4 | 9.2 | 49.0 | 30.0 | 64.0 |  |
|  |  |  |  |  |  |  |  |  |  |  |  |  |  |  |
| HABC | Height(cm) | 488 | 173.0 | 7.0 | 172.9 | 156.6 | 200.7 | 651 | 159.6 | 6.5 | 159.3 | 137.0 | 181.0 | Replication |
|  | Age(yrs) | 488 | 73.5 | 2.8 | 73.0 | 69.0 | 80.0 | 651 | 73.4 | 3.0 | 73.0 | 68.0 | 80.0 |  |
|  |  |  |  |  |  |  |  |  |  |  |  |  |  |  |
| GeneSTAR | Height(cm) | 439 | 177.6 | 7.4 | 177.8 | 137.7 | 200.7 | 709 | 164.4 | 6.9 | 164.1 | 139.7 | 188.5 | Replication |
|  | Age(yrs) | 439 | 42.8 | 10.7 | 43.0 | 20.0 | 71.0 | 709 | 43.2 | 10.6 | 44.0 | 20.0 | 75.0 |  |
|  |  |  |  |  |  |  |  |  |  |  |  |  |  |  |
| WHI(Women only) | Height(cm) | NA | | | | | | 8149 | 162.5 | 6.2 | 162.5 | 146.2 | 177.0 | Replication |
|  | Age(yrs) | NA | | | | | | 8149 | 61.6 | 7.0 | 61.0 | 50 | 79 |  |
|  |  |  |  |  |  |  |  |  |  |  |  |  |  |  |
| AABC+AABC+AAT2D (MEC) | Height(cm) | 2525 | 178.0 | 6.4 | 177.8 | 165.1 | 193.0 | 2578 | 163.3 | 5.8 | 162.6 | 149.9 | 175.3 | Replication |
|  | Age(yrs) | 2525 | 68.1 | 8.0 | 69.0 | 45.0 | 88.0 | 2578 | 63.2 | 10.0 | 63.0 | 45.0 | 87.0 |  |
|  |  |  |  |  |  |  |  |  |  |  |  |  |  |  |
| Latino BC + PC (MEC) | Height(cm) | 1955 | 171.6 | 5.7 | 170.2 | 160.0 | 182.9 | 1024 | 158.9 | 5.2 | 157.5 | 147.3 | 170.2 | Replication |
|  | Age(yrs) | 1955 | 62.6 | 6.6 | 63.0 | 45.0 | 76.0 | 1024 | 65.3 | 7.8 | 65.0 | 46.0 | 90.0 |  |
|  |  |  |  |  |  |  |  |  |  |  |  |  |  |  |
| Japanese BC + PC (MEC) | Height(cm) | 1858 | 167.3 | 5.0 | 167.6 | 157.5 | 177.8 | 1629 | 155.4 | 4.7 | 154.9 | 144.8 | 165.1 | Replication |
|  | Age(yrs) | 1858 | 63.9 | 7.4 | 65.0 | 45.0 | 76.0 | 1629 | 66.5 | 8.5 | 67.0 | 46.0 | 88.0 |  |
