# Supplementary material for: Identification, Replication, and Fine-Mapping of Loci Associated with Adult Height in Individuals of African Ancestry
Source: PLoS Genet. 2011 Oct 6;7(10):e1002298. doi: 10.1371/journal.pgen.1002298 (PMC3188544; doi:10.1371/journal.pgen.1002298)
Supplement: Table S2 — Association results for the top 153 SNPs in the discovery meta-analysis. Positions are on NCBI build 36.1 (hg18) and the alleles are on the forward strand. Beta (effect size) and SE (standard error) are in standardized ‘Z-score’ units. (DOC) [file pgen.1002298.s004.doc]

**Table S2**. Association results for the top 153 SNPs in the discovery meta-analysis. Positions are on NCBI build 36.1 (hg18) and the alleles are on the forward strand. Beta (effect size) and SE (standard error) are in standardized ‘Z-score’ units.

|  |  | Discovery | | | | | | | Replication | | Combined | |
| --- | --- | --- | --- | --- | --- | --- | --- | --- | --- | --- | --- | --- |
| SNP | CHR(POS) | Locus | Effect allele/other allele | Effect allele frequency | Beta(SE) | GC-corrected  P-value | Hetero.  P-value | I^2^ (%) | Beta(SE) | P-value | Beta(SE) | P-value |
| rs11265313 | 1(158296080) | KCNJ10 | A/G | 0.34 | 0.048(0.01) | 8.4x10^-6^ | 0.74 | 0 | 0.018(0.015) | 2.4x10^-1^ | 0.038(0.009) | 1.5x10^-5^ |
| rs2182766 | 1(81569056) |  | A/G | 0.93 | -0.1(0.021) | 2.5x10^-6^ | 0.66 | 0 | 0.027(0.026) | 2.8x10^-1^ | -0.048(0.016) | 3.3x10^-3^ |
| rs3856477 | 2(217796633) |  | A/G | 0.43 | -0.047(0.009) | 1.3x10^-6^ | 0.69 | 0 | 0.03(0.014) | 2.8x10^-2^ | -0.021(0.008) | 7.3x10^-3^ |
| rs2115862 | 2(217803299) |  | C/G | 0.17 | 0.062(0.013) | 3.0x10^-6^ | 0.76 | 0 | 0.022(0.018) | 2.2x10^-1^ | 0.048(0.011) | 7.0x10^-6^ |
| rs2565689 | 2(217818793) |  | A/T | 0.35 | 0.048(0.01) | 1.7x10^-6^ | 0.97 | 0 | 0.015(0.014) | 2.9x10^-1^ | 0.037(0.008) | 6.2x10^-6^ |
| rs2113827 | 2(217819555) |  | T/C | 0.18 | 0.064(0.012) | 5.1x10^-7^ | 0.78 | 0 | 0.038(0.017) | 2.9x10^-2^ | 0.055(0.01) | 9.1x10^-8^ |
| rs2565688 | 2(217819946) |  | A/T | 0.18 | 0.063(0.013) | 1.4x10^-6^ | 0.76 | 0 | 0.034(0.017) | 4.3x10^-2^ | 0.052(0.01) | 3.7x10^-7^ |
| rs2553028 | 2(217826201) |  | C/G | 0.81 | -0.062(0.012) | 9.3x10^-7^ | 0.8 | 0 | -0.029(0.017) | 8.8x10^-2^ | -0.05(0.01) | 7.3x10^-7^ |
| rs2553027 | 2(217829855) |  | T/C | 0.19 | 0.063(0.012) | 4.5x10^-7^ | 0.84 | 0 | 0.032(0.017) | 6.5x10^-2^ | 0.052(0.01) | 2.6x10^-7^ |
| rs2553026 | 2(217831893) |  | A/G | 0.19 | 0.063(0.012) | 7.1x10^-7^ | 0.86 | 0 | 0.042(0.017) | 1.4x10^-2^ | 0.056(0.01) | 5.5x10^-8^ |
| rs1863190 | 2(217836397) |  | A/T | 0.8 | -0.064(0.012) | 3.1x10^-7^ | 0.83 | 0 | -0.031(0.017) | 5.8x10^-2^ | -0.052(0.01) | 1.6x10^-7^ |
| rs7578283 | 2(218225964) |  | T/C | 0.46 | 0.043(0.009) | 1.1x10^-5^ | 0.35 | 0 | 0.025(0.014) | 6.6x10^-2^ | 0.037(0.008) | 3.2x10^-6^ |
| rs6435999 | 2(218231407) |  | A/G | 0.64 | 0.047(0.01) | 2.6x10^-6^ | 0.35 | 0 | 0.027(0.014) | 5.8x10^-2^ | 0.041(0.008) | 7.3x10^-7^ |
| rs13035160 | 2(218232077) |  | T/G | 0.47 | 0.053(0.011) | 8.4x10^-7^ | 0.33 | 0 | -0.041(0.014) | 4.5x10^-3^ | 0.019(0.009) | 2.5x10^-2^ |
| rs1430125 | 2(218244900) |  | A/G | 0.46 | 0.05(0.009) | 2.7x10^-7^ | 0.25 | 1.6 | -0.031(0.014) | 2.3x10^-2^ | 0.023(0.008) | 4.3x10^-3^ |
| rs6761086 | 2(218251675) |  | T/C | 0.5 | -0.049(0.009) | 3.9x10^-7^ | 0.31 | 0 | -0.015(0.014) | 2.8x10^-1^ | -0.038(0.008) | 1.9x10^-6^ |
| rs3791950 | 2(218438110) | TNS1 | A/C | 0.89 | -0.07(0.016) | 1.7x10^-5^ | 0.65 | 0 | -0.046(0.021) | 2.8x10^-2^ | -0.061(0.013) | 2.0x10^-6^ |
| rs1863133 | 2(218440453) | TNS1 | T/C | 0.11 | 0.071(0.016) | 1.5x10^-5^ | 0.65 | 0 | 0.041(0.023) | 7.7x10^-2^ | 0.061(0.013) | 4.9x10^-6^ |
| rs1424916 | 2(218447545) | TNS1 | T/C | 0.1 | 0.074(0.016) | 8.1x10^-6^ | 0.81 | 0 | 0.023(0.023) | 3.2x10^-1^ | 0.056(0.013) | 2.6x10^-5^ |
| rs10197074 | 2(219414779) |  | T/C | 0.1 | 0.097(0.021) | 9.7x10^-6^ | 0.33 | 0 | 0.031(0.05) | 5.4x10^-1^ | 0.086(0.02) | 1.7x10^-5^ |
| rs1512692 | 2(219975571) |  | A/C | 0.58 | 0.043(0.009) | 1.3x10^-5^ | 0.03 | 40.7 | 0.009(0.014) | 5.2x10^-1^ | 0.031(0.008) | 8.1x10^-5^ |
| rs1512693 | 2(219975756) |  | T/C | 0.42 | -0.042(0.009) | 1.6x10^-5^ | 0.04 | 38.8 | -0.007(0.014) | 6.0x10^-1^ | -0.03(0.008) | 1.4x10^-4^ |
| rs12466021 | 2(219977009) |  | A/G | 0.27 | -0.047(0.011) | 1.4x10^-5^ | 0 | 57.6 | -0.003(0.015) | 8.6x10^-1^ | -0.032(0.009) | 2.9x10^-4^ |
| rs1991083 | 2(23740942) | KLHL29 | T/C | 0.39 | 0.044(0.01) | 1.1x10^-5^ | 0.48 | 0 | 0.014(0.014) | 3.3x10^-1^ | 0.034(0.008) | 3.6x10^-5^ |
| rs2589113 | 2(55369827) | CCDC88A | T/G | 0.48 | 0.043(0.009) | 1.1x10^-5^ | 0.35 | 0 | 0.048(0.014) | 4.0x10^-4^ | 0.044(0.008) | 1.7x10^-8^ |
| rs706548 | 2(55703610) |  | C/G | 0.85 | 0.076(0.017) | 1.7x10^-5^ | 0.65 | 0 | 0.008(0.02) | 6.8x10^-1^ | 0.046(0.013) | 4.8x10^-4^ |
| rs11680015 | 2(55943743) |  | A/G | 0.76 | -0.094(0.02) | 3.3x10^-6^ | 0.49 | 0 | 0.016(0.018) | 3.7x10^-1^ | -0.033(0.014) | 1.4x10^-2^ |
| rs7598185 | 2(59805763) |  | A/C | 0.26 | -0.058(0.013) | 8.7x10^-6^ | 0.82 | 0 | -0.024(0.016) | 1.3x10^-1^ | -0.044(0.01) | 1.2x10^-5^ |
| rs4315565 | 2(69141447) | ANTXR1 | A/G | 0.2 | -0.067(0.012) | 1.5x10^-7^ | 0.83 | 0 | -0.044(0.018) | 1.3x10^-2^ | -0.059(0.01) | 1.2x10^-8^ |
| rs5749482 | 22(31389665) | SYN3 | C/G | 0.44 | -0.045(0.01) | 4.0x10^-6^ | 0.78 | 0 | -0.031(0.014) | 2.7x10^-2^ | -0.04(0.008) | 4.7x10^-7^ |
| rs7619048 | 3(101409927) |  | A/G | 0.2 | 0.052(0.012) | 1.5x10^-5^ | 0.96 | 0 | 0.019(0.017) | 2.8x10^-1^ | 0.041(0.01) | 3.0x10^-5^ |
| rs6440003 | 3(142576899) | ZBTB38 | A/G | 0.81 | 0.064(0.012) | 3.7x10^-7^ | 0.48 | 0 | 0.066(0.017) | 1.3x10^-4^ | 0.065(0.01) | 1.9x10^-10^ |
| rs6764769 | 3(142582970) | ZBTB38 | A/G | 0.19 | -0.061(0.012) | 9.5x10^-7^ | 0.53 | 0 | -0.065(0.017) | 1.4x10^-4^ | -0.063(0.01) | 5.4x10^-10^ |
| rs6763931 | 3(142585523) | ZBTB38 | A/G | 0.81 | 0.061(0.012) | 9.5x10^-7^ | 0.52 | 0 | 0.066(0.017) | 1.3x10^-4^ | 0.063(0.01) | 5.2x10^-10^ |
| rs724016 | 3(142588260) | ZBTB38 | A/G | 0.26 | -0.049(0.011) | 1.1x10^-5^ | 0.27 | 0 | -0.051(0.015) | 7.0x10^-4^ | -0.049(0.009) | 2.9x10^-8^ |
| rs2871960 | 3(142604504) | ZBTB38 | A/C | 0.19 | -0.066(0.012) | 2.5x10^-7^ | 0.26 | 0.7 | -0.06(0.017) | 5.8x10^-4^ | -0.064(0.01) | 5.7x10^-10^ |
| rs1344674 | 3(142607876) | ZBTB38 | A/G | 0.19 | -0.065(0.012) | 2.7x10^-7^ | 0.26 | 0.5 | -0.056(0.017) | 1.2x10^-3^ | -0.062(0.01) | 1.4x10^-9^ |
| rs7637449 | 3(56603071) | CCDC66 | A/G | 0.16 | 0.06(0.013) | 5.9x10^-6^ | 0.87 | 0 | 0.035(0.018) | 5.9x10^-2^ | 0.051(0.011) | 1.8x10^-6^ |
| rs9833926 | 3(56625218) | CCDC66 | A/G | 0.16 | 0.06(0.013) | 6.6x10^-6^ | 0.91 | 0 | 0.026(0.018) | 1.5x10^-1^ | 0.048(0.011) | 7.0x10^-6^ |
| rs958755 | 3(56691962) | C3orf63 | T/G | 0.16 | 0.059(0.013) | 9.2x10^-6^ | 0.91 | 0 | 0.026(0.018) | 1.5x10^-1^ | 0.047(0.011) | 9.6x10^-6^ |
| rs6773931 | 3(56710220) |  | A/C | 0.8 | -0.056(0.012) | 4.9x10^-6^ | 0.99 | 0 | -0.048(0.017) | 4.2x10^-3^ | -0.053(0.01) | 7.5x10^-8^ |
| rs6445815 | 3(56715141) |  | A/G | 0.22 | 0.056(0.011) | 1.8x10^-6^ | 0.96 | 0 | 0.034(0.016) | 3.6x10^-2^ | 0.048(0.01) | 3.5x10^-7^ |
| rs6768755 | 3(60261526) | FHIT | T/C | 0.36 | 0.078(0.017) | 1.2x10^-5^ | 0.84 | 0 | 0.008(0.016) | 6.4x10^-1^ | 0.04(0.012) | 9.4x10^-4^ |
| rs11707481 | 3(8929385) | RAD18 | A/G | 0.17 | 0.056(0.013) | 1.7x10^-5^ | 0.37 | 0 | 0.015(0.018) | 4.0x10^-1^ | 0.042(0.011) | 6.8x10^-5^ |
| rs4956020 | 4(108791600) | PAPSS1 | T/C | 0.08 | 0.077(0.017) | 8.9x10^-6^ | 0.43 | 0 | 0.015(0.023) | 5.1x10^-1^ | 0.054(0.014) | 8.4x10^-5^ |
| rs4956119 | 4(108812539) | PAPSS1 | T/C | 0.92 | -0.075(0.017) | 1.6x10^-5^ | 0.57 | 0 | -0.002(0.023) | 9.2x10^-1^ | -0.048(0.014) | 4.6x10^-4^ |
| rs2074974 | 4(17421713) | NCAPG | A/C | 0.31 | 0.056(0.012) | 5.9x10^-6^ | 0.25 | 1.2 | 0.054(0.014) | 1.7x10^-4^ | 0.056(0.009) | 3.6x10^-9^ |
| rs2302211 | 4(17428233) | NCAPG | A/T | 0.09 | -0.074(0.017) | 1.7x10^-5^ | 0.16 | 15.9 | -0.024(0.024) | 3.3x10^-1^ | -0.057(0.014) | 4.8x10^-5^ |
| rs2286538 | 4(17435958) | NCAPG | C/G | 0.91 | 0.072(0.016) | 1.6x10^-5^ | 0.28 | 0 | 0.032(0.022) | 1.5x10^-1^ | 0.057(0.013) | 1.6x10^-5^ |
| rs16895878 | 4(17456379) | LCORL | A/C | 0.91 | 0.072(0.016) | 1.5x10^-5^ | 0.31 | 0 | 0.024(0.024) | 3.2x10^-1^ | 0.056(0.014) | 3.9x10^-5^ |
| rs16895895 | 4(17463866) | LCORL | T/C | 0.91 | 0.071(0.016) | 1.7x10^-5^ | 0.22 | 6.5 | 0.02(0.024) | 4.1x10^-1^ | 0.054(0.014) | 5.9x10^-5^ |
| rs6844379 | 4(17465899) | LCORL | T/C | 0.65 | -0.05(0.01) | 7.2x10^-7^ | 0.42 | 0 | -0.075(0.014) | 6.7x10^-8^ | -0.058(0.008) | 7.0x10^-13^ |
| rs925098 | 4(17528909) | LCORL | A/G | 0.65 | -0.056(0.01) | 1.6x10^-8^ | 0.39 | 0 | -0.081(0.014) | 8.6x10^-9^ | -0.065(0.008) | 2.3x10^-15^ |
| rs7663818 | 4(17545541) | LCORL | T/C | 0.39 | -0.049(0.01) | 5.5x10^-7^ | 0.96 | 0 | -0.054(0.014) | 8.3x10^-5^ | -0.051(0.008) | 2.2x10^-10^ |
| rs7692995 | 4(17545732) | LCORL | T/C | 0.61 | 0.049(0.01) | 6.9x10^-7^ | 0.96 | 0 | 0.044(0.014) | 1.4x10^-3^ | 0.047(0.008) | 4.0x10^-9^ |
| rs2707450 | 4(17551658) | LCORL | T/C | 0.74 | -0.053(0.011) | 9.1x10^-7^ | 0.52 | 0 | -0.063(0.015) | 2.5x10^-5^ | -0.057(0.009) | 1.2x10^-10^ |
| rs6853216 | 4(17579753) | LCORL | T/C | 0.47 | -0.044(0.009) | 4.1x10^-6^ | 0.78 | 0 | -0.044(0.013) | 1.1x10^-3^ | -0.044(0.008) | 1.5x10^-8^ |
| rs2320299 | 4(17581470) | LCORL | A/G | 0.63 | -0.049(0.01) | 6.0x10^-7^ | 0.63 | 0 | -0.061(0.014) | 1.0x10^-5^ | -0.053(0.008) | 3.3x10^-11^ |
| rs16896210 | 4(17601430) | LCORL | A/G | 0.56 | 0.044(0.009) | 5.6x10^-6^ | 0.73 | 0 | 0.054(0.013) | 5.2x10^-5^ | 0.047(0.008) | 1.6x10^-9^ |
| rs724577 | 4(17602508) | LCORL | A/C | 0.31 | 0.054(0.01) | 1.2x10^-7^ | 0.28 | 0 | 0.091(0.014) | 1.9x10^-10^ | 0.067(0.008) | 1.1x10^-15^ |
| rs16896236 | 4(17612910) | LCORL | T/C | 0.57 | 0.055(0.012) | 9.0x10^-6^ | 0.84 | 0 | 0.033(0.029) | 2.5x10^-1^ | 0.051(0.011) | 5.7x10^-6^ |
| rs16896261 | 4(17620300) | LCORL | A/G | 0.65 | 0.049(0.01) | 1.0x10^-6^ | 0.93 | 0 | 0.056(0.014) | 7.1x10^-5^ | 0.051(0.008) | 3.3x10^-10^ |
| rs6830062 | 4(17626828) | LCORL | T/C | 0.56 | 0.043(0.009) | 7.0x10^-6^ | 0.88 | 0 | 0.027(0.014) | 4.4x10^-2^ | 0.038(0.008) | 1.4x10^-6^ |
| rs1380294 | 4(17633219) | LCORL | T/C | 0.44 | -0.044(0.009) | 5.9x10^-6^ | 0.86 | 0 | -0.05(0.013) | 2.0x10^-4^ | -0.046(0.008) | 4.6x10^-9^ |
| rs710839 | 4(82353811) |  | T/C | 0.39 | -0.047(0.01) | 4.4x10^-6^ | 0.72 | 0 | -0.042(0.015) | 5.9x10^-3^ | -0.046(0.009) | 8.8x10^-8^ |
| rs710841 | 4(82368855) |  | T/C | 0.65 | 0.046(0.01) | 5.7x10^-6^ | 0.87 | 0 | 0.044(0.014) | 2.1x10^-3^ | 0.045(0.008) | 4.3x10^-8^ |
| rs1662840 | 4(82375433) |  | T/C | 0.69 | 0.051(0.01) | 1.1x10^-6^ | 0.96 | 0 | 0.046(0.015) | 1.8x10^-3^ | 0.05(0.009) | 7.3x10^-9^ |
| rs994014 | 4(82384814) |  | T/C | 0.29 | -0.052(0.01) | 1.1x10^-6^ | 0.85 | 0 | -0.056(0.015) | 1.7x10^-4^ | -0.054(0.009) | 7.8x10^-10^ |
| rs1961460 | 4(82393522) |  | A/G | 0.73 | 0.05(0.011) | 8.3x10^-6^ | 0.98 | 0 | -0.041(0.015) | 7.1x10^-3^ | 0.018(0.009) | 4.6x10^-2^ |
| rs2042327 | 5(127650882) | FBN2 | T/C | 0.17 | 0.072(0.016) | 8.8x10^-6^ | 0.44 | 0 | 0(0.018) | 9.9x10^-1^ | 0.039(0.012) | 9.7x10^-4^ |
| rs27855 | 5(127668760) | FBN2 | A/G | 0.81 | -0.057(0.012) | 6.1x10^-6^ | 0.05 | 36.1 | -0.028(0.017) | 1.1x10^-1^ | -0.047(0.01) | 3.8x10^-6^ |
| rs7704625 | 5(144044847) |  | A/G | 0.38 | 0.043(0.01) | 1.4x10^-5^ | 0.07 | 30.3 | 0.01(0.014) | 4.7x10^-1^ | 0.032(0.008) | 7.6x10^-5^ |
| rs724382 | 5(144045355) |  | A/G | 0.62 | -0.043(0.01) | 1.8x10^-5^ | 0.09 | 26.7 | -0.009(0.014) | 5.2x10^-1^ | -0.031(0.008) | 1.1x10^-4^ |
| rs4076512 | 5(33058895) |  | T/C | 0.84 | -0.062(0.013) | 2.7x10^-6^ | 0.92 | 0 | -0.003(0.018) | 8.8x10^-1^ | -0.041(0.011) | 1.2x10^-4^ |
| rs35397 | 5(33986873) | SLC45A2 | T/G | 0.23 | 0.058(0.012) | 3.8x10^-6^ | 0.12 | 22.5 | 0.018(0.017) | 2.9x10^-1^ | 0.045(0.01) | 1.4x10^-5^ |
| rs183671 | 5(33999967) | SLC45A2 | T/G | 0.69 | -0.047(0.011) | 1.8x10^-5^ | 0.05 | 35.4 | -0.02(0.016) | 2.2x10^-1^ | -0.038(0.009) | 2.4x10^-5^ |
| rs2937550 | 5(36508994) |  | T/C | 0.2 | 0.056(0.012) | 7.5x10^-6^ | 0.22 | 7.2 | 0.025(0.018) | 1.6x10^-1^ | 0.045(0.01) | 7.6x10^-6^ |
| rs4957397 | 5(41569372) |  | A/G | 0.77 | -0.05(0.011) | 1.4x10^-5^ | 0.72 | 0 | -0.012(0.016) | 4.4x10^-1^ | -0.037(0.009) | 7.4x10^-5^ |
| rs4957398 | 5(41569404) |  | A/T | 0.23 | 0.05(0.011) | 1.5x10^-5^ | 0.75 | 0 | 0.02(0.017) | 2.3x10^-1^ | 0.04(0.01) | 2.2x10^-5^ |
| rs9321065 | 6(126785780) |  | A/G | 0.3 | 0.045(0.01) | 1.5x10^-5^ | 0.77 | 0 | 0.006(0.015) | 6.8x10^-1^ | 0.032(0.009) | 1.6x10^-4^ |
| rs1361108 | 6(126809293) |  | T/C | 0.3 | 0.045(0.01) | 1.8x10^-5^ | 0.69 | 0 | 0.025(0.015) | 9.7x10^-2^ | 0.038(0.009) | 8.7x10^-6^ |
| rs4422634 | 6(126848369) |  | T/C | 0.74 | -0.051(0.011) | 8.8x10^-6^ | 1 | 0 | -0.014(0.016) | 3.8x10^-1^ | -0.039(0.009) | 3.6x10^-5^ |
| rs9495232 | 6(139110942) |  | A/T | 0.88 | -0.065(0.015) | 1.8x10^-5^ | 0.29 | 0 | -0.017(0.022) | 4.5x10^-1^ | -0.05(0.013) | 7.8x10^-5^ |
| rs853356 | 6(14281407) |  | A/G | 0.29 | -0.045(0.01) | 1.7x10^-5^ | 0.96 | 0 | -0.031(0.015) | 4.1x10^-2^ | -0.04(0.009) | 2.7x10^-6^ |
| rs853382 | 6(14287755) |  | A/G | 0.29 | -0.046(0.01) | 1.5x10^-5^ | 0.95 | 0 | -0.019(0.015) | 2.2x10^-1^ | -0.037(0.009) | 2.0x10^-5^ |
| rs1150781 | 6(34322300) | HMGA1 | C/G | 0.42 | 0.044(0.01) | 1.7x10^-5^ | 0.54 | 0 | 0.056(0.015) | 1.8x10^-4^ | 0.048(0.009) | 1.6x10^-8^ |
| rs16874062 | 6(35359173) | ZNF76 | A/G | 0.83 | 0.075(0.013) | 4.7x10^-9^ | 0.23 | 5.4 | 0.112(0.035) | 1.5x10^-3^ | 0.08(0.012) | 4.6x10^-11^ |
| rs16874262 | 6(35373689) | DEF6 | T/C | 0.17 | -0.072(0.013) | 3.0x10^-8^ | 0.19 | 11.3 | -0.113(0.035) | 1.3x10^-3^ | -0.077(0.012) | 3.0x10^-10^ |
| rs9470003 | 6(35445909) | PPARD | C/G | 0.15 | -0.073(0.013) | 6.5x10^-8^ | 0.16 | 14.7 | -0.11(0.038) | 3.3x10^-3^ | -0.077(0.013) | 1.3x10^-9^ |
| rs9470004 | 6(35449828) | PPARD | T/C | 0.17 | -0.078(0.012) | 6.0x10^-10^ | 0.2 | 9.9 | -0.102(0.035) | 3.3x10^-3^ | -0.081(0.012) | 1.0x10^-11^ |
| rs16868709 | 6(35462449) | PPARD | C/G | 0.83 | 0.061(0.012) | 1.5x10^-6^ | 0 | 56.9 | -0.003(0.018) | 8.8x10^-1^ | 0.04(0.01) | 9.9x10^-5^ |
| rs9462078 | 6(35465952) | PPARD | A/G | 0.84 | 0.068(0.013) | 5.3x10^-7^ | 0.13 | 20.3 | 0.078(0.037) | 3.4x10^-2^ | 0.069(0.013) | 4.9x10^-8^ |
| rs2224391 | 6(5205935) | LYRM4 | A/C | 0.23 | 0.066(0.013) | 1.8x10^-6^ | 0.35 | 0 | 0.026(0.016) | 1.1x10^-1^ | 0.049(0.01) | 3.0x10^-6^ |
| rs13246702 | 7(124571841) |  | A/C | 0.99 | -0.346(0.078) | 1.5x10^-5^ | 0.84 | 0 | -0.149(0.133) | 2.6x10^-1^ | -0.293(0.069) | 1.9x10^-5^ |
| rs7007970 | 8(120717110) | ENPP2 | C/G | 0.73 | -0.047(0.011) | 1.8x10^-5^ | 0.92 | 0 | -0.028(0.016) | 7.4x10^-2^ | -0.041(0.009) | 6.0x10^-6^ |
| rs11988811 | 8(135667857) | ZFAT1 | A/G | 0.29 | -0.046(0.01) | 1.1x10^-5^ | 0.3 | 0 | -0.013(0.015) | 3.9x10^-1^ | -0.035(0.009) | 4.1x10^-5^ |
| rs3739422 | 8(135684154) | ZFAT | A/G | 0.73 | 0.052(0.01) | 1.0x10^-6^ | 0.1 | 25.7 | 0.017(0.015) | 2.5x10^-1^ | 0.041(0.009) | 3.5x10^-6^ |
| rs733254 | 8(135707814) | ZFAT1 | A/C | 0.27 | -0.054(0.01) | 4.4x10^-7^ | 0.19 | 11.6 | -0.016(0.015) | 2.8x10^-1^ | -0.041(0.009) | 2.1x10^-6^ |
| rs4875611 | 8(3165756) | CSMD1 | T/G | 0.28 | -0.057(0.013) | 1.6x10^-5^ | 0.19 | 10.2 | 0.016(0.016) | 3.0x10^-1^ | -0.026(0.01) | 9.6x10^-3^ |
| rs7841495 | 8(3240477) | CSMD1 | A/T | 0.31 | -0.048(0.01) | 6.0x10^-6^ | 0.66 | 0 | 0.013(0.015) | 3.7x10^-1^ | -0.027(0.009) | 1.9x10^-3^ |
| rs6982250 | 8(70575004) | SULF1 | T/C | 0.25 | -0.061(0.011) | 9.7x10^-8^ | 1 | 0 | -0.014(0.017) | 4.1x10^-1^ | -0.046(0.01) | 1.3x10^-6^ |
| rs1929842 | 9(106673444) | ABCA1 | C/G | 0.64 | 0.048(0.011) | 1.3x10^-5^ | 0.07 | 30.8 | 0.015(0.018) | 4.2x10^-1^ | 0.039(0.009) | 3.5x10^-5^ |
| rs1380442 | 10(127849645) | ADAM12 | T/G | 0.56 | -0.044(0.01) | 1.5x10^-5^ | 0.8 | 0 | -0.014(0.016) | 3.8x10^-1^ | -0.036(0.009) | 3.5x10^-5^ |
| rs2704470 | 10(71204189) |  | A/G | 0.33 | -0.044(0.01) | 1.4x10^-5^ | 0.17 | 14.3 | 0.012(0.014) | 4.0x10^-1^ | -0.025(0.008) | 2.4x10^-3^ |
| rs2704471 | 10(71205087) |  | T/C | 0.67 | 0.044(0.01) | 1.4x10^-5^ | 0.2 | 9.9 | 0.02(0.015) | 1.7x10^-1^ | 0.037(0.008) | 1.4x10^-5^ |
| rs941873 | 10(80809468) |  | A/G | 0.41 | -0.054(0.01) | 3.6x10^-7^ | 0.28 | 0 | -0.024(0.015) | 1.2x10^-1^ | -0.044(0.009) | 4.1x10^-7^ |
| rs1596299 | 11(28538133) |  | T/C | 0.49 | 0.042(0.009) | 9.9x10^-6^ | 0.18 | 12.7 | 0.008(0.013) | 5.5x10^-1^ | 0.031(0.008) | 8.3x10^-5^ |
| rs2585824 | 11(28538377) |  | T/G | 0.49 | 0.042(0.009) | 1.1x10^-5^ | 0.25 | 2.3 | 0.003(0.013) | 8.1x10^-1^ | 0.029(0.008) | 1.8x10^-4^ |
| rs2585823 | 11(28538638) |  | A/G | 0.49 | 0.044(0.009) | 4.1x10^-6^ | 0.32 | 0 | 0.008(0.014) | 5.8x10^-1^ | 0.032(0.008) | 4.3x10^-5^ |
| rs1805495 | 12(13601277) |  | T/G | 0.98 | -0.209(0.047) | 1.8x10^-5^ | 0.51 | 0 | -0.082(0.044) | 6.5x10^-2^ | -0.139(0.033) | 2.2x10^-5^ |
| rs764525 | 12(13603254) |  | T/C | 0.99 | -0.213(0.048) | 1.4x10^-5^ | 0.47 | 0 | -0.086(0.05) | 8.4x10^-2^ | -0.151(0.035) | 1.7x10^-5^ |
| rs11836863 | 12(44910279) | SLC38A1 | A/G | 0.51 | 0.043(0.009) | 8.9x10^-6^ | 0.08 | 28.1 | 0.011(0.014) | 4.2x10^-1^ | 0.032(0.008) | 4.1x10^-5^ |
| rs12321906 | 12(52202982) | ATF7 | T/C | 0.87 | 0.061(0.014) | 1.6x10^-5^ | 0.41 | 0 | 0.035(0.02) | 7.6x10^-2^ | 0.052(0.011) | 5.4x10^-6^ |
| rs7304627 | 12(56689427) |  | A/C | 0.48 | 0.071(0.015) | 4.5x10^-6^ | 0.82 | 0 | 0.002(0.014) | 9.1x10^-1^ | 0.031(0.01) | 2.0x10^-3^ |
| rs7979673 | 12(64513524) | HMGA2 | T/C | 0.32 | -0.056(0.012) | 6.7x10^-6^ | 0.6 | 0 | -0.062(0.015) | 2.3x10^-5^ | -0.058(0.01) | 7.3x10^-10^ |
| rs12298541 | 12(64592708) | HMGA2 | A/C | 0.23 | 0.055(0.012) | 5.9x10^-6^ | 0.55 | 0 | 0.073(0.019) | 9.5x10^-5^ | 0.061(0.01) | 3.2x10^-9^ |
| rs1979440 | 12(64632891) | HMGA2 | T/C | 0.6 | 0.048(0.009) | 8.6x10^-7^ | 0.95 | 0 | 0.031(0.014) | 2.6x10^-2^ | 0.042(0.008) | 1.1x10^-7^ |
| rs867633 | 12(64641178) | HMGA2 | A/G | 0.35 | -0.043(0.01) | 1.5x10^-5^ | 0.87 | 0 | -0.048(0.014) | 8.7x10^-4^ | -0.045(0.008) | 4.9x10^-8^ |
| rs7968682 | 12(64658147) |  | T/G | 0.58 | -0.043(0.009) | 9.0x10^-6^ | 0.83 | 0 | 0.033(0.014) | 2.0x10^-2^ | -0.019(0.008) | 1.9x10^-2^ |
| rs9508647 | 13(29606574) |  | T/C | 0.16 | -0.154(0.035) | 1.6x10^-5^ | 0.48 | 0 | 0.001(0.021) | 9.8x10^-1^ | -0.038(0.018) | 3.3x10^-2^ |
| rs9315372 | 13(35369289) | DCLK1 | A/G | 0.57 | -0.044(0.01) | 9.4x10^-6^ | 0.38 | 0 | -0.01(0.014) | 4.8x10^-1^ | -0.033(0.008) | 5.8x10^-5^ |
| rs6563210 | 13(35374447) | DCLK1 | A/G | 0.56 | -0.044(0.009) | 4.7x10^-6^ | 0.4 | 0 | -0.029(0.014) | 3.5x10^-2^ | -0.039(0.008) | 7.3x10^-7^ |
| rs1539542 | 13(35382253) | DCLK1 | A/T | 0.51 | -0.043(0.009) | 7.8x10^-6^ | 0.36 | 0 | -0.017(0.014) | 2.2x10^-1^ | -0.034(0.008) | 1.3x10^-5^ |
| rs2182487 | 13(35382951) | DCLK1 | C/G | 0.51 | -0.043(0.009) | 7.1x10^-6^ | 0.37 | 0 | -0.022(0.014) | 1.0x10^-1^ | -0.036(0.008) | 4.1x10^-6^ |
| rs831223 | 13(59014105) |  | T/C | 0.71 | 0.048(0.011) | 1.2x10^-5^ | 0.68 | 0 | 0.02(0.015) | 1.7x10^-1^ | 0.038(0.009) | 1.4x10^-5^ |
| rs9646158 | 14(58746722) | DAAM1 | T/C | 0.05 | -0.102(0.023) | 1.7x10^-5^ | 0.12 | 21.7 | 0.022(0.028) | 4.4x10^-1^ | -0.051(0.018) | 5.2x10^-3^ |
| rs724743 | 14(75199344) | TTLL5 | A/G | 0.94 | 0.1(0.02) | 1.2x10^-6^ | 0.65 | 0 | 0.109(0.051) | 3.3x10^-2^ | 0.101(0.019) | 1.1x10^-7^ |
| rs4252340 | 14(75500746) | TGFB3 | T/C | 0.71 | 0.046(0.01) | 1.6x10^-5^ | 0.89 | 0 | 0.02(0.015) | 2.0x10^-1^ | 0.037(0.009) | 1.9x10^-5^ |
| rs3917194 | 14(75501286) | TGFB3 | A/T | 0.3 | -0.045(0.01) | 1.5x10^-5^ | 0.84 | 0 | -0.019(0.016) | 2.4x10^-1^ | -0.037(0.009) | 2.2x10^-5^ |
| rs11853893 | 15(52502842) | UNC13C | T/G | 0.97 | -0.157(0.03) | 3.9x10^-7^ | 0.3 | 0 | -0.02(0.041) | 6.2x10^-1^ | -0.107(0.025) | 1.4x10^-5^ |
| rs1383484 | 15(82313759) | ADAMTSL3 | T/C | 0.4 | 0.043(0.01) | 1.1x10^-5^ | 0.97 | 0 | 0.032(0.014) | 2.4x10^-2^ | 0.04(0.008) | 9.3x10^-7^ |
| rs4932199 | 15(87142054) |  | A/G | 0.73 | -0.061(0.013) | 3.8x10^-6^ | 0.08 | 29.6 | -0.044(0.017) | 1.1x10^-2^ | -0.055(0.011) | 1.8x10^-7^ |
| rs2351491 | 15(87199109) | ACAN | T/C | 0.25 | 0.062(0.011) | 1.1x10^-7^ | 0.42 | 0 | 0.048(0.017) | 3.4x10^-3^ | 0.057(0.01) | 1.5x10^-9^ |
| rs420017 | 15(87418085) |  | T/C | 0.21 | 0.052(0.012) | 1.3x10^-5^ | 0.25 | 1.6 | 0.026(0.016) | 1.1x10^-1^ | 0.043(0.01) | 7.3x10^-6^ |
| rs7212932 | 17(12654475) | RICH2 | C/G | 0.61 | 0.044(0.01) | 9.5x10^-6^ | 0.34 | 0 | -0.001(0.015) | 9.4x10^-1^ | 0.031(0.008) | 2.6x10^-4^ |
| rs2428588 | 17(19318151) |  | A/G | 0.37 | 0.083(0.017) | 3.8x10^-6^ | 0.5 | 0 | 0.014(0.016) | 3.9x10^-1^ | 0.045(0.012) | 1.7x10^-4^ |
| rs7213608 | 17(21219882) | KCNJ12 | T/C | 0.81 | 0.06(0.012) | 2.2x10^-6^ | 0.28 | 0 | 0(0.018) | 9.8x10^-1^ | 0.04(0.01) | 1.1x10^-4^ |
| rs9904503 | 17(43239371) | OSBPL7 | T/C | 0.57 | -0.044(0.01) | 8.4x10^-6^ | 0.89 | 0 | -0.014(0.014) | 3.2x10^-1^ | -0.034(0.008) | 2.4x10^-5^ |
| rs9896712 | 17(43239542) | OSBPL7 | A/G | 0.43 | 0.044(0.01) | 7.5x10^-6^ | 0.92 | 0 | 0.011(0.014) | 4.4x10^-1^ | 0.033(0.008) | 4.1x10^-5^ |
| rs9903897 | 17(59070437) | MAP3K3 | A/T | 0.7 | 0.047(0.01) | 9.5x10^-6^ | 0.51 | 0 | 0.06(0.015) | 4.8x10^-5^ | 0.051(0.009) | 2.5x10^-9^ |
| rs11658329 | 17(59116763) | MAP3K3 | C/G | 0.7 | 0.049(0.01) | 4.2x10^-6^ | 0.36 | 0 | 0.063(0.015) | 2.5x10^-5^ | 0.053(0.009) | 5.7x10^-10^ |
| rs15637 | 17(59125782) | MAP3K3 | C/G | 0.3 | -0.046(0.01) | 9.2x10^-6^ | 0.47 | 0 | -0.065(0.015) | 1.5x10^-5^ | -0.053(0.009) | 1.1x10^-9^ |
| rs2854160 | 17(59330980) |  | T/C | 0.21 | -0.053(0.012) | 8.3x10^-6^ | 0.9 | 0 | -0.053(0.017) | 1.7x10^-3^ | -0.053(0.01) | 5.2x10^-8^ |
| rs2854152 | 17(59339759) |  | A/G | 0.2 | -0.057(0.012) | 5.4x10^-6^ | 0.75 | 0 | -0.042(0.018) | 2.0x10^-2^ | -0.052(0.01) | 4.2x10^-7^ |
| rs357897 | 18(44838182) | DYM | T/C | 0.36 | 0.044(0.01) | 1.1x10^-5^ | 0.87 | 0 | 0.058(0.014) | 3.8x10^-5^ | 0.049(0.008) | 2.3x10^-9^ |
| rs1787200 | 18(44841652) | DYM | A/G | 0.37 | 0.044(0.01) | 1.5x10^-5^ | 0.92 | 0 | 0.07(0.014) | 5.5x10^-7^ | 0.053(0.008) | 1.2x10^-10^ |
| rs6566701 | 18(52216786) |  | A/G | 0.06 | -0.087(0.019) | 1.6x10^-5^ | 0.28 | 0 | 0.04(0.026) | 1.3x10^-1^ | -0.04(0.016) | 1.2x10^-2^ |
| rs12985777 | 19(2219055) |  | T/C | 0.68 | 0.047(0.01) | 8.1x10^-6^ | 0.96 | 0 | 0.007(0.015) | 6.4x10^-1^ | 0.033(0.009) | 1.1x10^-4^ |
| rs2523178 | 19(2226079) | C19orf35 | A/G | 0.67 | 0.056(0.013) | 1.7x10^-5^ | 0.72 | 0 | 0.043(0.035) | 2.2x10^-1^ | 0.055(0.012) | 8.1x10^-6^ |
| rs1503198 | X(105000198) | NRK | T/C | 0.41 | -0.041(0.009) | 4.5x10^-6^ | 0.16 | 12.7 | 0.031(0.015) | 4.6x10^-2^ | -0.023(0.008) | 3.4x10^-3^ |
| rs7056631 | X(105001492) | NRK | T/G | 0.4 | -0.041(0.009) | 5.6x10^-6^ | 0.16 | 13.4 | 0.034(0.015) | 2.8x10^-2^ | -0.022(0.008) | 4.2x10^-3^ |
| rs2072837 | X(128518513) | OCRL | A/G | 0.55 | -0.046(0.01) | 8.1x10^-6^ | 0.45 | 0 | -0.017(0.015) | 2.5x10^-1^ | -0.037(0.009) | 1.6x10^-5^ |
| rs12393627 | X(2895723) | ARSE | A/G | 0.63 | -0.093(0.019) | 1.4x10^-6^ | 0.24 | 0 | -0.064(0.012) | 2.6x10^-7^ | -0.072(0.010) | 5.7x10^-12^ |
