# Supplementary material for: Identification, Replication, and Fine-Mapping of Loci Associated with Adult Height in Individuals of African Ancestry
Source: PLoS Genet. 2011 Oct 6;7(10):e1002298. doi: 10.1371/journal.pgen.1002298 (PMC3188544; doi:10.1371/journal.pgen.1002298)
Supplement: Table S3 — Fine-mapping results for SNPs associated with height in Caucasians [49]. We could not fine-map 19 of the 180 SNPs reported by the GIANT Consortium because they were not available in the HapMap phased datasets. In the left-handed side of the table, we present the association results in the African-American height meta-analysis for SNPs associated with height in Caucasians. In the right-handed side of the table, we present results from our fine-mapping experiment using data from our African-American height meta-analysis. For intergenic SNPs, we provide the closest gene and the physical distance between them. (DOC) [file pgen.1002298.s005.doc]

**Table S3**. Fine-mapping results for SNPs associated with height in Caucasians [[49]](#_ENREF_49). We could not fine-map 19 of the 180 SNPs reported by the GIANT Consortium because they were not available in the HapMap phased datasets. In the left-handed side of the table, we present the association results in the African-American height meta-analysis for SNPs associated with height in Caucasians. In the right-handed side of the table, we present results from our fine-mapping experiment using data from our African-American height meta-analysis. For intergenic SNPs, we provide the closest gene and the physical distance between them.

| **Association results in the African-American height meta-analysis for the SNPs associated with height in Caucasians** | | | | | | **Fine-mapping results in the African-American height meta-analysis of the height association signals in Caucasians** | | | | | | |
| --- | --- | --- | --- | --- | --- | --- | --- | --- | --- | --- | --- | --- |
| **European SNP** | **Chr (BP)** | **Effet allele/**  **Other allele** | **Beta (SE)** | **GC-corrected P** | **Annotation** | **Best AfAm SNP** | **Chr (BP)** | **Effet allele/**  **Other allele** | **Beta (SE)** | | **GC-corrected P** | **Annotation** |
| rs425277 | 1 (2059032) | T/C | -0.0014 (0.0153) | 0.927187195 | PRKCZ (intron) | rs17372341 | 1 (2032210) | A/G | 0.0122 (0.0153) | | 0.227663284 | PRKCZ (intron) |
| rs2284746 | 1 (17179262) | C/G | -0.0379 (0.0121) | 0.002309675 | MFAP2 (intron) | rs6657613 | 1 (17200787) | A/T | -0.045 (0.012) | | 0.999915657 | ATP13A2 (intron) |
| rs1738475 | 1 (23409478) | C/G | 0.0242 (0.0096) | 0.014686936 | HTR1D (intergenic, 15669bp) | rs627304 | 1 (23410142) | C/T | 0.026 (0.0097) | | 0.004707634 | HTR1D (intergenic, 16333bp) |
| rs4601530 | 1 (24916698) | T/C | -0.0058 (0.0094) | 0.550388322 | CLIC4 (intergenic, 27648bp) | rs16829945 | 1 (24912902) | C/T | -0.011 (0.0098) | | 0.142194557 | CLIC4 (intergenic, 31444bp) |
| rs7532866 | 1 (26614131) | A/G | -0.0078 (0.01) | 0.449255544 | LIN28A (intron) | rs7532866 | 1 (26614131) | A/G | -0.0078 (0.01) | | 0.788934634 | LIN28A (intron) |
| rs2154319 | 1 (41518357) | T/C | 0.0005 (0.0208) | 0.979835128 | SCMH1 (intergenic, 37955bp) | rs578348 | 1 (41343904) | A/T | 0.0112 (0.0162) | | 0.258247643 | SCMH1 (intron) |
| rs17391694 | 1 (78396214) | T/C | 0.039 (0.0311) | 0.224462903 | GIPC2 (intergenic, 20514bp) | rs17391694 | 1 (78396214) | C/T | 0.039 (0.0311) | | 0.116154841 | GIPC2 (intergenic, 20514bp) |
| rs6699417 | 1 (88896031) | T/C | 0.0086 (0.0095) | 0.378139361 | PKN2 (intergenic, 26478bp) | rs4457557 | 1 (88910047) | A/G | -0.0126 (0.0094) | | 0.100730844 | PKN2 (intergenic, 12462bp) |
| rs10874746 | 1 (93096559) | T/C | -0.014 (0.0095) | 0.151061656 | FAM69A (intron) | rs17380789 | 1 (93160107) | C/T | -0.0194 (0.0104) | | 0.036220854 | FAM69A (intron) |
| rs9428104 | 1 (118657110) | A/G | -0.0187 (0.0102) | 0.075905828 | SPAG17 (intergenic, 127739bp) | rs7536458 | 1 (118666125) | G/T | 0.0465 (0.0121) | | 9.55703E-05 | SPAG17 (intergenic, 136754bp) |
| rs11205277 | 1 (148159496) | A/G | -0.0185 (0.0173) | 0.301674249 | SF3B4 (intergenic, 2338bp) | rs11205277 | 1 (148159496) | A/G | -0.0185 (0.0173) | | 0.155976734 | SF3B4 (intergenic, 2338bp) |
| rs17346452 | 1 (170319910) | T/C | -0.0432 (0.0256) | 0.10203021 | DNM3 (intron) | rs17346473 | 1 (170349716) | A/G | -0.0548 (0.0157) | | 0.000384779 | DNM3 (intron) |
| rs1325598 | 1 (175058872) | A/G | -0.0054 (0.0109) | 0.630236926 | PAPPA2 (intron) | rs10798483 | 1 (175065766) | A/G | 0.0107 (0.0135) | | 0.2276125 | PAPPA2 (intron) |
| rs1046934 | 1 (182290152) | A/C | -0.0345 (0.0131) | 0.010374261 | TSEN15 (missense) | rs1926872 | 1 (182285098) | A/G | -0.0346 (0.0131) | | 0.005288367 | TSEN15 (intergenic, 2335bp) |
| rs10863936 | 1 (210304421) | A/G | 0.0028 (0.01) | 0.782278099 | DTL (intron) | rs10863936 | 1 (210304421) | A/G | 0.0028 (0.01) | | 0.623106092 | DTL (intron) |
| rs11118346 | 1 (217810342) | T/C | 0.0052 (0.012) | 0.67541501 | SLC30A10 (intergenic, 343886bp) | rs11118347 | 1 (217810872) | G/T | 0.0056 (0.0123) | | 0.683797987 | SLC30A10 (intergenic, 343356bp) |
| rs10799445 | 1 (225978506) | A/C | -0.0127 (0.0098) | 0.207561752 | LOC100130093 (intergenic, 4356bp) | rs10799445 | 1 (225978506) | A/C | -0.0127 (0.0098) | | 0.906142535 | LOC100130093 (intergenic, 4356bp) |
| rs4665736 | 2 (25041103) | T/C | 0.0049 (0.0138) | 0.730056588 | DNAJC27 (intron) | rs12466350 | 2 (25093473) | C/T | 0.0415 (0.0172) | | 0.010012626 | LOC729723 (intron) |
| rs6714546 | 2 (33214929) | A/G | 0.0082 (0.017) | 0.639318229 | LTBP1 (intron) | rs1545552 | 2 (33213842) | A/G | -0.0106 (0.0123) | | 0.209040525 | LTBP1 (intron) |
| rs17511102 | 2 (37814117) | A/T | -0.0272 (0.0355) | 0.45843484 | CDC42EP3 (intergenic, 61287bp) | rs17511102 | 2 (37814117) | A/T | -0.0272 (0.0355) | | 0.236644109 | CDC42EP3 (intergenic, 61287bp) |
| rs2341459 | 2 (44621706) | T/C | -0.0026 (0.0098) | 0.800447644 | C2orf34 (intron) | rs2341456 | 2 (44613445) | C/T | -0.0093 (0.0093) | | 0.172942196 | C2orf34 (intron) |
| rs12474201 | 2 (46774789) | A/G | 0.0054 (0.0137) | 0.704939483 | SOCS5 (intergenic, 4813bp) | rs17822294 | 2 (46813508) | A/G | 0.0102 (0.0127) | | 0.794958675 | SOCS5 (intron) |
| rs3791675 | 2 (55964813) | T/C | -0.0312 (0.0221) | 0.171810312 | EFEMP1 (intron) | rs1346789 | 2 (55945556) | A/G | 0.0765 (0.0192) | | 5.61908E-05 | EFEMP1 (intergenic, 1049bp) |
| rs11684404 | 2 (88705737) | T/C | -0.0111 (0.0185) | 0.562445413 | EIF2AK3 (intron) | rs6731022 | 2 (88698150) | C/G | 0.0134 (0.0122) | | 0.147279491 | EIF2AK3 (intron) |
| rs7567288 | 2 (134151294) | T/C | -0.0112 (0.0102) | 0.287314739 | NCKAP5 (intergenic, 108793bp) | rs7567288 | 2 (134151294) | C/T | -0.0112 (0.0102) | | 0.148575281 | NCKAP5 (intergenic, 108793bp) |
| rs7567851 | 2 (178392966) | C/G | -0.0043 (0.0118) | 0.724119796 | PDE11A (intron) | rs7567851 | 2 (178392966) | C/G | -0.0043 (0.0118) | | 0.652299486 | PDE11A (intron) |
| rs1351164 | 2 (217980143) | T/C | 0.0137 (0.0108) | 0.21927681 | DIRC3 (intron) | rs1351164 | 2 (217980143) | C/T | 0.0137 (0.0108) | | 0.113477962 | DIRC3 (intron) |
| rs12470505 | 2 (219616613) | T/G | 0.0232 (0.0096) | 0.019317649 | CCDC108 (intergenic, 2124bp) | rs4453686 | 2 (219604794) | C/G | 0.0395 (0.0094) | | 2.67343E-05 | CCDC108 (intron) |
| rs2580816 | 2 (232506210) | T/C | -0.0196 (0.0099) | 0.056036641 | NPPC (intergenic, 7007bp) | rs2580816 | 2 (232506210) | C/T | -0.0196 (0.0099) | | 0.029067835 | NPPC (intergenic, 7007bp) |
| rs12694997 | 2 (241911659) | A/G | -0.0115 (0.016) | 0.487410903 | SEPT2 (intron) | rs6726915 | 2 (241863422) | A/G | -0.0187 (0.0097) | | 0.031967541 | HDLBP (intron) |
| rs2597513 | 3 (13530836) | T/C | -0.0136 (0.0125) | 0.291434858 | HDAC11 (intergenic, 7913bp) | rs2630445 | 3 (13529886) | A/C | 0.0248 (0.0149) | | 0.054995551 | HDAC11 (intergenic, 6963bp) |
| rs13088462 | 3 (51046753) | T/C | -0.1555 (0.0591) | 0.010718464 | DOCK3 (intron) | rs13088462 | 3 (51046753) | C/T | -0.1555 (0.0591) | | 0.005567721 | DOCK3 (intron) |
| rs2336725 | 3 (53093779) | T/C | -0.0164 (0.0108) | 0.141934885 | RFT1 (intergenic, 3763bp) | rs4687701 | 3 (53096316) | A/G | 0.0279 (0.0107) | | 0.005990191 | RFT1 (intergenic, 1226bp) |
| rs9835332 | 3 (56642722) | C/G | -0.0401 (0.0126) | 0.002093758 | C3orf63 (missense) | rs7637449 | 3 (56603071) | A/G | 0.0597 (0.0128) | | 3.07735E-06 | CCDC66 (missense) |
| rs17806888 | 3 (67499012) | T/C | 0.0333 (0.0215) | 0.134341962 | SUCLG2 (intron) | rs12490292 | 3 (67422046) | G/T | 0.0258 (0.0165) | | 0.066631791 | MIR4272 (intergenic, 63405bp) |
| rs9863706 | 3 (72520103) | T/C | -0.0114 (0.0111) | 0.320908543 | RYBP (intron) | rs4677156 | 3 (72500547) | A/T | -0.0295 (0.0096) | | 0.001474222 | RYBP (intergenic, 5886bp) |
| rs9844666 | 3 (137456906) | A/G | 0.0003 (0.0239) | 0.991371809 | PCCB (intron) | rs9844666 | 3 (137456906) | A/G | 0.0003 (0.0239) | | 0.517555732 | PCCB (intron) |
| rs724016 | 3 (142588260) | A/G | -0.0485 (0.0107) | 1.05088E-05 | ZBTB38 (intron) | rs2871960 | 3 (142604504) | A/C | -0.0655 (0.0123) | | 1.32217E-07 | ZBTB38 (intron) |
| rs572169 | 3 (173648421) | T/C | -0.0032 (0.0199) | 0.874615843 | GHSR (synonymous) | rs572169 | 3 (173648421) | A/G | -0.0032 (0.0199) | | 0.576599874 | GHSR (synonymous) |
| rs720390 | 3 (187031377) | A/G | 0.006 (0.0094) | 0.536262093 | IGF2BP2 (intergenic, 5856bp) | rs720390 | 3 (187031377) | A/G | 0.006 (0.0094) | | 0.276600251 | IGF2BP2 (intergenic, 5856bp) |
| rs2247341 | 4 (1671115) | A/G | 0.013 (0.0149) | 0.399285787 | SLBP (synonymous) | rs8051 | 4 (1664607) | A/G | -0.0156 (0.015) | | 0.161813367 | SLBP (utr_3p) |
| rs6449353 | 4 (17642586) | T/C | 0.0312 (0.01) | 0.00250234 | LCORL (intergenic, 10005bp) | rs7663818 | 4 (17545541) | C/T | -0.0489 (0.0095) | | 2.86948E-07 | LCORL (intron) |
| rs17081935 | 4 (57518233) | T/C | 0.0021 (0.0142) | 0.88683983 | C4orf14 (intergenic, 6039bp) | rs17087335 | 4 (57533340) | G/T | 0.0447 (0.0117) | | 0.00011315 | C4orf14 (intron) |
| rs7697556 | 4 (73734177) | T/C | 0.0048 (0.0114) | 0.681361336 | ADAMTS3 (intergenic, 80797bp) | rs6852061 | 4 (73655563) | A/G | 0.0166 (0.0093) | | 0.043667085 | ADAMTS3 (intergenic, 2183bp) |
| rs788867 | 4 (82369030) | T/G | -0.029 (0.0158) | 0.075562654 | PRKG2 (intergenic, 23791bp) | rs1662840 | 4 (82375433) | A/G | 0.0513 (0.0102) | | 5.62198E-07 | PRKG2 (intergenic, 30194bp) |
| rs10010325 | 4 (106325802) | A/C | 0.0122 (0.0092) | 0.199598469 | TET2 (intron) | rs10010325 | 4 (106325802) | A/C | 0.0122 (0.0092) | | 0.103318163 | TET2 (intron) |
| rs7689420 | 4 (145787802) | T/C | -0.0229 (0.0103) | 0.030910755 | HHIP (intron) | rs1812175 | 4 (145794294) | C/T | -0.0231 (0.0103) | | 0.015646999 | HHIP (intron) |
| rs955748 | 4 (184452669) | A/G | -0.0126 (0.0093) | 0.188240291 | WWC2 (intron) | rs1473070 | 4 (184429031) | C/T | 0.0207 (0.0095) | | 0.017667714 | WWC2 (intron) |
| rs1173727 | 5 (32866278) | T/C | 0.0332 (0.0114) | 0.004647523 | C5orf23 (intergenic, 38702bp) | rs1173727 | 5 (32866278) | A/G | 0.0332 (0.0114) | | 0.002415232 | C5orf23 (intergenic, 38702bp) |
| rs11958779 | 5 (55037656) | A/G | 0.025 (0.0149) | 0.103951291 | SLC38A9 (intron) | rs7721054 | 5 (54926559) | C/T | 0.0023 (0.0098) | | 0.421675822 | SLC38A9 (intergenic, 30873bp) |
| rs10037512 | 5 (88390431) | T/C | 0.0083 (0.0116) | 0.48810006 | MEF2C (intergenic, 154753bp) | rs6452809 | 5 (88367721) | G/T | -0.0176 (0.0092) | | 0.032596201 | MEF2C (intergenic, 132043bp) |
| rs13177718 | 5 (108141243) | T/C | 0.018 (0.0381) | 0.647029732 | FER (intron) | rs13181901 | 5 (108198506) | C/T | -0.0043 (0.0334) | | 0.564013221 | FER (intron) |
| rs1582931 | 5 (122685098) | A/G | -0.0186 (0.0113) | 0.112374797 | CEP120 (intergenic, 23380bp) | rs1582931 | 5 (122685098) | C/T | -0.0186 (0.0113) | | 0.058235371 | CEP120 (intergenic, 23380bp) |
| rs274546 | 5 (131727766) | A/G | -0.0032 (0.0104) | 0.764392623 | SLC22A5 (intergenic, 5533bp) | rs274558 | 5 (131749073) | C/T | 0.0122 (0.0095) | | 0.111796731 | SLC22A5 (synonymous) |
| rs526896 | 5 (134384604) | T/G | -0.0377 (0.0143) | 0.010577514 | PITX1 (intergenic, 6718bp) | rs299363 | 5 (134359721) | A/G | 0.0228 (0.0162) | | 0.922726419 | CATSPER3 (intron) |
| rs4282339 | 5 (168188818) | A/G | -0.0457 (0.0132) | 0.000776846 | SLIT3 (intron) | rs4282339 | 5 (168188818) | A/G | -0.0457 (0.0132) | | 0.000403978 | SLIT3 (intron) |
| rs12153391 | 5 (171136043) | A/C | -0.0272 (0.0157) | 0.092863145 | FBXW11 (intergenic, 85117bp) | rs12153391 | 5 (171136043) | A/C | -0.0272 (0.0157) | | 0.048138533 | FBXW11 (intergenic, 85117bp) |
| rs889014 | 5 (172916720) | T/C | 0.0042 (0.0093) | 0.661470126 | LOC285593 (intergenic, 22531bp) | rs6885032 | 5 (172915885) | C/G | 0.0065 (0.0093) | | 0.765024579 | LOC285593 (intergenic, 23366bp) |
| rs422421 | 5 (176449932) | T/C | 0 (0.0108) | 0.998254975 | FGFR4 (intron) | rs376618 | 5 (176450403) | A/G | 0.0035 (0.0099) | | 0.647908102 | FGFR4 (missense) |
| rs6879260 | 5 (179663620) | T/C | -0.011 (0.0095) | 0.260721466 | GFPT2 (intron) | rs6879260 | 5 (179663620) | C/T | -0.011 (0.0095) | | 0.13486269 | GFPT2 (intron) |
| rs3812163 | 6 (7670759) | A/T | -0.0277 (0.0102) | 0.008716645 | BMP6 (near_gene_5p_(2000_bp)) | rs3812163 | 6 (7670759) | A/T | -0.0277 (0.0102) | | 0.004528421 | BMP6 (near_gene_5p_(2000_bp)) |
| rs1047014 | 6 (19949472) | T/C | -0.0155 (0.0119) | 0.209193473 | ID4 (intergenic, 578bp) | rs1047014 | 6 (19949472) | A/G | -0.0155 (0.0119) | | 0.108272488 | ID4 (intergenic, 578bp) |
| rs806794 | 6 (26308656) | A/G | 0.0109 (0.0096) | 0.270203289 | HIST1H3D (near_gene_5p_(2000_bp)) | rs806794 | 6 (26308656) | A/G | 0.0109 (0.0096) | | 0.139752712 | HIST1H3D (near_gene_5p_(2000_bp)) |
| rs3129109 | 6 (29192211) | T/C | -0.015 (0.0139) | 0.295954133 | OR2J3 (intergenic, 3629bp) | rs6901599 | 6 (29034724) | C/T | 0.0236 (0.0127) | | 0.037441283 | TRIM27 (intergenic, 34977bp) |
| rs2256183 | 6 (31488508) | A/G | 0.0087 (0.01) | 0.396706911 | MICA (intron) | rs2853977 | 6 (31487283) | A/T | 0.0273 (0.0102) | | 0.004802718 | MICA (intron) |
| rs6457620 | 6 (32771977) | C/G | -0.0105 (0.0092) | 0.268287937 | HLA-DQB1 (intergenic, 29533bp) | rs4713582 | 6 (32768029) | C/T | 0.0114 (0.0093) | | 0.120347634 | HLA-DQB1 (intergenic, 25585bp) |
| rs2780226 | 6 (34307070) | T/C | -0.0338 (0.0097) | 0.000723996 | HMGA1 (intergenic, 5484bp) | rs12214804 | 6 (34296844) | C/T | -0.0237 (0.01) | | 0.011124009 | HMGA1 (intergenic, 15710bp) |
| rs6457821 | 6 (35510783) | A/C | 0.0075 (0.0169) | 0.665566588 | PPARD (intergenic, 6839bp) | rs9658134 | 6 (35486776) | A/G | -0.004 (0.0347) | | 0.468100518 | PPARD (utr_5p) |
| rs9472414 | 6 (45054484) | A/T | -0.0154 (0.0108) | 0.165836244 | SUPT3H (intron) | rs12530016 | 6 (44974300) | A/G | -0.0231 (0.0125) | | 0.038109089 | SUPT3H (intron) |
| rs9360921 | 6 (76322362) | T/G | -0.0247 (0.0143) | 0.09393459 | SENP6 (intergenic, 45979bp) | rs10943249 | 6 (76255939) | C/T | 0.0399 (0.0146) | | 0.004131538 | FILIP1 (intron) |
| rs310405 | 6 (81857081) | A/G | 0.0142 (0.0106) | 0.193565152 | FAM46A (intergenic, 655084bp) | rs310426 | 6 (81846637) | A/G | 0.0192 (0.0107) | | 0.041743295 | FAM46A (intergenic, 665528bp) |
| rs961764 | 6 (117628849) | C/G | -0.0052 (0.0098) | 0.609130859 | VGLL2 (intergenic, 64564bp) | rs4946230 | 6 (117609470) | A/C | -0.0179 (0.0128) | | 0.09114326 | VGLL2 (intergenic, 83943bp) |
| rs6569648 | 6 (130390812) | T/C | -0.0222 (0.0234) | 0.358639469 | L3MBTL3 (intron) | rs6569648 | 6 (130390812) | C/T | -0.0222 (0.0234) | | 0.185318854 | L3MBTL3 (intron) |
| rs9456307 | 6 (158849430) | A/T | -0.0102 (0.0143) | 0.489379835 | TULP4 (utr_3p) | rs10447365 | 6 (158806875) | A/G | -0.081 (0.0418) | | 0.031281977 | TULP4 (intron) |
| rs798489 | 7 (2768329) | T/C | 0.0035 (0.0358) | 0.924665905 | GNA12 (intron) | rs798498 | 7 (2762408) | A/C | 0.0181 (0.0108) | | 0.054499166 | GNA12 (intron) |
| rs4470914 | 7 (19583047) | T/C | 0.0056 (0.0098) | 0.57801963 | TWISTNB (intergenic, 118562bp) | rs2390151 | 7 (19608625) | G/T | 0.0116 (0.0095) | | 0.122127348 | TWISTNB (intergenic, 92984bp) |
| rs12534093 | 7 (23469499) | A/T | -0.0075 (0.0145) | 0.617829776 | IGF2BP3 (intron) | rs12540730 | 7 (23450340) | A/G | 0.0279 (0.0175) | | 0.063280364 | IGF2BP3 (intron) |
| rs1708299 | 7 (28156471) | A/G | -0.0029 (0.014) | 0.840455521 | JAZF1 (intron) | rs506154 | 7 (28168604) | C/T | 0.0321 (0.017) | | 0.035003765 | JAZF1 (intron) |
| rs6959212 | 7 (38094851) | T/C | -0.011 (0.0097) | 0.268187112 | STARD3NL (intergenic, 89606bp) | rs940347 | 7 (38076950) | C/T | -0.0133 (0.0096) | | 0.092841922 | STARD3NL (intergenic, 107507bp) |
| rs822552 | 7 (148281567) | C/G | 0.0008 (0.0102) | 0.9372719 | PDIA4 (intergenic, 49519bp) | rs822553 | 7 (148281751) | A/G | -0.007 (0.0171) | | 0.667760858 | PDIA4 (intergenic, 49335bp) |
| rs2110001 | 7 (150147955) | C/G | -0.002 (0.0096) | 0.843464668 | TMEM176A (intergenic, 14814bp) | rs2110001 | 7 (150147955) | 0/C | -0.002 (0.0096) | | 0.433644858 | TMEM176A (intergenic, 14814bp) |
| rs1013209 | 8 (24172249) | T/C | 0.0036 (0.0114) | 0.758558695 | ADAM28 (intergenic, 35275bp) | rs2089827 | 8 (24176232) | A/G | 0.0293 (0.0175) | | 0.053806004 | ADAM28 (intergenic, 31292bp) |
| rs7460090 | 8 (57356717) | T/C | 0.0192 (0.0121) | 0.122231997 | SDR16C5 (intergenic, 18406bp) | rs7815925 | 8 (57362936) | A/G | 0.0175 (0.0128) | | 0.916762312 | SDR16C5 (intergenic, 12187bp) |
| rs6473015 | 8 (78341040) | A/C | 0.0055 (0.0135) | 0.694224498 | PEX2 (intergenic, 265205bp) | rs11778981 | 8 (78274739) | C/G | 0.0115 (0.0174) | | 0.268842442 | PEX2 (intergenic, 198904bp) |
| rs6470764 | 8 (130794847) | T/C | -0.0392 (0.0111) | 0.000610668 | GSDMC (intergenic, 34776bp) | rs4733724 | 8 (130792910) | A/G | 0.0442 (0.0113) | | 7.62167E-05 | GSDMC (intergenic, 36713bp) |
| rs12680655 | 8 (135706519) | C/G | 0.0212 (0.0095) | 0.031326957 | ZFAT (intron) | rs2277138 | 8 (135691822) | A/G | 0.0458 (0.0121) | | 0.000122654 | ZFAT (intron) |
| rs7864648 | 9 (16358732) | T/G | 0.0038 (0.0116) | 0.750000671 | BNC2 (intergenic, 40768bp) | rs10756751 | 9 (16361985) | C/G | -0.009 (0.0099) | | 0.822154645 | BNC2 (intergenic, 37515bp) |
| rs11144688 | 9 (77732106) | A/G | -0.0753 (0.0312) | 0.019202129 | PCSK5 (intron) | rs11144688 | 9 (77732106) | A/G | -0.0753 (0.0312) | | 0.009970666 | PCSK5 (intron) |
| rs7853377 | 9 (85742025) | A/G | -0.0166 (0.0121) | 0.181574206 | C9orf64 (intergenic, 1021bp) | rs10868080 | 9 (85816589) | A/T | -0.0213 (0.0114) | | 0.035813549 | RMI1 (intergenic, 7782bp) |
| rs8181166 | 9 (88306448) | C/G | 0.0199 (0.0128) | 0.13236228 | ZCCHC6 (intergenic, 147226bp) | rs405761 | 9 (88274946) | C/T | 0.0372 (0.01) | | 0.000161102 | ZCCHC6 (intergenic, 115724bp) |
| rs2778031 | 9 (90025546) | T/C | 0.012 (0.0123) | 0.343388052 | FAM75C2 (intergenic, 85826bp) | rs2482488 | 9 (90011241) | A/G | -0.0236 (0.0094) | | 0.007943787 | FAM75C2 (intergenic, 71521bp) |
| rs9969804 | 9 (94468941) | A/C | 0.0215 (0.0185) | 0.259913802 | IPPK (intron) | rs10120210 | 9 (94324803) | G/T | 0.0223 (0.0149) | | 0.075669904 | ECM2 (missense) |
| rs473902 | 9 (97296056) | T/G | 0.0364 (0.0376) | 0.347477682 | PTCH1 (intron) | rs473902 | 9 (97296056) | A/C | 0.0364 (0.0376) | | 0.179572122 | PTCH1 (intron) |
| rs7027110 | 9 (108638867) | A/G | 0.0108 (0.0124) | 0.395218804 | ZNF462 (intergenic, 26331bp) | rs7025923 | 9 (108610548) | A/G | -0.0158 (0.0119) | | 0.101470425 | ZNF462 (intergenic, 54650bp) |
| rs1468758 | 9 (112846903) | T/C | -0.0208 (0.0135) | 0.13684053 | LPAR1 (intergenic, 6717bp) | rs10817133 | 9 (112831795) | A/G | 0.0463 (0.0129) | | 0.000250931 | LPAR1 (intron) |
| rs751543 | 9 (118162163) | T/C | 0.0219 (0.0092) | 0.021114915 | PAPPA (intron) | rs751543 | 9 (118162163) | A/G | 0.0219 (0.0092) | | 0.010963086 | PAPPA (intron) |
| rs7466269 | 9 (132453905) | A/G | -0.0065 (0.011) | 0.568324003 | FUBP3 (intron) | rs7021911 | 9 (132490839) | C/T | -0.0199 (0.0347) | | 0.297811199 | FUBP3 (intron) |
| rs7849585 | 9 (138251691) | T/G | 0.0096 (0.0113) | 0.409892173 | QSOX2 (intron) | rs12338076 | 9 (138261561) | A/C | -0.0273 (0.0124) | | 0.016766788 | QSOX2 (intron) |
| rs7909670 | 10 (12958770) | T/C | -0.0167 (0.0124) | 0.190596381 | CCDC3 (intergenic, 19860bp) | rs7909670 | 10 (12958770) | C/T | -0.0167 (0.0124) | | 0.098669114 | CCDC3 (intergenic, 19860bp) |
| rs2145998 | 10 (80791702) | A/T | -0.0339 (0.0094) | 0.000474598 | PPIF (intergenic, 6607bp) | rs941873 | 10 (80809468) | C/T | -0.0541 (0.0103) | | 1.86295E-07 | ZCCHC24 (intergenic, 2622bp) |
| rs11599750 | 10 (101795432) | T/C | -0.0065 (0.0093) | 0.499318812 | CPN1 (intron) | rs7084921 | 10 (101803792) | C/T | -0.0071 (0.0093) | | 0.237708595 | CPN1 (intron) |
| rs2237886 | 11 (2767307) | T/C | -0.0279 (0.0217) | 0.213575776 | KCNQ1 (intron) | rs234886 | 11 (2758666) | C/G | 0.0104 (0.0137) | | 0.782132324 | KCNQ1 (intron) |
| rs7926971 | 11 (12654616) | A/G | -0.0113 (0.0101) | 0.279871808 | TEAD1 (intron) | rs10831894 | 11 (12637309) | A/G | 0.0156 (0.0097) | | 0.061004727 | TEAD1 (intergenic, 15235bp) |
| rs1330 | 11 (17272605) | T/C | 0.014 (0.0122) | 0.267481293 | NUCB2 (intron) | rs1330 | 11 (17272605) | A/G | | 0.014 (0.0122) | 0.138349002 | NUCB2 (intron) |
| rs1814175 | 11 (49515748) | T/C | -0.0127 (0.0105) | 0.24242012 | LOC440040 (intergenic, 20907bp) | rs1814175 | 11 (49515748) | C/T | | -0.0127 (0.0105) | 0.889507528 | LOC440040 (intergenic, 20907bp) |
| rs5017948 | 11 (51270794) | A/T | -0.0055 (0.0111) | 0.63160423 | OR4A5 (near_gene_5p_(2000_bp)) | rs5017948 | 11 (51270794) | A/T | | -0.0055 (0.0111) | 0.698559659 | OR4A5 (near_gene_5p_(2000_bp)) |
| rs3782089 | 11 (65093395) | T/C | 0.0062 (0.0257) | 0.816570948 | SSSCA1 (near_gene_5p_(2000_bp)) | rs3782089 | 11 (65093395) | A/G | | 0.0062 (0.0257) | 0.605855638 | SSSCA1 (near_gene_5p_(2000_bp)) |
| rs634552 | 11 (74959700) | T/G | 0.0159 (0.0099) | 0.119509666 | SERPINH1 (intron) | rs606452 | 11 (74953826) | A/C | | 0.0425 (0.01) | 1.81249E-05 | SERPINH1 (intron) |
| rs494459 | 11 (118079885) | T/C | 0.0118 (0.0114) | 0.318607023 | TREH (intergenic, 24294bp) | rs625735 | 11 (118080629) | C/T | | 0.0131 (0.0114) | 0.138816983 | TREH (intergenic, 25038bp) |
| rs654723 | 11 (128091365) | A/C | 0.02 (0.0101) | 0.054084417 | FLI1 (intron) | rs654723 | 11 (128091365) | A/C | | 0.02 (0.0101) | 0.028056313 | FLI1 (intron) |
| rs2856321 | 12 (11747040) | A/G | -0.0115 (0.0144) | 0.437796522 | ETV6 (intron) | rs762717 | 12 (11759837) | G/T | | -0.0368 (0.0149) | 0.008580055 | ETV6 (intron) |
| rs10770705 | 12 (20748734) | A/C | 0.0288 (0.0108) | 0.009899913 | SLCO1C1 (intron) | rs7305275 | 12 (20725902) | C/T | | 0.0459 (0.013) | 0.00031927 | PDE3A (intergenic, 754bp) |
| rs2638953 | 12 (28425682) | C/G | 0.0029 (0.0097) | 0.772169865 | CCDC91 (intron) | rs11049460 | 12 (28250221) | A/G | | -0.0282 (0.0109) | 0.006368515 | CCDC91 (intergenic, 51178bp) |
| rs1351394 | 12 (64638093) | T/C | 0.0392 (0.0093) | 4.71085E-05 | HMGA2 (intron) | rs7968682 | 12 (64658147) | G/T | | -0.0426 (0.0093) | 4.70714E-06 | HMGA2 (intergenic, 11809bp) |
| rs10748128 | 12 (68113925) | T/G | 0.0177 (0.0119) | 0.14971512 | FRS2 (intergenic, 36470bp) | rs10748128 | 12 (68113925) | G/T | | 0.0177 (0.0119) | 0.077545516 | FRS2 (intergenic, 36470bp) |
| rs11107116 | 12 (92502635) | T/G | 0.0461 (0.0164) | 0.00652218 | SOCS2 (intergenic, 8526bp) | rs11107116 | 12 (92502635) | G/T | | 0.0461 (0.0164) | 0.003388895 | SOCS2 (intergenic, 8526bp) |
| rs7971536 | 12 (100897919) | A/T | 0.001 (0.0099) | 0.920689927 | CCDC53 (intergenic, 32928bp) | rs7978999 | 12 (100892196) | C/T | | 0.0272 (0.0118) | 0.013381621 | CCDC53 (intergenic, 38651bp) |
| rs7332115 | 13 (32045548) | T/G | -0.007 (0.0099) | 0.488592295 | PDS5B (intergenic, 13015bp) | rs731413 | 13 (32073656) | A/G | | 0.0228 (0.0093) | 0.008984635 | PDS5B (intron) |
| rs3118905 | 13 (50003335) | A/G | -0.0206 (0.015) | 0.182805701 | DLEU7 (intergenic, 181424bp) | rs3118906 | 13 (50004789) | A/G | | -0.0274 (0.0151) | 0.040591159 | DLEU7 (intergenic, 179970bp) |
| rs7319045 | 13 (90822575) | A/G | 0.0275 (0.0094) | 0.004538457 | MIR17HG (intergenic, 17745bp) | rs7319045 | 13 (90822575) | A/G | | 0.0275 (0.0094) | 0.002358578 | MIR17HG (intergenic, 17745bp) |
| rs1950500 | 14 (23900690) | T/C | 0.0167 (0.0096) | 0.090952059 | NFATC4 (intergenic, 5294bp) | rs12590407 | 14 (23904955) | A/G | | -0.0231 (0.0096) | 0.010156409 | NFATC4 (near_gene_5p_(2000_bp)) |
| rs2093210 | 14 (60027032) | T/C | -0.0094 (0.0147) | 0.534593632 | C14orf39 (intergenic, 4515bp) | rs2093210 | 14 (60027032) | C/T | | -0.0094 (0.0147) | 0.275744396 | C14orf39 (intergenic, 4515bp) |
| rs1570106 | 14 (67882868) | T/C | -0.0058 (0.0198) | 0.774891551 | RAD51L1 (intron) | rs6573834 | 14 (67878151) | C/T | | -0.0338 (0.0149) | 0.014323073 | RAD51L1 (intron) |
| rs862034 | 14 (74060499) | A/G | -0.0317 (0.0121) | 0.010897487 | LTBP2 (intron) | rs862057 | 14 (74048618) | A/G | | 0.04 (0.0095) | 0.000022838 | LTBP2 (intron) |
| rs7155279 | 14 (91555634) | T/G | -0.0172 (0.0111) | 0.13236228 | TRIP11 (intron) | rs7157056 | 14 (91567786) | A/G | | 0.0179 (0.0116) | 0.940083862 | TRIP11 (intron) |
| rs16964211 | 15 (49317787) | A/G | -0.007 (0.0102) | 0.504826621 | CYP19A1 (intron) | rs16964220 | 15 (49330674) | A/G | | -0.0136 (0.0099) | 0.094485979 | CYP19A1 (intron) |
| rs7178424 | 15 (60167551) | T/C | -0.0061 (0.01) | 0.550976663 | C2CD4A (intergenic, 17143bp) | rs7172967 | 15 (60005860) | A/C | | -0.0299 (0.0119) | 0.007724976 | VPS13C (intron) |
| rs10152591 | 15 (67835211) | A/C | 0.0039 (0.0142) | 0.789469057 | C15orf50 (intergenic, 79415bp) | rs7181071 | 15 (67784396) | C/T | | -0.0221 (0.0152) | 0.08198407 | C15orf50 (intergenic, 130230bp) |
| rs5742915 | 15 (72123686) | T/C | -0.0498 (0.0194) | 0.01296857 | PML (missense) | rs5742915 | 15 (72123686) | C/T | | -0.0498 (0.0194) | 0.006735738 | PML (missense) |
| rs11259936 | 15 (82371586) | A/C | -0.034 (0.0112) | 0.003396551 | ADAMTSL3 (intron) | rs1564472 | 15 (82360797) | C/T | | -0.0355 (0.0112) | 0.001120475 | ADAMTSL3 (intron) |
| rs2871865 | 15 (97012419) | C/G | 0.0191 (0.0106) | 0.079974942 | IGF1R (intron) | rs2871865 | 15 (97012419) | C/G | | 0.0191 (0.0106) | 0.041466498 | IGF1R (intron) |
| rs4965598 | 15 (98577137) | T/C | -0.0113 (0.0151) | 0.466522937 | ADAMTS17 (intron) | rs4965599 | 15 (98577407) | C/G | | -0.0145 (0.01) | 0.082784867 | ADAMTS17 (intron) |
| rs26868 | 16 (2189377) | A/T | 0.0161 (0.019) | 0.409991248 | CASKIN1 (intergenic, 2911bp) | rs26868 | 16 (2189377) | A/T | | 0.0161 (0.019) | 0.211741818 | CASKIN1 (intergenic, 2911bp) |
| rs1659127 | 16 (14295806) | A/G | 0.034 (0.0106) | 0.001794521 | MIR193B (intergenic, 9518bp) | rs1659127 | 16 (14295806) | A/G | | 0.034 (0.0106) | 0.00093294 | MIR193B (intergenic, 9518bp) |
| rs8052560 | 16 (87304743) | A/C | 0.0187 (0.0152) | 0.234413054 | CTU2 (intron) | rs8052560 | 16 (87304743) | A/C | | 0.0187 (0.0152) | 0.121289999 | CTU2 (intron) |
| rs4640244 | 17 (21224816) | A/G | 0.0404 (0.0122) | 0.001312637 | KCNJ12 (intron) | rs4640244 | 17 (21224816) | A/G | | 0.0404 (0.0122) | 0.00068249 | KCNJ12 (intron) |
| rs3110496 | 17 (24941897) | A/G | -0.0144 (0.0095) | 0.141934885 | GIT1 (near_gene_5p_(2000_bp)) | rs565977 | 17 (24922954) | C/T | | -0.0192 (0.0115) | 0.055709709 | TP53I13 (intron) |
| rs3764419 | 17 (26188149) | A/C | -0.0322 (0.0106) | 0.003209979 | ATAD5 (intron) | rs7225461 | 17 (26284025) | A/G | | -0.0348 (0.011) | 0.001097993 | ADAP2 (intron) |
| rs17780086 | 17 (27367395) | A/G | 0.0368 (0.0278) | 0.199598469 | LRRC37B (intergenic, 4872bp) | rs560132 | 17 (27344132) | A/G | | 0.039 (0.0275) | 0.087953845 | SUZ12 (intron) |
| rs1043515 | 17 (34175722) | A/G | -0.0138 (0.0102) | 0.189879456 | PIP4K2B (utr_3p) | rs1043515 | 17 (34175722) | C/T | | -0.0138 (0.0102) | 0.098298829 | PIP4K2B (utr_3p) |
| rs4986172 | 17 (40571807) | T/C | 0.02 (0.0121) | 0.106871709 | ACBD4 (intron) | rs4986172 | 17 (40571807) | C/T | | 0.02 (0.0121) | 0.953333415 | ACBD4 (intron) |
| rs2072153 | 17 (44745013) | C/G | -0.005 (0.0184) | 0.794132845 | ZNF652 (intron) | rs11652146 | 17 (44777362) | A/G | | -0.0068 (0.0095) | 0.251982629 | ZNF652 (intron) |
| rs4605213 | 17 (46599746) | C/G | 0.0152 (0.0129) | 0.254964416 | NME2 (intron) | rs4605213 | 17 (46599746) | C/G | | 0.0152 (0.0129) | 0.131893193 | NME2 (intron) |
| rs227724 | 17 (52133816) | A/T | 0.0242 (0.0111) | 0.033974699 | C17orf67 (intergenic, 90456bp) | rs227724 | 17 (52133816) | A/T | | 0.0242 (0.0111) | 0.986073685 | C17orf67 (intergenic, 90456bp) |
| rs2079795 | 17 (56851431) | T/C | 0.0065 (0.0094) | 0.500794319 | C17orf82 (intergenic, 6008bp) | rs740755 | 17 (56851579) | C/G | | 0.0127 (0.0094) | 0.099197971 | C17orf82 (intergenic, 6156bp) |
| rs2665838 | 17 (59320197) | C/G | -0.0107 (0.0114) | 0.366305058 | CSH1 (intergenic, 5807bp) | rs2854207 | 17 (59300839) | C/G | | -0.0271 (0.0096) | 0.003150425 | CSH2 (intergenic, 2264bp) |
| rs11867479 | 17 (65601802) | T/C | 0.0015 (0.0142) | 0.917295699 | KCNJ16 (intron) | rs11867479 | 17 (65601802) | C/T | | 0.0015 (0.0142) | 0.471205829 | KCNJ16 (intron) |
| rs4800452 | 18 (18981609) | T/C | 0.0126 (0.0125) | 0.325409537 | CABLES1 (intron) | rs4800455 | 18 (19002731) | A/G | | -0.0285 (0.0117) | 0.009556523 | CABLES1 (intron) |
| rs9967417 | 18 (45213498) | C/G | -0.0253 (0.0117) | 0.035975385 | DYM (intron) | rs11663141 | 18 (45198465) | A/G | | 0.0324 (0.0121) | 0.004899567 | DYM (intron) |
| rs17782313 | 18 (56002077) | T/C | 0.0021 (0.0102) | 0.844338264 | MC4R (intergenic, 187466bp) | rs12970134 | 18 (56035730) | A/G | | 0.0254 (0.0133) | 0.03306726 | MC4R (intergenic, 153813bp) |
| rs12982744 | 19 (2128193) | C/G | 0.0097 (0.0279) | 0.736868026 | DOT1L (intron) | rs2864419 | 19 (2134175) | G/T | | 0.0127 (0.0093) | 0.094538989 | DOT1L (intron) |
| rs891088 | 19 (7135762) | A/G | -0.0103 (0.0093) | 0.28409721 | INSR (intron) | rs891088 | 19 (7135762) | A/G | | -0.0103 (0.0093) | 0.14691657 | INSR (intron) |
| rs4072910 | 19 (8550031) | C/G | -0.0135 (0.0127) | 0.299467014 | MYO1F (near_gene_5p_(2000_bp)) | rs4072910 | 19 (8550031) | C/G | | -0.0135 (0.0127) | 0.154839178 | MYO1F (near_gene_5p_(2000_bp)) |
| rs2279008 | 19 (17144303) | T/C | 0.0468 (0.0156) | 0.003674033 | MYO9B (intron) | rs2279008 | 19 (17144303) | A/G | | 0.0468 (0.0156) | 0.001909529 | MYO9B (intron) |
| rs17318596 | 19 (46628935) | A/G | 0.0153 (0.0117) | 0.203888192 | ATP5SL (near_gene_3p_(500_bp)) | rs17318596 | 19 (46628935) | A/G | | 0.0153 (0.0117) | 0.105533255 | ATP5SL (near_gene_3p_(500_bp)) |
| rs1741344 | 20 (4049800) | T/C | -0.0195 (0.0094) | 0.043424105 | SMOX (intergenic, 27649bp) | rs1741344 | 20 (4049800) | C/T | | -0.0195 (0.0094) | 0.022531682 | SMOX (intergenic, 27649bp) |
| rs2145272 | 20 (6574218) | A/G | -0.0025 (0.0123) | 0.842882264 | BMP2 (intergenic, 122526bp) | rs979012 | 20 (6571374) | A/G | | 0.0243 (0.0102) | 0.011088259 | BMP2 (intergenic, 125370bp) |
| rs143384 | 20 (33489170) | A/G | -0.0457 (0.018) | 0.013935162 | GDF5 (utr_5p) | rs6088791 | 20 (33371323) | C/T | | -0.0311 (0.0102) | 0.001623846 | UQCC (intron) |
| rs237743 | 20 (47336426) | A/G | 0.0168 (0.0102) | 0.108346187 | NCRNA00275 (intron) | rs8123912 | 20 (47296689) | C/G | | 0.0237 (0.0119) | 0.027733789 | ZNFX1 (utr_3p) |
| rs2834442 | 21 (34612656) | A/T | 0.0154 (0.0097) | 0.121918038 | KCNE2 (intergenic, 45536bp) | rs2834440 | 21 (34612369) | A/G | | 0.0254 (0.0096) | 0.005384683 | KCNE2 (intergenic, 45823bp) |
| rs4821083 | 22 (31386341) | T/C | 0.04 (0.0094) | 3.68574E-05 | SYN3 (intron) | rs3788478 | 22 (31387746) | A/G | | 0.0414 (0.0094) | 1.03878E-05 | SYN3 (intron) |
