# Supplementary material for: Identification, Replication, and Fine-Mapping of Loci Associated with Adult Height in Individuals of African Ancestry
Source: PLoS Genet. 2011 Oct 6;7(10):e1002298. doi: 10.1371/journal.pgen.1002298 (PMC3188544; doi:10.1371/journal.pgen.1002298)
Supplement: Text S1 — Supplementary information. (DOC) [file pgen.1002298.s006.doc]

**Text S1**

**Identification, replication and fine-mapping of loci associated with adult height in individuals of African ancestry**

Amidou N’Diaye, Gary K. Chen, Cameron D. Palmer, Bing Ge, Bamidele Tayo, Rasika A. Mathias, Jingzhong Ding, Michael A. Nalls, Adebowale Adeyemo, Véronique Adoue, Christine B. Ambrosone, Larry Atwood, Elisa V. Bandera, Lewis C. Becker, Sonja I. Berndt, Leslie Bernstein, William J. Blot, Eric Boerwinkle, Angela Britton, Graham Casey, Stephen J. Chanock, Ellen Demerath, Angela DeMichele, Sandra L. Deming, W. Ryan Diver, Caroline Fox, Tamara B. Harris, Dena G. Hernandez, Jennifer J. Hu, Sue A. Ingles, Esther M. John, Craig Johnson, Brendan Keating, Rick A. Kittles, Laurence N. Kolonel, Stephen B. Kritchevsky, Loic Le Marchand, Kurt Lohman, Jiankang Liu, Robert C. Millikan, Adam Murphy, Solomon Musani, Christine Neslund-Dudas, Kari E. North, Sarah Nyante, Adesola Ogunniyi, Elaine A. Ostrander, George Papanicolaou, Sanjay Patel, Curtis A. Pettaway, Michael F. Press, Susan Redline, Jorge L. Rodriguez-Gil, Charles Rotimi, Benjamin A. Rybicki, Babatunde Salako, Pamela J. Schreiner, Lisa B. Signorello, Andrew B. Singleton, Janet L. Stanford, Alex Stram, Sara S. Strom, Bhoom Suktitipat, Michael J. Thun, John S. Witte, Lisa R. Yanek, Regina G. Ziegler, Wei Zheng, Xiaofeng Zhu, Joseph M. Zmuda, Alan B. Zonderman, Michele K. Evans, Yongmei Liu, Diane M. Becker, Richard S. Cooper, Tomi Pastinen, Brian E. Henderson, Joel N. Hirschhorn, Guillaume Lettre, Christopher A. Haiman

**Discovery studies**

Baseline characteristics including sample size, mean value and standard deviation of age and height for each study are provided in Table S1.

- 1. ***Genome-wide association study of breast cancer in African Americans (AABC)***

AABC consisted of 9 studies. In each study, information on height was based on self-report.

T*he Multiethnic Cohort Study (MEC):* The MEC is a prospective cohort study of 215,000 men and women in Hawaii and Los Angeles [[1]](#_ENREF_1) between the ages of 45 and 75 years at baseline (1993-1996). Through December, 31 2007, a nested breast cancer (BC) case-control study in the MEC included 556 African American cases (544 invasive and 12 in situ) and 1,003 African American controls. An additional 178 African American breast cancer cases (ages: 50-84 years) diagnosed between June 1, 2006 and December 31, 2007 in Los Angeles County (but outside of the MEC) were combined with the MEC samples in the analysis.

*The Los Angeles component of The Women’s Contraceptive and Reproductive Experiences (CARE) Study:* The NICHD Women's CARE Study is a large multi-center population-based case-control study that was designed to examine the effects of oral contraceptive use on invasive breast cancer risk among African American women and white women ages 35-64 years in five U.S. locations [[2]](#_ENREF_2). Cases in Los Angeles County were diagnosed from July 1, 1994 through April 30, 1998, and controls were sampled by random-digit dialing (RDD) from the same population and time period; 380 African American cases and 224 African American controls were included in stage 1 of the scan.

*The Women’s Circle of Health Study (WCHS):* The WCHS is an ongoing case-control study of breast cancer among white women and African American women in the New York City boroughs (Manhattan, the Bronx, Brooklyn and Queens) and in seven counties in New Jersey (Bergen, Essex, Hudson, Mercer, Middlesex, Passaic, and Union) [[3]](#_ENREF_3). Eligible cases included women with invasive breast cancer between 20 and 74 years of age; controls were identified through RDD. The WCHS contributed 272 invasive African American cases and 240 African American controls to stage 1 of the GWAS.

*The San Francisco Bay Area Breast Cancer Study (SFBCS):* The SFBCS is a population-based case-control study of invasive breast cancer in Hispanic, African American and non-Hispanic White women conducted between 1995 and 2003 in the San Francisco Bay Area [[4]](#_ENREF_4). African American cases, ages 35-79 years, were diagnosed between April 1, 1995 and April 30, 1999, with controls identified through RDD. Stage 1 included 172 invasive African American cases and 231 African American controls from the SFBCS.

*The Northern California Breast Cancer Family Registry (NC-BCFR):* The NC-BCFR is an on-going population-based family study conducted in the Greater San Francisco Bay Area, and is one of 6 sites collaborating in the Breast Cancer Family Registry (BCFR), an international consortium funded by NCI [5]. African American breast cancer cases in NC-BCFR were diagnosed after January 1, 1995 and between the ages of 18 and 64 years; population controls were identified through RDD. Stage 1 genotyping was conducted for 440 invasive African American cases and 53 African American controls.

*The Carolina Breast Cancer Study (CBCS):* The CBCS is a population-based case-control study conducted between 1993 and 2001 in 24 counties of central and eastern North Carolina [[6]](#_ENREF_6). Cases were identified by rapid case ascertainment system in cooperation with the North Carolina Central Cancer Registry and controls were selected from the North Carolina Division of Motor Vehicle and United States Health Care Financing Administration beneficiary lists. Participants’ ages ranged from 20 to 74 years. For stage 1, DNA samples were provided from 656 African American cases with invasive breast cancer and 608 African American controls.

*The Prostate, Lung, Colorectal, and Ovarian Cancer Screening Trial (PLCO) Cohort*: PLCO, coordinated by the U.S. National Cancer Institute (NCI) in 10 U.S. centers, enrolled during 1993 - 2001 approximately 155,000 men and women, aged 55-74 years, in a randomized, two-arm trial to evaluate the efficacy of screening for these four cancers [[7]](#_ENREF_7). A total of 64 African American invasive breast cancer cases and 133 African American controls contributed to stage 1 of the GWAS.

*The Nashville Breast Health Study (NBHS):* The NBHS is a population-based case-control study of breast cancer conducted in Tennessee [[8]](#_ENREF_8). The study was initiated in 2001 to recruit patients with invasive breast cancer or ductal carcinoma in situ, and controls, recruited through RDD between the ages of 25 and 75 years. NBHS contributed 310 African American cases (57 in situ), and 186 African American controls to stage 1 of the GWAS.

*Wake Forest University Breast Cancer Study (WFBC):* African American breast cancer cases and controls in WFBC were recruited at Wake Forest University Health Sciences from November 1998 through December 2008 [[9]](#_ENREF_9). Controls were recruited from the patient population receiving routine mammography at the Breast Screening and Diagnostic Center. Age range of participants was 30-86 years. WFBC contributed 125 cases (116 invasive and 9 in situ) and 153 controls to the stage 1 analysis.

- 1. ***Genome-wide association study of prostate cancer in African Americans (AAPC)***

AAPC is composed of 10 studies. In each study, information on height was based on self-report.

*The Multiethnic Cohort (MEC):* The MEC is discussed above [[1]](#_ENREF_1). Through January 1, 2008 the African American prostate cancer (PC) case-control study in the MEC included 1,094 cases and 1,096 controls.

*The Southern Community Cohort Study (SCCS):* The SCCS is a prospective cohort of African and non-African Americans which during 2002-2009 enrolled approximately 86,000 residents aged 40-79 years across 12 southern states [[10]](#_ENREF_10).  Recruitment occurred mainly at community health centers, institutions providing basic health services primarily to the medically uninsured, so that the cohort includes many adults of lower income and educational status. Each study participant completed a detailed baseline questionnaire, and nearly 90% provided a biologic specimen (approximately 45% a blood sample and 45% buccal cells). Follow-up of the cohort is conducted by linkage to national mortality registers and to state cancer registries. Included in this study are 212 incident African American prostate cancer cases and a matched stratified random sample of 419 African American male cohort members without prostate cancer at the index date selected by incidence density sampling.

*The Prostate, Lung, Colorectal, and Ovarian Cancer Screening Trial (PLCO):*The Prostate, Lung, Colorectal, and Ovarian Cancer Screening Trial is discussed above [[11]](#_ENREF_11). Included in this study are 286 African American prostate cancer cases and 269 controls without a history of prostate cancer, matched on age at randomization and study year of the trial.

*The Cancer Prevention Study II Nutrition Cohort (CPS-II):* The CPS-II Nutrition Cohort includes over 86,000 men and 97,000 women from 21 US states who completed a mailed questionnaire in 1992 (aged 40-92 years at baseline) [[12]](#_ENREF_12). Starting in 1997, follow-up questionnaires were sent to surviving cohort members every other year to update exposure information and to ascertain occurrence of new cases of cancer; a >90% response rate has been achieved for each follow-up questionnaire. From 1998-2001, blood samples were collected in a subgroup of 39,376 cohort members. To further supplement the DNA resources, during 2000-2001, buccal cell samples were collected by mail from an additional 70,000 cohort members. Incident cancers are verified through medical records, or through state cancer registries or death certificates when the medical record can not be obtained. Genomic DNA from 76 African American prostate cancer cases and 152 age-matched controls were included in stage 1 of the scan.

*Prostate Cancer Case-Control Studies at MD Anderson (MDA):* Participants in this study were identified from epidemiological prostate cancer studies conducted at the University of Texas M.D. Anderson Cancer Center in the Houston Metropolitan area since 1996. Cases were accrued from six institutions in the Houston Medical Center and were not restricted with respect to Gleason score, stage or PSA. Controls were identified via random-digit-dialing or among hospital visitors and they were frequency matched to cases on age and race. Lifestyle, demographic, and family history data were collected using a standardized questionnaire. These studies contributed 543 African American cases and 474 controls to this study [[13]](#_ENREF_13).

*The Los Angeles Study of Aggressive Prostate Cancer (LAAPC):* The LAAPC is a population-based case-control study of aggressive prostate among African Americans in Los Angeles County [[14]](#_ENREF_14). Cases were identified through the Los Angeles County Cancer Surveillance Program rapid case ascertainment system and eligible cases included African American men diagnosed with a first primary prostate cancer between January 1, 1999 and December 31, 2003. Eligible cases also had either tumor extension outside the prostate, metastatic prostate cancer in sites other than prostate, or needle biopsy of the prostate with Gleason grade 8 or higher, or Gleason grade 7 and tumor in more than 2/3 of the biopsy cores. Controls were identified by a neighborhood walk algorithm and were men never diagnosed with prostate cancer, and were frequency matched to cases on age (±5 years). For this study, genomic DNA was included for 296 cases and 140 controls. We also included an additional 163 African American controls from the MEC that were frequency matched to cases on age.

*Prostate Cancer Genetics Study (CaP Genes):* The African American component of this study population comprised 160 men: 75 cases diagnosed with more aggressive prostate cancer and 85 age-matched controls [[15]](#_ENREF_15). All subjects were recruited and frequency-matched on the major medical institutions in Cleveland, Ohio (i.e., the Cleveland Clinic, University Hospitals of Cleveland, and their affiliates) between 2001 and 2004. The cases were newly diagnosed with histologically confirmed disease: Gleason score 7; tumor stage T2c; or a prostate-specific antigen level >10 ng/ml at diagnosis. Controls were men without a prostate cancer diagnosis who underwent standard annual medical examinations at the collaborating medical institutions.

*Case-Control Study of Prostate Cancer among African Americans in Washington, DC (DCPC):* Unrelated men self-described as African American were recruited for several case-control studies on genetic risk factors for prostate cancer between the years 2001 and 2005 from the Division of Urology at Howard University Hospital (HUH) in Washington, DC. Control subjects unrelated to the cases and matched for age (± 5 years) were also ascertained from the prostate cancer screening population of the Division of Urology at HUH [[16]](#_ENREF_16). These studies included 292 cases and 359 controls.

*King County (Washington) Prostate Cancer Studies (KCPCS):* The study population consists of participants from one of two population-based case-control studies among residents of King County, Washington [17,18]. Incident Caucasian and African American cases with histologically confirmed prostate cancer were ascertained from the Seattle-Puget Sound SEER cancer registry during two time periods, 1993-1996 and 2002-2005. Age-matched (5-year age groups) controls were men without a self-reported history of being diagnosed with prostate cancer and were identified using one-step random digit telephone dialing. Controls were ascertained during the same time periods as the cases. A total of 145 incident African American cases and 81 African American controls were included from these studies.

*The Gene-Environment Interaction in Prostate Cancer Study (GECAP):* The Henry Ford Health System (HFHS) recruited cases diagnosed with adenocarcinoma of the prostate of Caucasian or African American race, less than 75 years of age, and living in the metropolitan Detroit tri-county area [[19]](#_ENREF_19). Controls were randomly selected from the same HFHS population base from which cases were drawn. The control sample was frequency matched at a ratio of 3 enrolled cases to 1 control based on race and five-year age stratum. In total, 637 cases and 244 controls were enrolled between January 2002 and December 2004.  Of study enrollees, DNA for 234 African Americans cases and 92 controls were included in stage 1 of the scan.

- 1. ***CARe cohorts***

African Americans as part of the NHLBI Candidate gene Association Resource (CARe) Project [[20]](#_ENREF_20) were surveyed. The CARe cohort consisted of five studies [[21]](#_ENREF_21).

*Atherosclerosis Risk in Communities (ARIC):* The ARIC study is a prospective population-based study of atherosclerosis and cardiovascular diseases in 15,792 men and women, including 11,478 non-Hispanic whites and 4,314 African Americans, drawn from 4 U.S. communities (suburban Minneapolis, Minnesota; Washington County, Maryland; Forsyth County, North Carolina, and Jackson, Mississippi). In the first three communities, the sample reflects the demographic composition of the community. In Jackson, only black residents were enrolled. Because of the design and focus of the CARe Project, only self-reported African-American participants are included in this analysis. Participants were between age 45 and 64 years at their baseline examination in 1987-1989 when blood was drawn for DNA extraction and participants consented to genetic testing. After taking into account availability of adequate amounts of high quality DNA, appropriate informed consent and genotyping quality control and assurance procedures, genotype data were available on 2,989 African-American individuals.

*Coronary Artery Risk Development in young Adults (CARDIA):* The CARDIA study is a prospective, multi-center investigation of the natural history and etiology of cardiovascular disease in African Americans and whites 18-30 years of age at the time of initial examination. The initial examination included 5,115 participants selectively recruited to represent proportionate racial, gender, age, and education groups from four communities: Birmingham, AL; Chicago, IL; Minneapolis, MN; and Oakland, CA. Participants from the Birmingham, Chicago, and Minneapolis centers were recruited from the total community or from selected census tracts. Participants from the Oakland center were randomly recruited from the Kaiser-Permanente health plan membership. Details of the study design have been published [[22]](#_ENREF_22). From the time of initiation of the study in 1985-1986, five follow-up examinations have been conducted at years 2, 5, 7, 10, 15, and 20. DNA extraction for genetic studies was performed at the Y10 examination. After taking into account availability of adequate amounts of high quality DNA, appropriate informed consent and genotyping quality control and assurance procedures, genotype data were available on 955 African-American individuals. Each participant’s age, race, and sex were self-reported during the recruitment phase and verified during the baseline clinic visit.

*Cleveland Family Study (CFS):* The Cleveland Family Study (CFS) is a family-based, longitudinal study designed to characterize the genetic and non-genetic risk factors for sleep apnea. In total, 2534 individuals (46% African American) from 352 families were studied on up to 4 occasions over a period of 16 years (1990-2006). The initial aim of the study was to quantify the familial aggregation of sleep apnea. Over time, the aims were expanded to characterize the natural history of sleep apnea, sleep apnea outcomes, and to identify the genetic basis for sleep apnea. With subsequent exams, the cohort was expanded to include increased minority representation and additional family members. The total sample included index probands (N=275) who were recruited from 3 area hospital sleep centers if they had a confirmed diagnosis of sleep apnea and at least 2 first-degree relatives available to be studied. In the first 5 years of the study, neighborhood control probands (N=87) with at least 2 living relatives available for study were selected at random from a list provided by the index family. All available first-degree relatives and spouses of the case and control probands were recruited. Second-degree relatives, including half-sibs, aunts, uncles and grandparents, were also included if they lived near the first degree relatives (cases or controls), or if the family had been found to have two or more relatives with sleep apnea. The sample, which is enriched for individuals with sleep apnea, also contains a high prevalence of individuals with sleep apnea-related traits, including: obesity, impaired glucose tolerance, and hypertension. Data that were used for the CARe analyses were for individuals for whom DNA had been collected (i.e., over the last two exam cycles (N=1447)). After genotyping quality control, genotype data were available for 632 African Americans. Phenotype data were collected over as many as 4 exam cycles, each occurring ~every 4 years. The last three exams targeted all subjects who had been studied at earlier exams, as well as new minority families and family members of previously studied probands who had been unavailable at prior exams. The phenotype data used in the current analysis were from the 4th exam (2001-06) conducted in 736 subjects, with oversampling of minorities and individuals in whom prior microsatellite genome scans had been conducted.

*Jackson Heart Study (JHS):* The Jackson Heart Study (JHS) is a prospective population-based study to seek the causes of the high prevalence of common complex diseases among African Americans in the Jackson, Mississippi metropolitan area, including cardiovascular disease, type-2 diabetes, obesity, chronic kidney disease, and stroke [[23]](#_ENREF_23). During the baseline examination period (2000-2004) 5,301 self-identified African Americans were recruited from four sources, including (1) randomly sampled households from a commercial listing; (2) ARIC participants; (3) a structured volunteer sample that was designed to mirror the eligible population; and (4) a nested family cohort. Unrelated participants were between 35 and 84 years old, and members of the family cohort were ≥ 21 years old when consent for genetic testing was obtained and blood was drawn for DNA extraction. Based on DNA availability, appropriate informed consent, and genotyping results that met quality control procedures, genotype data were available for 3,030 individuals, including 885 who are also ARIC participants. In the current study, JHS participants who were also enrolled in the ARIC study were analyzed with the ARIC dataset – for this reason, the JHS dataset analyzed here had 2,145 individuals.

*Multi-Ethnic Study of Atherosclerosis (MESA):* The Multi-Ethnic Study of Atherosclerosis (MESA) is a study of the characteristics of subclinical cardiovascular disease (disease detected non-invasively before it has produced clinical signs and symptoms) and the risk factors that predict progression to clinically overt cardiovascular disease or progression of the subclinical disease. MESA researchers study a diverse, population-based sample of 6,814 asymptomatic men and women aged 45-84 at baseline. Approximately 38% of the recruited participants are white, 28% African-American, 22% Hispanic, and 12% Asian, predominantly of Chinese descent. For the current study, after taking into account availability of adequate amounts of high quality DNA, appropriate informed consent and genotyping quality control and assurance procedures, genotype data were available on 1,646 African-American individuals. Participants were recruited from six field centers across the United States: Wake Forest University, Columbia University, Johns Hopkins University, University of Minnesota, Northwestern University and University of California - Los Angeles. Each participant received an extensive physical exam to determine coronary calcification, ventricular mass and function, flow-mediated endothelial vasodilation, carotid intimal-medial wall thickness and presence of echogenic lucencies in the carotid artery, lower extremity vascular insufficiency, arterial wave forms, electrocardiographic (ECG) measures, standard coronary risk factors, socio-demographic factors, lifestyle factors, and psychosocial factors. Selected repetition of subclinical disease measures and risk factors at follow-up visits allows study of the progression of disease. Blood samples are being assayed for putative biochemical risk factors and stored for case-control studies. DNA is extracted and lymphocytes immortalized for study of candidate genes and genome-wide scanning. Participants are followed for identification and characterization of cardiovascular disease events, including acute myocardial infarction and other forms of coronary heart disease (CHD), stroke, and congestive heart failure; for cardiovascular disease interventions; and for mortality. In addition to the six Field Centers, MESA involves a Coordinating Center, a Central Laboratory, and Central Reading Centers for Computed Tomography (CT), Magnetic Resonance Imaging (MRI), Ultrasound, and Electrocardiography (ECG). Protocol development, staff training, and pilot testing were performed in the first 18 months of the study. The first examination took place over two years, from July 2000 - July 2002. It was followed by three additional examination periods: September 2002 – January 2004, February 2004 - July 2005 September 2005 - May 2007. Participants are contacted every 9 to 12 months throughout the study to assess clinical morbidity and mortality. NHLBI recently funded MESA II, which will bring all participants back for a fifth exam, starting in April 2010.

- 1. ***Maywood cohort***

DNA samples were obtained from African Americans enrolled in studies of blood pressure at Loyola University in Maywood, IL, USA. A total of 775 participants from 311 families were surveyed [[24]](#_ENREF_24). Phenotype measurements were performed using a standardized protocol [[25]](#_ENREF_25).

- 1. ***Nigerian cohort***

Participants of the Nigerian cohort were recruited from Igbo-Ora and Ibadan in southwest Nigeria as a long-term study on the environmental and genetic factors underlying hypertension [[26]](#_ENREF_26). A total of 1,188 unrelated adults were evaluated in this analysis. Phenotype measurements were performed using a standardized protocol [[25]](#_ENREF_25).

1. **Replication cohorts**

Replication results for the top 153 SNP associations were performed *in silico*, using data from five cohorts.

- 1. ***The Healthy Aging in Neighborhoods of Diversity across the Life Span study (HANDLS) study***

The HANDLS is an interdisciplinary, community-based, prospective longitudinal epidemiologic study examining the influences of race and socioeconomic status (SES) on the development of age-related health disparities among socioeconomically diverse African Americans and whites in Baltimore. A total of 3,722 participants were recruited from Baltimore, MD with mean age 47.7 (range 30-64) years.

- 1. ***Health, Aging, and Body Composition (Health ABC) Study***

The Health ABC study is a prospective cohort study investigating the associations between body composition, weight-related health conditions, and incident functional limitation in older adults. Health ABC enrolled well-functioning, community-dwelling black (N=1281) and white (N=1794) men and women aged 70-79 years between April 1997 and June 1998. Participants were recruited from a random sample of white and all black Medicare eligible residents in the Pittsburgh, PA, and Memphis, TN, metropolitan areas. Participants have undergone annual exams and semi-annual phone interviews.

- 1. ***GeneSTAR study***

Participants came from GeneSTAR (Genetic Study of Atherosclerosis Risk), an ongoing prospective study begun in 1983 designed to determine environmental, phenotypic, and genetic causes of premature cardiovascular disease. Participants came from families identified from 1983-2006 from probands with a premature coronary disease event prior to 60 years of age who were identified at the time of hospitalization in any of 10 Baltimore area hospitals. Their apparently healthy 30-59 year old siblings without known CAD were recruited and underwent phenotypic measurement and characterization between 1983 and 2006. From 2003-2006, adult offspring over 21 years of age of all participating siblings and probands and the coparent of the offspring were recruited and underwent phenotypic measurement and characterization. Height was measured using a stadiometer with participants wearing no shoes. Genotyping was performed on the Illumina 1Mv1_c platform.

- 1. ***Women Health Initiative (WHI)***

The Women’s Health Initiative (WHI) SNP Health Association Resource (SHARe) minority cohort included self-identified African American and Hispanic women from the Women’s Health Initiative Study [[27]](#_ENREF_27) who provided consent for genetic research. A total of 8,515 African American women were genotyped. Body height (in cm) was measured using wall-mounted stadiometers. Baseline height was used for all women with the exception of 41 women who were missing baseline values but had height measurements from a follow-up visit (all within 3 years of baseline). Six women without any height data were excluded from analyses. At the request of dbGAP, body height measurements were truncated for the 1^st^ and 99^th^ percentiles (N=157). This truncation was requested by dbGAP to retain anonymity for SHARe participants. We retained these truncated values in the analyses.

- 1. ***Multiethnic Cohort (MEC).***

Details of the MEC are provided above. As part of the replication phase we genotyped rs12393627 in the MEC samples that are part of AABC and AAPC as well as additional African American samples in the MEC from a study of type II diabetes [[28]](#_ENREF_28) and Latinos and Japanese samples from case-control studies of breast (BC) and prostate (PC) cancer in the MEC [29,30]. As in the GWAS, excluded from the analysis were subjects with height values >± 2 SD from the mean. Genotypes at rs12393627 were provided for 5,103 African Americans, 2,979 Latinos and 3,487 Japanese. For the Latino samples, principal components were available from ongoing GWAS for these cancers (illumina 660W). For the African American T2D study, principal components were estimated from genotyping of >100 ancestry informative markers (AIMs) [[31]](#_ENREF_31).

1. **Genotyping**

Genotyping and quality control steps in the CARe cohorts (ARIC, CFS, CARDIA, JHS, MESA), Maywood, Nigeria, GeneSTAR, and HABC have been described elsewhere [21,32,33].

***3.1 AABC***

Genotyping in AABC was conducted using the Illumina Human1M-Duo BeadChip. Of the 5,984 samples from these studies (3,153 cases and 2,831 controls), we attempted genotyping of 5,932, removing samples (n=52) with DNA concentrations <20 ng/ul. Following genotyping, we removed samples based on the following exclusion criteria: 1) unknown replicates (≥98.9% genetically identical) that we were able to confirm (only one of each duplicate was removed, n=15); 2) unknown replicates that we were not able to confirm through discussions with study investigators (pair or triplicate removed, n=14); 3) samples with call rates <95% after a second attempt (n=100); 4) ancestry outliers (n=36); and, 5) samples with <15% mean heterozygosity of SNPs in the X chromosome and/or similar mean allele intensities of SNPs on the X and Y chromosomes (n=6) (these are likely to be males).

In the analysis, we removed SNPs with <95% call rate (n=21,732) or minor allele frequencies (MAFs) <1% (n=80,193). To assess genotyping reproducibility we included 138 replicate samples; the average concordance rate was 99.95% (>99.93% for all pairs). We also eliminated SNPs with genotyping concordance rates <98% based on the replicates (n=11,701). The final analysis dataset included 1,043,036 SNPs genotyped on 3,016 breast cancer cases and 2,745 controls, with an average SNP call rate of 99.7% and average sample call rate of 99.8%. Hardy-Weinberg equilibrium (HWE) was not used as a criterion for removing SNPs at this stage; all SNPs reported in this report did not deviate from HWE (based on a cut-off of *P*<1x10^-6^).

*Global Ancestry Estimation.* We utilized STRUCTURE to infer percent African ancestry on an individual level. Individuals with ≤ 5% African ancestry were excluded. We removed individuals missing height and individuals with height Z-scores >2 or <-2. The GWAS analysis of height in AABC included 5,380 subjects.

***3.2 AAPC***

Genotyping in AAPC was conducted using the Illumina Infinium 1M-Duo bead array at the University of Southern California and the NCI Genotyping Core Facility (PLCO study). Following genotyping, samples were removed based on the following exclusion criteria: 1) unknown replicates across studies (n=24, none within studies); 2) call rates <95% (n=126); 3) >10% mean heterozygosity on the X chromosome and/or <10% mean intensity on the Y chromosome - we inferred 3 samples to be XX and 6 to be XXY; and, 4) ancestry outliers and related samples (n=249). To assess genotyping reproducibility we included 158 replicate samples; the average concordance rate was 99.99% (≥99.3% for all pairs). Starting with 1,153,397 SNPs, we removed SNPs with <95% call rate, MAFs <1%, or >1 QC mismatch based on sample replicates (n=105,411). All SNPs reported in this report did not deviate from HWE (based on a cut-off of *P*<1x10^-6^).

*Global Ancestry Estimation.* A total of 2,546 ancestry-informative SNPs from the Illumina array were selected based on low inter-marker correlation and ability to differentiate between samples of African and European descent. An individual was subject to filtering from the analysis if his value along eigenvector 1 or 2 was outside of 4 standard deviations of the mean of each respective eigenvector, based on principal components analysis. We also removed individuals missing height and individuals with height Z-scores >2 or <-2. The GWAS analysis of height in AAPC included 5,526 subjects.

***3.3 HANDLS***

Genotyping was successfully performed on 1,024 African American participants. Initial inclusion criteria for genetic data includes concordance between self reported sex and sex estimated from X chromosome heterogeneity, > 95% call rate per participant (across all equivalent arrays), concordance between self-reported African ancestry and ancestry confirmed by analyses of genotyped SNPs, and no cryptic relatedness to any other samples at a level of proportional sharing of genotypes > 15% (effectively excluding 1^st^ cousins and closer relatives from the set of probands used in analyses). In addition, SNPs were filtered for HWE p-value > 1e-7, missing by haplotype p-values > 1e-7, minor allele frequency > 0.01, and call rate > 95%. Basic genotype quality control and data management was conducted using PLINKv1.06 [[34]](#_ENREF_34). Cryptic relatedness was estimated via pairwise identity by descent analyses in PLINK and confirmed using RELPAIR[[35]](#_ENREF_35).

Ancestry estimates were assessed using both STRUCTUREv2.3[[36-38]](#_ENREF_36) and the multidimensional scaling (MDS) function in PLINKv1.06. In the multidimensional scaling analysis, HANDLS participants were clustered with data made available from HapMap Phase 3 for the YRI, ASW, CEU, TSI, JPT and CHB populations, using a set of 36892 linkage-disequilibrium-pruned SNPs common to each population. This set of SNPs were chosen as they are not in r^2^ > 0.20 with another SNP in overlapping sliding windows of 100 SNPs in the ASW samples. HANDLS participants with component vector estimates consistent with the HapMap ASW samples for the first 4 component vectors were included. In addition, the 1,024 quality controlled HANDLS samples were later clustered among themselves using MDS to generate 10 component vectors estimating internal population structure within the HANDLS study. Of the SNPs utilized for MDS clustering, the 2000 SNPs with the most divergent allele frequency estimates between African populations (frequency estimates based on YRI samples) and European populations (frequency estimates based on combined CEU and TSI samples) were utilized as ancestry informative markers (AIMs). These 2000 AIMs were associated with frequency differences on the level of p-values < 1e-3 based on chi-squared tests. A two population model in STRUCTURE was used to estimate percent African and percent European ancestry in the HANDLS samples, for a 10000 iteration burn-in period, and a 10000 iteration follow-up of the Markov Chain Monte Carlo model utilized by STRUCTURE. The ancestry estimates from STRUCTURE were highly concordant with the first component vector of the MDS clustering of HANDLS samples, with an r^2^ of > 0.82.

***3. 4*** ***WHI***

Genetic data were obtained from genome-wide scans using the Genome-Wide Human SNP Array 6.0 containing 909,622 single nucleotide polymorphisms (SNPs). Genotyping QC included examination of concordance rates for blinded and un-blinded duplicates. SNPs with call rates < 95% or extreme departures from Hardy Weinberg proportions, p<10^–7^were excluded. After quality control, 855,034 SNPs were available for the association analysis. Genotyping failed in approximately 1% (n=99) samples. Additional participants were excluded based on low call rates <95%, race/ethnicity discrepancy (n=57), and relatedness [39,40]. The first or second degree relative with the highest call rate was retained and other family members were excluded, n=208, leaving a total of 8,149 African American women for analysis.

**3.5 Multiethnic Cohort (MEC)**

Genotyping of rs12393627 in the MEC samples as part of AABC and AAPC as well as the additional African American, Latino and Japanese samples was performed using the TaqMan allelic discrimination assay. The concordance of blinded replicates was 100%.

1. **Statistical methods**
   1. ***Genotype imputation***

Imputation was performed using MACH 1.0.16 (<http://www.sph.umich.edu/csg/abecasis/MaCH/>). MACH requires phased reference haplotypes to perform imputation. For the African Americans, a combined CEU+YRI reference panel was created. This panel includes SNPs segregating in both CEU and YRI, as well as SNPs segregating in one panel and monomorphic and nonmissing in the other (2.74 million altogether). Due to the overlap of African American individuals on the Affymetrix 6.0 and IBC arrays [23,41], it was possible to analyze imputation performance at SNPs not genotyped on Affymetrix 6.0. For imputation based on Affymetrix data, the use of the CEU+YRI panel resulted in an allelic concordance rate of ~95.6%, calculated as 1 – 1/2*|imputed_dosage – chip_dosage|. This rate is comparable to rates calculated for individuals of African descent imputed with the HapMap 2 YRI individuals[[1]](#_ENREF_1). For each imputed sample, imputation was performed in two steps. For the first step, individuals with pedigree relatedness or cryptic relatedness (pi_hat > 0.05) were filtered. A subset of individuals was randomly extracted from each panel and used to generate recombination and error rate estimates for the corresponding sample. In the second step, these rates were used to impute all sample individuals across the entire reference panel. Imputation results were filtered at an RSQ_HAT threshold of 0.3 and a minor allele frequency threshold of 0.01.

- 1. ***Trait modeling***

Height measures were obtained for all cohorts during participants’ visits, except for AABC and AAPC were it was self-reported. For individuals with multiple height measurements available, we used the first height measure recorded. In all studies but Nigeria, individuals younger than 18 years old or older than 85 years old were excluded. In the Nigeria cohort (N=1,188), four individuals younger (age range: 16-17) and three individuals older than 85 (age range: 89-95) were kept in the analysis; exclusion of these samples will not affect the overall results of our meta-analysis of >20,000 participants. We stratified (when applicable) by sex and case-control status the studies. We corrected for age and other appropriate covariates (*e.g.* recruitment center), and then normalized the height residuals into height Z-scores. The height Z-scores were combined across gender and disease status, and the normality of the data was inspected by drawing a histogram. Outliers (Z-scores >4 or <-4) were excluded from the analysis (AABC and AAPC excluded individuals with height Z-scores >2 or <-2).

- 1. ***Association testing***

We tested association between genotyped or imputed SNPs and height Z-scores using a linear regression framework and an additive genetic model. Analysis were performed using PLINK [[34]](#_ENREF_34), mach2qtl, R, or a linear mixed effect model [[42]](#_ENREF_42) to account for family structure (CFS and GeneSTAR). We used the 10 first principal components as covariates to control for admixture and stratification. For conditional analysis, we included in the same linear regression model the SNP of interest.

- 1. ***Meta-analysis***

A total of 3,310,998 SNPs were meta-analyzed across 9 studies (ARIC, CARDIA, CFS, JHS, Maywood, MESA, Nigeria, AABC, and AAPC). We performed a quality control in each cohort, and only SNPs meeting the following criteria were included in the analysis: (i) minor allele frequency ≥ 0.01, (ii) MAC (sample size multiplied by minor allele frequency) ≥ 5, and (iii) oevar_imp (imputation quality) ≥ 0.3 for imputed SNPs. The genomic control inflation (λ_GC_) factor [43,44] was calculated for each study and used for within-study correction, prior to the meta-analysis. We used the inverse-variance fixed effects meta-analysis method to combine the results from individual studies. The overall GC factor (λ_GC_) was 1.064, suggesting no evidence of inflation. Meta-analysis was performed using the software program METAL ([www.sph.umich.edu/csg/abecasis/metal](http://www.sph.umich.edu/csg/abecasis/metal)) [[45]](#_ENREF_45). From the meta-analysis, we selected 153 SNPs that reached *P* < 1x10^-5^ in the meta-analysis for replication in four additional cohorts (n = 11,429). Association results from the discovery and replication studies were combined using the same inverse variance approach.

- 1. ***Conditional analyses***

To test for the presence of additional independently associated SNPs near rs4315565 on chromosome 2 and rs12393627 on the X chromosome, we included the genotype (as a dose for imputed SNP (0.0-2.0) or integer for genotyped SNP (0,1,2)) in the same regression model and repeated our linear regression analyses in a 1 Mb window centered around these top index SNPs.

- 1. ***Local ancestry estimates***

We used the software HAPMIX [[46]](#_ENREF_46) to estimate local ancestry on autosomes at each of the genotyped markers in the AAPC, AABC, and CARe cohorts. HAPMIX uses a reference panel (ancestral populations – in our case the HapMap YRI and CEU datasets) to assign at each locus in an admixed population (African American) the number of chromosomes (0, 1, or 2) that are of European or African descent. Since rs4315565 is an imputed marker, for the conditional and stratified analyses, we used estimates of local ancestry at the physically closest genotyped SNP.

**5. Analysis of allelic expression phenotypes**

**5.1 *Samples and Cell culture***

Our cohort included 60 unrelated YRI HapMap samples from which 56 were successfully grown (all phase 1 and 2 samples except GM18862, GM19116, GM19152, GM19153). Lymphoblastoid cell lines (LCLs) were cultured in RPMI 1640 media with 2mM L-glutamine, 15% fetal bovine serum, and 1% penicillin-streptomycin at 37°C with 5% CO_2_.

**5.2** **RNA *and DNA extraction and ds-cDNA synthesis***

We extracted total RNA and synthesized double-stranded cDNA as previously described [[47]](#_ENREF_47). Briefly, approximately 150 μg of total RNA was isolated and treated with 6 U DNase I and poly(A). The mRNA was purified using the MicroPoly(A)Purist protocol (Ambion Inc.) and cDNA synthesis was performed using the Superscript Double-Stranded cDNA Synthesis Kit (Invitrogen). DNA from the cell lysates was extracted using GenElute DNA Miniprep Kit (Sigma Aldrich) and resuspended in 200µl PBS.

**5.3 *Genotyping and allelic expression analysis on Human1M-Duo Beadchips***

Approximately 200 ng of genomic DNA and 50–300 ng of double-stranded cDNA were used for the parallel genotyping and allelic expression analysis on the Illumina Human Human1M-Duo according to the manufacturer’s instructions (Illumina Inc.). Genotypes were extracted using BeadStudio. The parallel assessment of genomic DNA and cDNA heterozygote ratios was then carried out to observe allelic expression differences among heterozygous expressed SNPs [[47]](#_ENREF_47). The allele ratio skewing caused by differences in signal intensities between genomic DNA and cDNA was corrected by applying a polynomial regression model as previously described [[48]](#_ENREF_48).

Mapping of SNPs underlying population differences in YRI allelic expression was carried out using a similar approach as previously described [[47]](#_ENREF_47) although now allelic differences were only mapped for full length annotated transcripts where we observed expression of SNPs in cDNA samples. Briefly, all SNP locations and gene annotations are based on NCBI build 36. HapMap genotypes were retrieved from HapMap release 23. To identify full length transcripts, we applied three gene annotations from UCSC database HG18: RefSeq gene (refFlattable ), UCSC gene (knownGene table) and Vega gene (vegaGene table). In order to map *cis*-regulatory SNPs, we first calculated the mean of normalized allelic expression across annotated genes for each sample, using one of the phased chromosomes (haplotypes) as reference. Then, this averaged allelic expression was used in a linear regression test, with the averaged allelic expression as the dependent variable and the phased genotype as the predictor variable. In the regression test, we assigned phased genotype AB as value 0, BB as 2 and heterozygous AB genotype as 1. All SNPs in 250 kb flanking the gene or within the gene were tested. Any height SNPs observed in earlier meta-analysis [[49]](#_ENREF_49) or in African-ancestry populations and within 250 kb of a measured transcript was then overlapped with the allelic expression mapping data generated in YRI LCLs.

**5.4 *Statistical analysis***

In order to assess empirical significance of the overlap of height SNPs with our genome-wide allelic expression mapping data in YRI LCLs, we carried out permutation tests using closely matched selections of SNPs. Briefly, we generated random matched pairs of SNPs using HapMap CEU and YRI genotyping and linkage disequilibrium information. To achieve variant matching, we applied the following criteria: 1) Minor allele frequency (MAF): MAF difference between random SNPs (based on CEU data) and height SNPs (based on European meta-analysis) was less than 0.05. 2) Distance from gene: The difference in distance from CEU SNPs and height SNPs to the closest annotated gene was less than 1 kb. 3) Linkage disequilibrium (LD): Based on SNPs selected in CEU and fulfilling above criteria, we selected SNPs in YRI that showed similar *r*^2^ as what we observed for top height SNPs. Specifically, this LD difference was less than 0.025 in HapMap YRI phased genotypes. If the SNPs in CEU were not genotyped in YRI (thus, no *r*^2^ information available), we just used distance between two SNPs in a pair as selection criteria. Using all three matching conditions, we generated 100 sets of 161 SNPs randomly selected from chromosomes 1 to 22.

**References**

1. Kolonel LN, Henderson BE, Hankin JH, Nomura AMY, Wilkens LR, et al. (2000) A Multiethnic Cohort in Hawaii and Los Angeles: Baseline Characteristics. American Journal of Epidemiology 151: 346-357.

2. Polly AM, Jill AM, Hoyt GW, Nancy MB, Janet RD, et al. (2002) The NICHD Women's Contraceptive and Reproductive Experiences Study: Methods and Operational Results. Annals of epidemiology 12: 213-221.

3. Ambrosone CB, Ciupak GL, Bandera EV, Jandorf L, Bovbjerg DH, et al. (2009) Conducting Molecular Epidemiological Research in the Age of HIPAA: A Multi-Institutional Case-Control Study of Breast Cancer in African-American and European-American Women. J Oncol 2009: 871250.

4. John EM, Schwartz GG, Koo J, Wang W, Ingles SA (2007) Sun exposure, vitamin D receptor gene polymorphisms, and breast cancer risk in a multiethnic population. Am J Epidemiol 166: 1409-1419.

5. John EM, Miron A, Gong G, Phipps AI, Felberg A, et al. (2007) Prevalence of pathogenic BRCA1 mutation carriers in 5 US racial/ethnic groups. JAMA 298: 2869-2876.

6. Newman B, Moorman PG, Millikan R, Qaqish BF, Geradts J, et al. (1995) The Carolina Breast Cancer Study: integrating population-based epidemiology and molecular biology. Breast Cancer Res Treat 35: 51-60.

7. Prorok PC, Andriole GL, Bresalier RS, Buys SS, Chia D, et al. (2000) Design of the Prostate, Lung, Colorectal and Ovarian (PLCO) Cancer Screening Trial. Control Clin Trials 21: 273S-309S.

8. Zheng W, Cai Q, Signorello LB, Long J, Hargreaves MK, et al. (2009) Evaluation of 11 breast cancer susceptibility loci in African-American women. Cancer Epidemiol Biomarkers Prev 18: 2761-2764.

9. Smith TR, Levine EA, Freimanis RI, Akman SA, Allen GO, et al. (2008) Polygenic model of DNA repair genetic polymorphisms in human breast cancer risk. Carcinogenesis 29: 2132-2138.

10. Signorello LB, Hargreaves MK, Steinwandel MD, Zheng W, Cai Q, et al. (2005) Southern community cohort study: establishing a cohort to investigate health disparities. J Natl Med Assoc 97: 972-979.

11. Gohagan JK, Prorok PC, Hayes RB, Kramer BS (2000) The Prostate, Lung, Colorectal and Ovarian (PLCO) Cancer Screening Trial of the National Cancer Institute: history, organization, and status. Control Clin Trials 21: 251S-272S.

12. Calle EE, Rodriguez C, Jacobs EJ, Almon ML, Chao A, et al. (2002) The American Cancer Society Cancer Prevention Study II Nutrition Cohort: rationale, study design, and baseline characteristics. Cancer 94: 500-511.

13. Strom SS, Gu Y, Zhang H, Troncoso P, Babaian RJ, et al. (2004) Androgen receptor polymorphisms and risk of biochemical failure among prostatectomy patients. The Prostate 60: 343-351.

14. Ingles SA, Coetzee GA, Ross RK, Henderson BE, Kolonel LN, et al. (1998) Association of prostate cancer with vitamin D receptor haplotypes in African-Americans. Cancer Res 58: 1620-1623.

15. Liu X, Plummer SJ, Nock NL, Casey G, Witte JS (2006) Nonsteroidal antiinflammatory drugs and decreased risk of advanced prostate cancer: modification by lymphotoxin alpha. Am J Epidemiol 164: 984-989.

16. Robbins, Christiane, Benn T, Jada, Hooker, et al. (2007) Confirmation study of prostate cancer risk variants at 8q24 in African Americans identifies a novel risk locus. Woodbury, NY, ETATS-UNIS: Cold Spring Harbor Laboratory Press. 6 p.

17. Agalliu I, Salinas CA, Hansten PD, Ostrander EA, Stanford JL (2008) Statin Use and Risk of Prostate Cancer: Results from a Population-based Epidemiologic Study. American Journal of Epidemiology 168: 250-260.

18. Stanford JL, Wicklund KG, McKnight B, Daling JR, Brawer MK (1999) Vasectomy and risk of prostate cancer. Cancer Epidemiol Biomarkers Prev 8: 881-886.

19. Rybicki BA, Neslund-Dudas C, Nock NL, Schultz LR, Eklund L, et al. (2006) Prostate cancer risk from occupational exposure to polycyclic aromatic hydrocarbons interacting with the GSTP1 Ile105Val polymorphism. Cancer Detect Prev 30: 412-422.

20. Musunuru K, Lettre G, Young T, Farlow DN, Pirruccello JP, et al. (2010) Candidate Gene Association Resource (CARe) / Clinical Perspective. Circulation: Cardiovascular Genetics 3: 267-275.

21. Lettre G, Palmer CD, Young T, Ejebe KG, Allayee H, et al. (2011) Genome-Wide Association Study of Coronary Heart Disease and Its Risk Factors in 8,090 African Americans: The NHLBI CARe Project. PLoS Genet 7: e1001300.

22. Friedman GD, Cutter GR, Donahue RP, Hughes GH, Hulley SB, et al. (1988) CARDIA: study design, recruitment, and some characteristics of the examined subjects. J Clin Epidemiol 41: 1105-1116.

23. Taylor HA, Jr., Wilson JG, Jones DW, Sarpong DF, Srinivasan A, et al. (2005) Toward resolution of cardiovascular health disparities in African Americans: design and methods of the Jackson Heart Study. Ethn Dis 15: S6-4-17.

24. Hassanein MT, Lyon HN, Nguyen TT, Akylbekova EL, Waters K, et al. (2010) Fine mapping of the association with obesity at the FTO locus in African-derived populations. Human Molecular Genetics 19: 2907-2916.

25. Cooper R, Puras A, Tracy J, Kaufman J, Asuzu M, et al. (1997) Evaluation of an electronic blood pressure device for epidemiological studies. Blood Press Monit 2: 35-40.

26. Kang SJ, Chiang CWK, Palmer CD, Tayo BO, Lettre G, et al. (2010) Genome-wide association of anthropometric traits in African- and African-derived populations. Hum Mol Genet 19: 2725-2738.

27. (1998) The Women's Health Initiative Study Group. Design of the Women's Health Initiative clinical trial and observational study. Control Clin Trials 19: 61-109.

28. Ge B, Gurd S, Gaudin T, Dore C, Lepage P, et al. (2005) Survey of allelic expression using EST mining. Genome Res 15: 1584-1591.

29. Chen F, Stram DO, Le Marchand L, Monroe KR, Kolonel LN, et al. (2011) Caution in generalizing known genetic risk markers for breast cancer across all ethnic/racial populations. Eur J Hum Genet 19: 243-245.

30. Pastinen T, Sladek R, Gurd S, Sammak A, Ge B, et al. (2004) A survey of genetic and epigenetic variation affecting human gene expression. Physiol Genomics 16: 184-193.

31. Kosoy R, Nassir R, Tian C, White PA, Butler LM, et al. (2009) Ancestry informative marker sets for determining continental origin and admixture proportions in common populations in America. Hum Mutat 30: 69-78.

32. Kang SJ, Chiang CW, Palmer CD, Tayo BO, Lettre G, et al. (2010) Genome-wide association of anthropometric traits in African- and African-derived populations. Hum Mol Genet 19: 2725-2738.

33. Johnson AD, Yanek LR, Chen MH, Faraday N, Larson MG, et al. (2010) Genome-wide meta-analyses identifies seven loci associated with platelet aggregation in response to agonists. Nat Genet 42: 608-613.

34. Purcell S, Neale B, Todd-Brown K, Thomas L, Ferreira M, et al. (2007) PLINK: a toolset for whole-genome association and population-based linkage analysis. American Journal of Human Genetics 81.

35. Epstein MP, Duren WL, Boehnke M (2000) Improved inference of relationship for pairs of individuals. Am J Hum Genet 67: 1219-1231.

36. Pritchard JK, Stephens M, Donnelly P (2000) Inference of population structure using multilocus genotype data. Genetics 155: 945-959.

37. Falush D, Stephens M, Pritchard JK (2003) Inference of population structure using multilocus genotype data: linked loci and correlated allele frequencies. 164: 1567-1587.

38. Falush D, Stephens J, Pritchard JK (2007) Inference of population structure using multilocus genotype data: dominant markers and null alleles. Molecular Ecology Notes 7: 574-578.

39. Purcell S, Neale B, Todd-Brown K, Thomas L, Ferreira MA, et al. (2007) PLINK: a tool set for whole-genome association and population-based linkage analyses. Am J Hum Genet 81: 559-575.

40. Thornton T, McPeek MS (2010) ROADTRIPS: case-control association testing with partially or completely unknown population and pedigree structure. Am J Hum Genet 86: 172-184.

41. Warnick GR (1986) Enzymatic methods for quantification of lipoprotein lipids. Methods Enzymol 129: 101-123.

42. Chen MH, Yang Q (2010) GWAF: an R package for genome-wide association analyses with family data. Bioinformatics 26: 580-581.

43. Clayton DG, Walker NM, Smyth DJ, Pask R, Cooper JD, et al. (2005) Population structure, differential bias and genomic control in a large-scale, case-control association study. Nat Genet 37: 1243-1246.

44. Devlin B, Roeder K, Wasserman L (2001) Genomic Control, a New Approach to Genetic-Based Association Studies. Theoretical Population Biology 60: 155-166.

45. Willer CJ, Li Y, Abecasis GaR (2010) METAL: fast and efficient meta-analysis of genomewide association scans. Bioinformatics 26: 2190-2191.

46. Price AL, Tandon A, Patterson N, Barnes KC, Rafaels N, et al. (2009) Sensitive detection of chromosomal segments of distinct ancestry in admixed populations. PLoS Genet 5: e1000519.

47. Ge B, Pokholok DK, Kwan T, Grundberg E, Morcos L, et al. (2009) Global patterns of cis variation in human cells revealed by high-density allelic expression analysis. Nat Genet 41: 1216-1222.

48. Grundberg E, Adoue V, Kwan T, Ge B, Duan QL, et al. (2011) Global analysis of the impact of environmental perturbation on cis-regulation of gene expression. PLoS Genet 7: e1001279.

49. Lango Allen H, Estrada K, Lettre G, Berndt SI, Weedon MN, et al. (2010) Hundreds of variants clustered in genomic loci and biological pathways affect human height. Nature 467: 832-838.
