# Supplementary figures and images for: Correction: Identification, Replication, and Fine-Mapping of Loci Associated with Adult Height in Individuals of African Ancestry
Source: PLoS Genet. 2011 Nov 21;7(11):10.1371/annotation/58c67154-3f10-4155-9085-dcd6e3689008. doi: 10.1371/annotation/58c67154-3f10-4155-9085-dcd6e3689008 (PMC3227698; doi:10.1371/annotation/58c67154-3f10-4155-9085-dcd6e3689008)

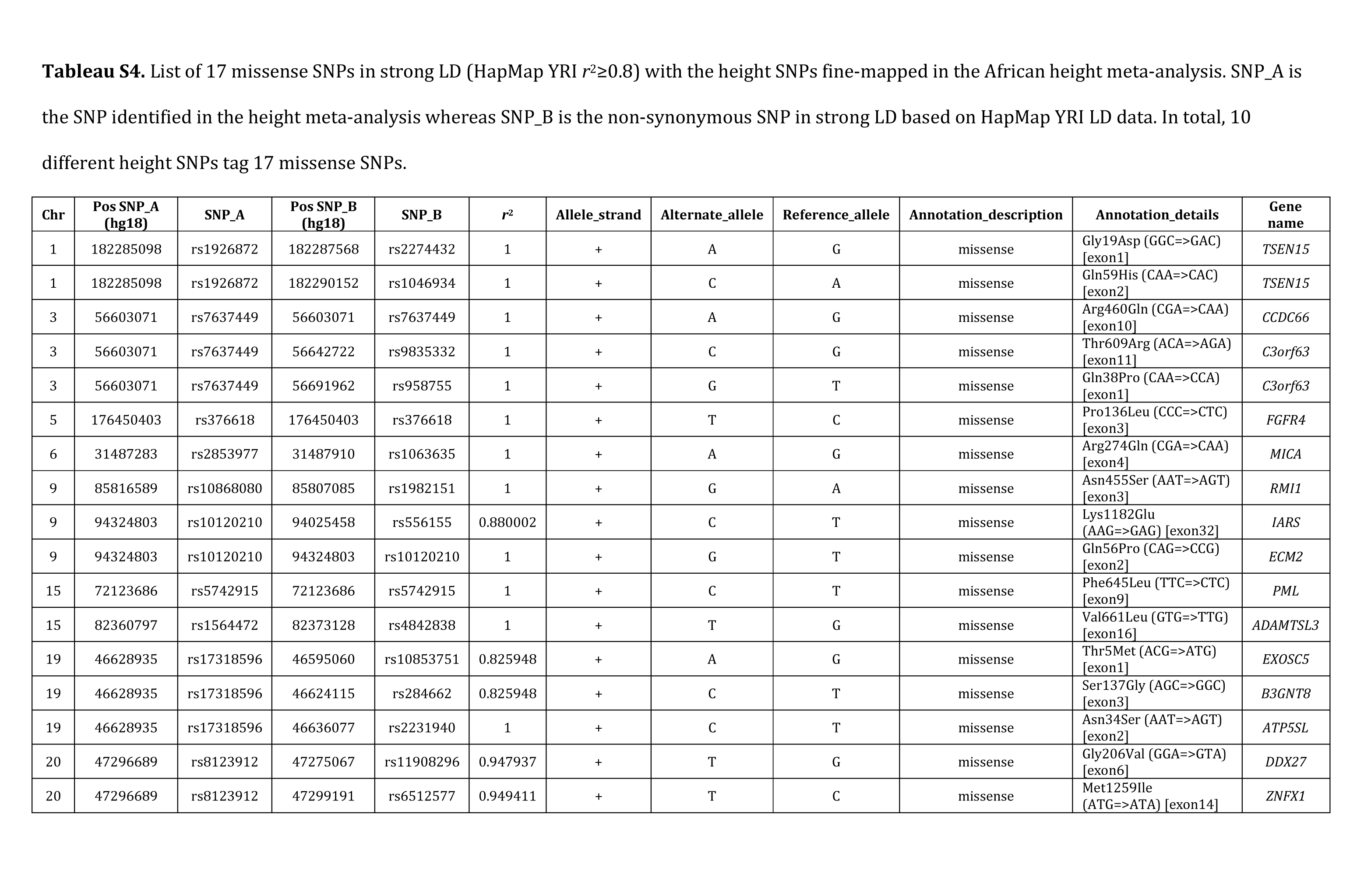

Supplement: Supplementary file 1 [file pgen.58c67154-3f10-4155-9085-dcd6e3689008.s001.tif]
